# Effects of Physical Activity Interventions on Strength, Balance and Falls in Middle-Aged Adults: A Systematic Review and Meta-Analysis

**DOI:** 10.1186/s40798-023-00606-3

**Published:** 2023-07-19

**Authors:** Michael Adams, Katharina Gordt-Oesterwind, Martin Bongartz, Samuel Zimmermann, Svenja Seide, Volker Braun, Michael Schwenk

**Affiliations:** 1 Network Aging Research, Heidelberg University, 69115 Heidelberg, Germany; 2 Institute of Sports and Sports Sciences, Heidelberg University, 69117 Heidelberg, Germany; 3 Unit Digitale Geriatrie, Geriatric Center of Heidelberg University Hospital, Medical Faculty of Heidelberg University, 69120 Heidelberg, Germany; 4 Geriatric Center, Heidelberg University Hospital, Agaplesion Bethanien Hospital Heidelberg, 69126 Heidelberg, Germany; 5 Institute of Medical Biometry, Heidelberg University Hospital, 69120 Heidelberg, Germany; 6 Medical Faculty Mannheim, Heidelberg University, 68167 Mannheim, Germany; 7 Human Performance Research Centre, Department of Sport Science, University of Konstanz, 78464 Constance, Germany

**Keywords:** Middle age, Middle-aged, Prevention, Strength, Balance, Falls, Exercise, Physical activity, Review

## Abstract

**Background:**

Weak lower body strength and balance impairments are fundamental risk factors for mobility impairments and falls that can be improved by physical activity (PA). Previous meta-analyses have focused on these risk factors in adults aged ≥ 65 years. Yet, the potential of PA for improving these risk factors in middle-aged populations has not been systematically investigated. This systematic review and meta-analysis aim to examine the effect of general and structured PA on lower limb strength, postural balance and falls in middle-aged adults.

**Methods:**

A computerized systematic literature search was conducted in the electronic databases MEDLINE, CINAHL, Web of Science and Cochrane Library. PA intervention types were classified according to the ProFaNE taxonomy. Randomized controlled trials exploring the effects of PA on strength (e.g., leg press one-repetition-maximum), balance (e.g., single limb stance) and falls (e.g., fall rates) in adults aged 40–60 years were systematically searched and included in a network analysis. Moderator analyses were performed for specific subgroups (age, sex, low PA). The methodological quality of the included studies was assessed using the Physiotherapy Evidence Database (PEDro) Scale.

**Results:**

Out of 7170 articles screened, 66 studies (median PEDro score 5) with 3387 participants were included. Strong, significant effects on muscle strength were found for strength (SMD = 1.02), strength–aerobic (SMD = 1.41), strength–endurance (SMD = 0.92) and water-based (SMD = 1.08) training (52 studies, *I*^2^ = 79.3%). Strength training (SMD = 1.16), strength–aerobic (SMD = 0.98) and 3D training (SMD = 1.31) improved postural balance (30 studies, *I*^2^ = 88.1%). Moderator analyses revealed significant effects of specific intervention types on certain subgroups and subdomains of strength and balance. No studies were found measuring falls.

**Conclusions:**

Structured PA interventions in middle-aged adults improve strength and balance outcomes related to functional impairments and falls. Strength training increases both strength and balance and can be recommended to prevent age-related functional decline. However, the interpretability of the results is limited due to considerable heterogeneity and the overall low methodological quality of the included studies. Long-term trials are needed to determine the preventive potential of PA on strength, balance and falls. This meta-analysis may inform guidelines for tailored training during middle age to promote healthy aging.

*Prospero registration*: CRD42020218643.

**Supplementary Information:**

The online version contains supplementary material available at 10.1186/s40798-023-00606-3.

## Key Points

- Strength training improves muscle strength and postural balance in middle-aged adults and can be considered a countermeasure to functional decline.
- Studies measuring the effect of PA interventions on falls are lacking in middle-aged adults.

## Background

Functional impairments and falls have been established correctly as global issues of older age but neglected in middle-aged adults [1–4]. However, recent studies highlight the immense public health importance of these two issues in adults aged 40–64, not only because of the significant impact on workforce participation and public expenditures [4] but, more importantly, because of the prevention of adverse events.

In a cohort study of 6874 community-dwelling adults, 22% developed impairments in activities of daily living (ADL) between the ages of 50 and 64 [5]. Although middle-aged people have a higher capacity to recover from an injury than older people [4] and are more likely to have temporary limitations only, half of them had persistent limitations, and 9% developed a further ADL decline within two years [5]. Individuals aged between 50 and 64 years who develop an ADL restriction have a 1.5 to 2.5-fold more significant risk of hospitalization, nursing home admissions and premature death [5, 6]. Falls are among the leading causes of disability-adjusted life-years, outranking other diseases such as asthma, osteoarthritis, chronic kidney disease and dementia [7]. Although much research has been done on fall risk in the last decades, an increasing trend of falls with injuries for adults of all ages has been observed [8], indicating a need for new fall prevention strategies. Previous studies reported fall rates of 11.4–31.4% in middle-aged adults [2, 3, 8, 9], of which 35% are injurious falls [3]. The risk of suffering a fall [2] or an injurious fall requiring medical treatment in the emergency room increases sharply in middle age, especially for women [1]. And since past falls are the strongest predictors of future falls [10] and among the strongest risk factors for injurious falls [11], there is an urgent need for primary prevention strategies.

Lower limb muscle strength and postural balance are modifiable major risk factors predicting mobility restrictions, functional impairments [12–15] and falls [10, 16]. Strength and balance decrease significantly between the age of 40 and 60 [17, 18], but the age-related decline does not appear considerably before 50 [19, 20]. The earlier functional decline can be attributed to an inactive, sedentary lifestyle and regular training can improve performance, even for inactive people [19]. Accordingly, PA interventions in middle-aged adults aimed at long-term maintenance and improvement of lower limb muscle strength and postural balance could prevent loss of function in motor domains essential for mobility, independent living and fall prevention in middle-aged and older adults. Given these facts, it is unclear why prevention programs should start later, once functional decline has already progressed, and a non-negligible proportion of people have already fallen [21, 22]

Indeed, early prevention of functional impairments and falls has been widely neglected so far. Guidelines for fall prevention have focused on older adults aged 65 and older [23, 24], despite current studies calling for prevention starting in middle age [1, 2]. Preventive health care research for middle-aged people has mainly focused on people with diseases such as heart disease, diabetes and stroke [4, 25]. In contrast, no systematic review could be found investigating the effects of balance and functional training on the risk of falls for middle-aged adults [26]. A systematic review by Ferreira et al. [27] revealed moderate effects of PA on muscle strength and postural balance in middle-aged adults. However, they did not conduct subgroup analyses, did not explicitly look for falls in their search strategy and did not differentiate between types of interventions. Therefore, it is time to update and expand upon this previous analysis.

To make a step toward early prevention of falls and functional decline, starting with middle-aged adults, evidence of the effectiveness of different types of PA for preventing falls and relevant physical outcomes is needed. Accordingly, this systematic review and meta-analysis of randomized controlled trials aims to investigate the short- and long-term effects of general PA and structured PA (i.e., specific training such as strength and balance training) on lower limb muscle strength, postural balance and falls in middle-aged adults.

## Methods

This systematic review was conducted following the recommendations of the 'Preferred Reporting Items for Systematic Reviews and Meta-Analyses' (PRISMA) [28]) and registered at PROSPERO (CRD42020218643).

### Literature Search

A computerized systematic literature search was performed within the databases MEDLINE, CINAHL, Web of Science and Cochrane Library for studies published up to November 18, 2022. Relevant search terms were combined with Boolean operators (OR/AND/NOT) (Additional file 1: Search strategy). Reference lists of all included studies and content-related reviews were screened. Two researchers from our reviewer team independently screened titles and abstracts for inclusion using Rayyan software (ww.rayyan.com). Pairs of reviewers independently screened all potentially eligible full texts for inclusion. Disagreement was resolved by discussion with the reviewer team. One reviewer from the reviewer team extracted the study characteristics, and outcomes from the included studies and another author (MA) double-checked the extracted data. Missing data related to study outcomes and eligibility were requested from the study authors.

### Eligibility Criteria for Selecting Studies

Studies were included if they met the following criteria: (P) participants: healthy or with specific risk factors for diseases but no specific pathology or acute medical condition (Additional file 1: Additional information on eligibility criteria); age: mean age between 40 and 60 years; mean age + 1 SD < 65 years and mean age – 1 SD > 35 years. (*I*) intervention: any PA except for pure endurance training. (C) comparator: a passive control group that maintained usual activity level or received no intervention, non-specific supportive intervention, sham exercise or placebo. (O) outcomes: at least one measure of lower limb muscle strength (maximal strength, muscle power, strength–endurance), postural balance (steady-static balance, steady-dynamic balance, proactive balance, reactive balance), falls [29] or injurious falls [30]. (S) study design: individual or cluster randomized controlled trial. Studies were excluded if they (a) combined PA interventions with dietary or ancillary materials that could influence the effect of the intervention (Additional file 1: Additional information on eligibility criteria), (b) included only master athletes in their study population, (c) were not in English or German, or (d) were not available in full length.

### Coding of Studies

Data were extracted from the included articles using a standardized Microsoft Excel 2016 form (Microsoft Corporation, Redmond, US). Those studies were coded for the following variables: age, sex, sample size, physical inactivity, type of intervention and strength/balance/fall outcomes. The interventions were grouped based on the fall prevention classification system of the Prevention of Falls Network Europe (ProFaNE) [29] and a previous Cochrane review on fall risk [31] (Additional file 1: Definition of intervention types) into a) general physical activity, b) balance/functional training, c) strength training and d) three-dimensional (3D) training (e.g., Tai Chi, Qi Gong, dance). For reading convenience, the ProFaNE category gait, balance, coordination, or functional task training was referred to as balance/functional training and strength/resistance training (including power) was referred to as strength training. Further categories were added when a study intervention combined two categories or an intervention could not be assigned to one of the categories above: e) strength–endurance training (strength training + endurance training), f) step aerobic g) strength–aerobic training (strength training + step aerobic), h) water-based training and i) whole-body vibration. Studies that reported results for both women and men separately were treated as two individual studies.

### Domains of Muscle Strength and Postural Balance

Strength outcome measures were categorized into the following domains: maximal strength (e.g., one-repetition-maximum, 1RM), muscle power (e.g., countermovement jump height) and strength–endurance (e.g., the number of squats in a minute). Balance outcome measures were categorized according to Shumway-Cook and Woollacott [32]: static steady-state balance (maintaining a steady position in standing, e.g., center of pressure movement during stance), dynamic steady-state balance (maintaining a steady position during walking, e.g., gait speed during 10-m walking), proactive balance (anticipating a postural disturbance such as standing up from a chair, e.g., Timed-Up-and-Go Test, TUG) and reactive balance (compensating for an unexpected perturbation, e.g., center of mass displacements after a slip). If multiple outcome measures were reported (e.g., multiple maximum strength outcomes: leg press and knee extensor strength; multiple static balance outcomes: static balance measures on stable and foam surfaces) for one domain, the most representative one was included in the analysis based on standardized criteria. The selection of the most relevant outcome measures was made on the following criteria: (a) most relevance to falls prevention, (b) most relevance for activities of daily living, (c) used more frequently in the included studies, (d) favorable in terms of data analysis and (e) more challenging and therefore more likely to be accurate for middle-aged adults. In some cases (f) the choice seemed to make no difference but was standardized to reduce heterogeneity (Additional file 1: Criteria for deciding on the most relevant outcome).

### Categories for Subanalyses

According to the mean age of natural menopause [36, 37] and the beginning of age-related skeletal muscle atrophy around the age of 50 [20], we subdivided the population into a lower (< 50 years of age) and higher aged subgroup (≥ 50 years of age). Study populations were considered inactive if they were not physically active more than once a week or more than 60 min/week for six months prior to the study.

### Assessment of Risk of Bias

The Physiotherapy Evidence Database (PEDro) Scale [38] was used to determine the methodological quality of all studies included. Based on 11 items, the internal validity and the presence of statistically replicable information were assessed. A PEDro score of ≥ 6 was considered a cut-off for high study quality.

### Statistical Analyses

To analyze the research question presented, we conducted random-effects network meta-analyses for the primary outcomes of overall strength and overall balance in an attempt to estimate treatment effects compared to the baseline effect of a combined control group.

**Fig. 1:**
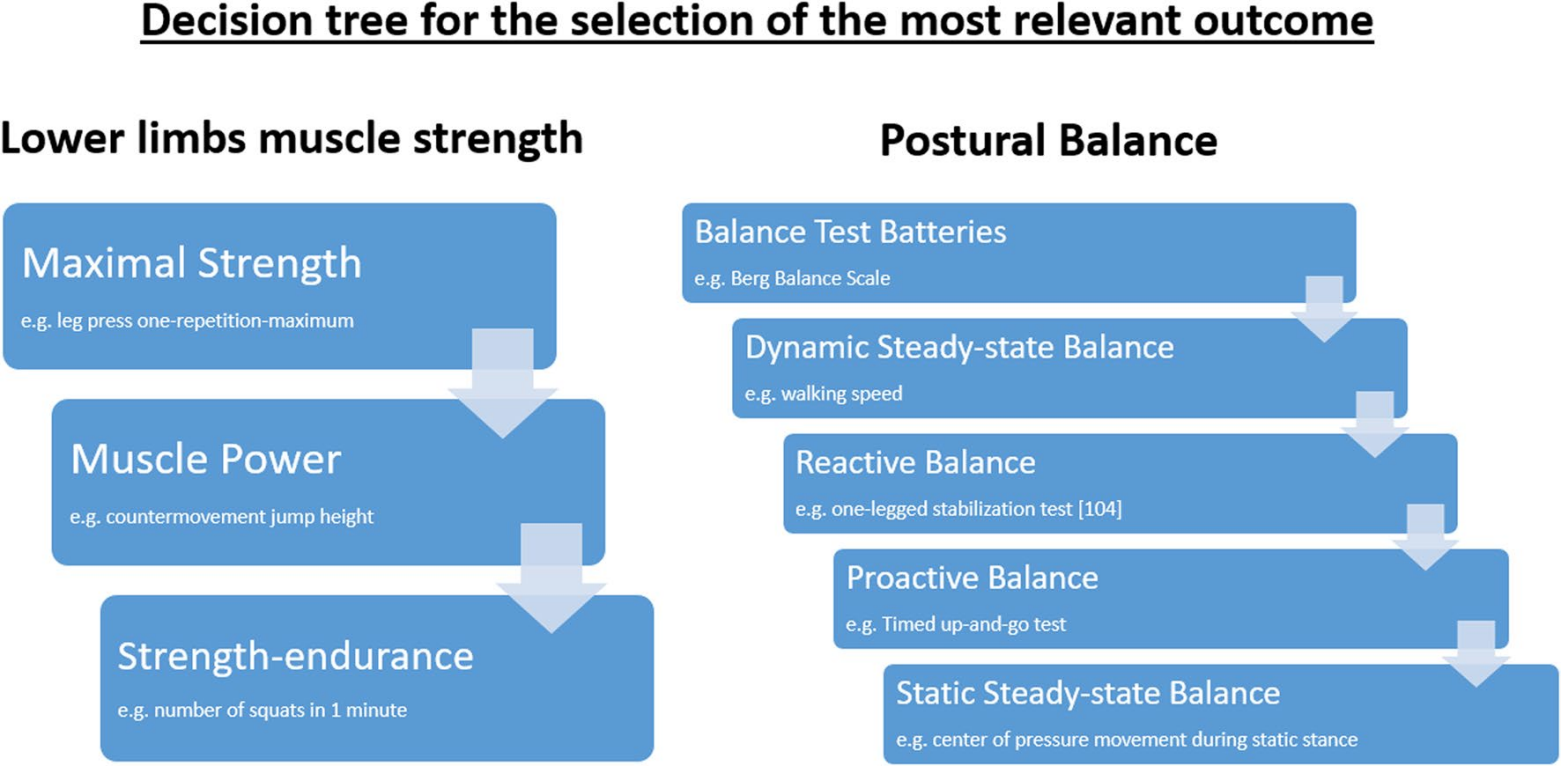
Decision tree for the most relevant outcome of muscle strength and postural balance [35]

To generate overall scores, the most relevant results for muscle strength and postural balance were selected from each study. If multiple outcomes were available in studies, outcomes were ordered with respect to the relevance of our study aims: First, we decided which outcome within each domain of muscle strength and postural balance is most representative based on standardized criteria (see Section “Domains of muscle strength and postural balance” above). Second, we used a decision tree approach from Lacroix et al. [35] to choose the most relevant domain of strength and balance represented in this study (Fig. 1). Based on this procedure, overall scores for balance and strength were generated following previous studies [33, 34].

We employed the method based on graph theory for data synthesis [39] and used *τ*^2^ and *I*^2^ statistics to assess between-trial heterogeneity. Heterogeneity is considered substantial if 75% or above [40]. Treatment effects are expressed as standardized mean differences (SMD) between a treatment and the corresponding control benchmark and reported alongside a 95%-confidence interval. Because of the mixing of interventions present in a large number of studies, we also conducted additive component network meta-analyses [41] in an attempt to isolate the specific treatment effects for every single type of intervention. An SMD of 0.8 or greater was considered a large effect size, between 0.5 and 0.8 was considered a medium effect size, and between 0.2 and 0.5 was considered a small effect size [42].

We created network graphs for all conducted analyses to visualize the connectedness of the related networks for every subgroup of interest and provided tables containing the resulting estimated effects for each network meta-analysis. We stratified the population by sex, age and intervention type for the mentioned subgroup analyses. Besides the primary analysis for overall strength and overall balance, we also analyzed strength and balance in terms of their domains (strength: maximum strength, muscle power, strength–endurance; balance: static, dynamic, proactive and reactive balance). As a sensitivity analysis, we also stratified the study selection by PEDRO score to investigate whether or not the results of our analyses differ for the studies with a high risk of bias and the ones with low risk.

We used forest plots to illustrate the estimation results of the two modeling approaches (conventional network meta-analysis [NMA] versus additive component NMA) against one another to detect discrepancies. We investigated the presence of small study effects using funnel plots for the outcomes of overall strength and balance together with Egger's regression test [43].

All analyses were performed in R [44] (version 4.1.2) using the packages meta [45] and netmeta [46].

## Results

### Study Selection

**Fig. 2:**
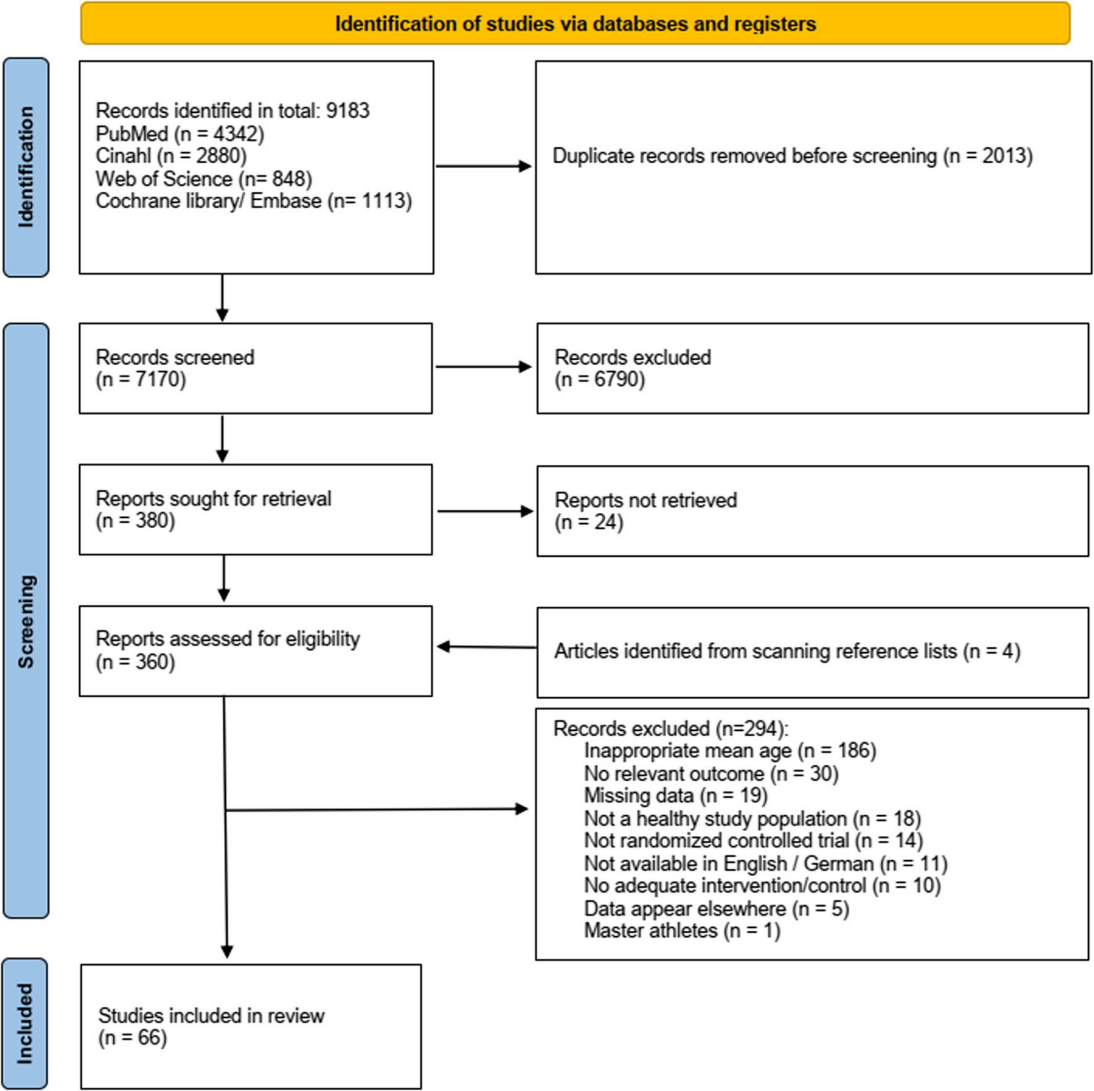
Study flow from the literature search

After removing duplicates, the systematic search strategy resulted in 7170 articles (Fig. 2). Four further articles were identified by screening reference lists [47–50]. The screening of title and abstract led to the inclusion of 360 articles in the full-text screening, from which 294 articles were excluded (Additional file 1: Included and excluded studies in full-text screening). To request relevant data not reported, the authors of 63 studies were contacted; 25 of these provided relevant information. Finally, 66 studies fulfilled the inclusion criteria and were considered in the analysis. Because it is impossible to estimate the absolute effect of an intervention in an NMA, all of the following effect sizes must be regarded compared to the control group.

### Characteristics of Included Studies

This meta-analysis comprises 66 studies (Table 1; Additional file 1: Individual study characteristics) with 88 experimental groups and 3387 participants (*n* experimental = 1929, *n* control = 1458). With one exception [51], all study interventions were structured PA interventions. Fifty-six studies reported at least one outcome for lower limb muscle strength and 31 studies for postural balance. In total, 95 outcome measures of strength (maximal strength: 70; power: 15; strength–endurance: 10) and 53 outcome measures of postural balance (static: 23; proactive: 20; dynamic: 9; reactive: 1) were found. The outcome measures falls or fall injuries were not found in any included study. Three studies reported follow-up data from 8 to 26 weeks after post-assessment [52–54]. Network meta-analysis (NMA) was not possible for these outcomes due to insufficient data. The full results of all analyses are illustrated in Additional file 1: Full results.

**Table 1:** Summary of characteristics of studies included in the analysis

Study characteristics	No. of (%) (*k* = 66)
*Sex*	
Exclusively women	45 (68.2)
Exclusively men	12 (18.2)
Women & men	8 (12.1)
Not reported	1 (1.5)
*Mean age*	
< 50 years	16 (24.2)
≥ 50 years	46 (69.7.)
Age range reported	4 (6.1)
*Activity status*	
Physical Inactive	20 (30.3)
Not reported	46 (69.7)
Intervention types*	
Strength training	41 (46.6)
Strength–endurance training	15 (17.0)
Balance/Functional training	10 (11.4)
Step aerobic training	5 (5.7)
Three-dimensional (3D) training	5 (5.7)
Strength–aerobic training	5 (5.7)
Whole-body vibration (WBV)	3 (3.4)
Water-based training	3 (3.4)
General physical activity	1 (1.1)

*k* Number of included studies

*No. of RCTs* Number of randomized controlled trials.

*Studies examining different types of interventions may be counted multiple times

### Risk of Bias

The median PEDro score of all included studies was 5 (IQR 2). Forty-seven out of 66 (71.1%) studies had a score below 6, indicating a high risk of bias (Additional file 1: PEDro scores of included studies), while 19 (28.8%) studies had a score of 6 and higher, indicating a low risk of bias. The most common reasons for downgrading the study quality were an unclear or lack of concealed allocation (84.9%), lack of blinding of all subjects (100%), all therapists (100%) and all investigators (77.3%), as well as non-use of intention-to-treat methods (78.8%). In contrast, most studies had a low risk of bias for randomization (100%), similar baseline results (91.9%), dropouts (60.6%), statistical between-group comparison (100%) and reporting of point and variability measures (98.5%).

### Effects of PA on Lower Limb Muscle Strength

#### Main Effects of Intervention Types on Overall Strength

**Fig. 3:**
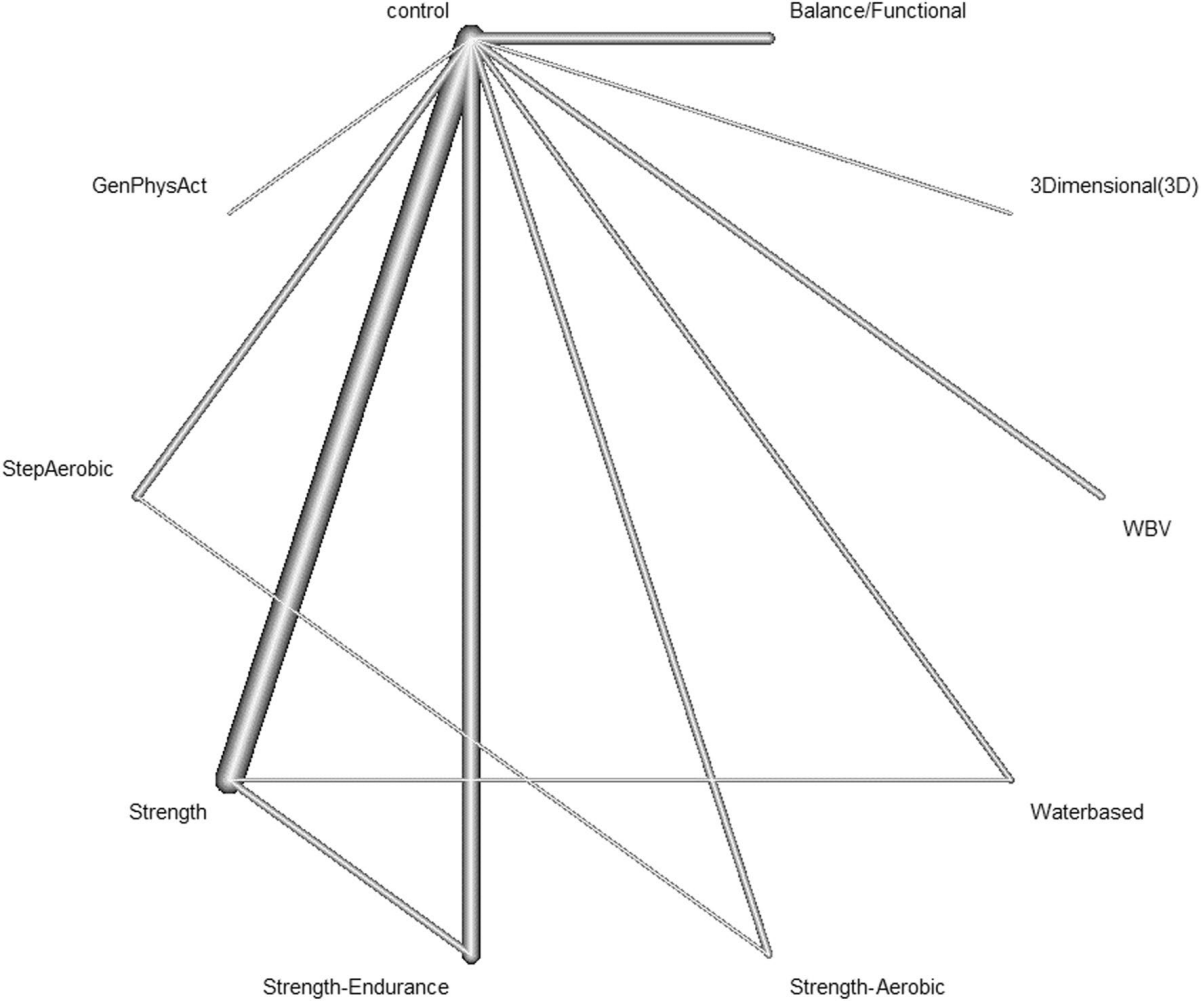
Network plot of all treatment groups in in the meta-analysis for the effects of PA on overall strength. Thicker connecting lines represent a higher number of studies comparing the corresponding interventions. *GenPhysAct* General Physical Activity. *WBV* Whole-Body Vibration

The NMA (Fig. 3) for the primary outcome *overall strength* included 52 studies (*I*^2^ = 79.3%). Compared to the control groups, strength training, strength–aerobic training, strength–endurance training and water-based training showed strong, significant effects on overall strength (Table 2). However, when exclusively studies with a low risk of bias were considered (16 studies, *I*^2^ = 84.5%), only the effects of strength training remained strong and significant. Visual inspection of the funnel plots and Egger's test (*p* = 0.0031) suggested significant asymmetry in our study population, indicating the presence of publication bias (Additional file 1: Funnel plots).

**Table 2:** Effect of intervention types on overall strength

Intervention type	SMD^a^	95% CI	*p*-value
Step aerobic training	0.39	− 0.23; 0.99	0.22
Balance/ Functional training	0.57	− 0.03; 1.18	0.06
Three-dimensional (3D) training	0.79	− 0.70; 2.28	0.30
General PA	− 0.04	− 1.38; 1.30	0.95
Strength training	1.02	0.73; 1.30	< 0.01**
Strength–aerobic training	1.41	0.80; 2.02	< 0.01**
Strength–endurance training	0.92	0.49; 1.36	< 0.01**
Whole-body vibration (WBV)	0.47	− 0.42; 1.36	0.30
Water-based training	1.08	0.06; 2.09	0.04*

^a^Effect sizes of intervention types compared to the control group

*95% CI* 95% confidence interval, *SMD* standardized mean differences, *PA* Physical activity

^*^ significant with p ≤ 0.05 ** highly significant with p ≤ 0.01

### Moderator Analysis of the Effects of PA Interventions on Overall Strength

#### Age

In the lower aged subgroup (13 studies, *I*^2^ = 57.1%), strength training (SMD = 1.04, 95% CI 0.64; 1.43) and strength–endurance training (SMD = 1.08, 95% CI 0.56; 1.59) resulted in strong significant improvements of overall strength.

In contrast, in the higher aged subgroup (39 studies, *I*^2^ = 80.6%) strength training (SMD = 1.18, 95% CI 0.77; 1.47), strength–endurance training (SMD = 0.80, 95% CI 0.22; 1.4), step aerobic training (SMD = 1.33, 95% CI 0.48; 2.19) and water-based training (SMD = 1.11, 95% CI 0.07; 2.15) increased overall strength significantly.

#### Sex

In female subjects (37 studies, *I*^2^ = 83.5%), strength training (SMD = 0.93, 95% CI 0.56; 1.30), strength–aerobic training (SMD = 1.36, 95% CI 0.68; 2.04) and strength–endurance training (SMD = 0.92, 95% CI 0.12; 1.71) improved overall strength significantly.

In male subjects (11 studies, *I*^2^ = 63.7%), only strength training (SMD = 1.34, 95% CI 0.89; 1.79) and strength–endurance training (SMD = 1.01, 95% CI 0.55; 1.47) showed significant effects on overall strength.

#### *High-Risk Groups (Female,* > *50 Years, Physically Inactive)*

In studies with women above the mean age of 50 years (31 studies, *I*^2^ = 83.1%), strength training (SMD = 1.14, 95% CI 0.72;1.56), strength–endurance training (SMD = 0.92, CI 95% 0.12; 1.71), step aerobic training (SMD = 1.34, 95% CI 0.42; 2.27) and water-based training (SMD = 1.12, CI 95% 0.01; 2.23) had significant effects on overall strength.

In physically inactive subjects (17 studies, *I*^2^ = 72.3%), only strength training (SMD = 0.97, 95% CI 0.39; 1.54) and strength–endurance training (SMD = 1.13, 95% CI 0.58; 1.67) improved overall strength significantly.

Moreover, in physically inactive, older middle-aged women (10 studies, *I*^2^ = 79.1%), only strength–endurance training (SMD = 1.06, 95% CI 0.12; 2.00) increased overall strength significantly.

### Effects of PA Interventions on Different Domains of Strength (Maximal Strength, Power, Strength–Endurance)

Maximal strength was examined in 46 studies (*I*^2^ = 80%), indicating significant effects of strength training (SMD = 1.23, 95% CI 0.92; 1.53), strength–aerobic training (SMD = 1.69, 95% CI 0.96; 2.43) and strength–endurance training (SMD = 1.05, 95% CI 0.61; 1.49). Considering only studies with low risk of bias (14 studies; *I*^2^ = 87.6%) strength training (SMD = 1.16; 95% CI 0.54; 1.77) and strength–endurance training (SMD = 0.79; 95% CI 0.03; 1.55) remained significantly effective.

Muscle power was measured by ten studies (*I*^2^ = 0%). Significant effects in this domain were present in balance/functional training (SMD = 0.46, 95% CI 0.12; 0.8). Considering studies with low risk of bias (2 studies, *I*^2^ = NA), again, only balance/functional training increased muscle power significantly (SMD = 0.47, 95% CI 0.02; 0.93). Strength–endurance (6 studies, *I*^2^ = 58.8%) was significantly increased by strength training (SMD = 1.11, 95% CI 0.56; 1.67), step aerobic training (SMD = 1.39, 95% CI 0.26; 2.53) and water-based training (SMD = 1.76, 95% CI 0.63; 2.89).

### Effects of PA on Postural Balance

#### Main effects of Intervention Types on Overall Balance

**Fig. 4:**
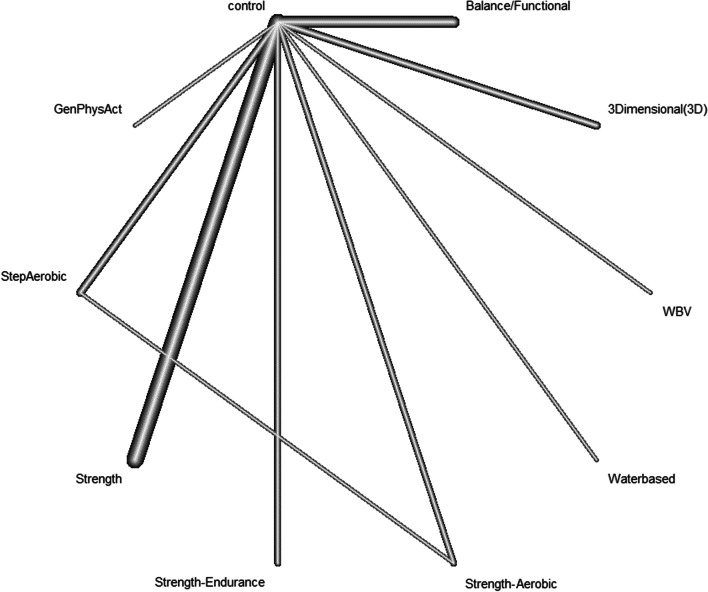
Network plot of all treatment groups in in the meta-analysis for the effects of PA on overall balance. Thicker connecting lines represent a higher number of studies comparing the corresponding interventions. *GenPhysAct* General Physical Activity. *WBV* Whole-Body Vibration

The NMA (Fig. 4) for the primary outcome *overall balance* included 30 studies (*I*^2^ = 88.1%). Compared to the control group, strength training (SMD = 1.16, 95% CI 0.7; 1.62), 3D training (SMD = 1.31, 95% CI 0.25; 2.36) and strength–aerobic (SMD = 0.98, 95% CI 0.12; 1.83) showed strong, significant effects on overall balance (Table 3). Analyzing studies with a low risk of bias only (*n* = 7; *I*^2^ = 60%), only balance/functional training (SMD = 0.48, 95% CI 0.13; 0.84) showed moderate effects with a significant difference from the control group. Visual inspection of the funnel plots and Egger's test (*p* = 0.07) suggested no significant asymmetry in our study population, indicating the absence of publication bias (Additional file 1: Funnel plots).

**Table 3:** Effect of intervention types on overall balance

Intervention	SMD^a^	95% CI	*p*-value
Step aerobic training	0.18	− 1.0; 0.64	0.66
Balance/ Functional training	0.48	− 1.19; 1.16	0.16
Three-dimensional (3D) training	1.31	0.25; 2.36	0.02
General physical activity	− 0.21	− 1.80; 1.37	0.79
Strength training	1.16	0.70; 1.62	< 0.01**
Strength–aerobic training	0.98	0. 12; 1.83	0.02
Strength–endurance training	0.11	− 1.07; 1.30	0.85
Whole-body vibration (WBV)	0.17	− 1.54; 1.88	0.85
Water-based training	− 0.28	− 2.04; 1.48	0.75

^a^Effect sizes of intervention types compared to the control group

*95% CI LB* lower bound of 95% confidence interval, *SMD* standardized mean differences

^*^Significant with *p* ≤ 0.05 ** highly significant with *p* ≤ 0.01

### Moderator Analysis of the Effects of PA Interventions on Overall Balance

#### Age

In the lower aged subgroup (7 studies, *I*^2^ = 86.2%), no intervention type showed significant improvements in overall balance compared to the control group. In contrast, in the higher aged subgroup (23 studies, *I*^2^ = 84.6%), strength training (SMD = 1.69, 95% CI 1.08; 2.29) and 3D training (SMD = 1.30, 95% CI 0.26; 2.33) increased overall balance significantly.

#### Sex

In female subjects (22 studies, *I*^2^ = 85.6%), strength training (SMD = 0.91, 95% CI 0.41; 1.41), strength–aerobic training (SMD = 0.82, 95% CI 0.05; 1.60) and 3D training (SMD = 1.26, 95% CI 0.32; 2.20) improved overall balance significantly. Only one study investigated balance and male subjects solely, and no NMA was therefore performed for this sub-outcome.

#### High-Risk Groups (Female, Older, Physically Inactive)

In women above a mean age of 50 years (17 studies, *I*^2^ = 83.7%), strength training (SMD = 1.19, 95% CI 0.51; 1.87) and 3D training (SMD = 1.27, 95% CI 0.3; 2.23) improved overall balance significantly. Furthermore, in physically inactive subjects (2 studies, *I*^2^ = NA), strength training (SMD = 0.81, 95% CI 0.15; 1.46) and balance/functional training (SMD = 1.27, 95% CI 0.66; 1.89) had significant effects on overall balance. The strength training and balance/functional training were conducted in physically inactive women above the age of 50 years (2 studies, *I*^2^ = NA). Therefore, these results remain for this population.

### Effects of PA Interventions on Different Domains of Balance (Static, Dynamic, Proactive, Reactive)

For both static balance (16 studies, I^2^ = 69.4%) and dynamic balance (SMD = 2.31, 95% CI 1.05; 3.57), only strength training (static balance: SMD = 0.41, 95% CI 0.05; 0.77; dynamic balance: SMD = 2.31, 95% CI 1.05; 3.57) showed significant effects. When considering only studies with low risk of bias (static balance: 4 studies; *I*^2^ = 0%; dynamic balance: 1 study, *I*^2^ = NA) no intervention type showed significant effects on static balance. The effects of PA on proactive balance were examined by 17 studies (*I*^2^ = 71.1%), resulting in significant effects of strength training (SMD = 1.53, 95% CI 1.07–1.99), 3D training (SMD = 1.98, 95% CI 0.97; 2.98) and strength–aerobic training (SMD = 1.74, 95% CI 0.89; 2.58). However, considering studies with a low risk of bias (5 studies, *I*^2^ = 68.5%), only balance/functional training (SMD = 0.55, 95% CI 0.07; 1.03) led to significant effects on proactive balance. Reactive balance was only investigated in one study and no NMA was therefore performed for this sub-outcome.

## Discussion

This systematic review and meta-analysis compares the effects of different types of PA interventions on lower limb muscle strength, postural balance and falls in healthy middle-aged adults. Our main finding is that strength training resulted in strong effects on both muscle strength and postural balance. Based on these findings, strength training is highly beneficial for counteracting functional decline during middle age. Moreover, the network analysis revealed the effects of specific intervention types on specific capacity domains and balance subcomponents in specific subgroups. This information may inform specific guidelines for tailored training during middle age to promote healthy aging. No RCT with outcome measures on falls or fall injuries was found.

### Effects of PA on Lower Limb Muscle Strength

Strong and significant effects on lower limb muscle strength were found when strength training was performed, either alone or combined with step aerobic or endurance exercise. This is in line with the analyses by Ferreira et al. [27], who found greater effects on muscle strength from interventions that included at least components of resistance training. Interestingly, the effect sizes in our analysis were comparable or even greater when strength training was combined with other intervention types. This finding is consistent with a study by Irving et al. [55], in which combined strength and endurance training resulted in more robust improvements in mitochondrial physiology and physical characteristics compared with strength or endurance training alone, despite lower training volumes and independent of age. This is important because mixed interventions have the potential to improve other aspects of health in addition to muscle strength, e.g., by reducing body fat [56]. As well, they are more varied and therefore could be more motivating and appropriate for holistic prevention programs.

Focusing on specific populations, we present evidence for several intervention types that can be recommended: in middle-aged women of at least 50 years, our results suggest that water-based interventions may increase lower limb muscle strength equivalent to strength training. Water-based interventions benefit from the physical properties of water, including buoyancy and hydrostatic pressure, resulting in high training resistances comparable to those of weights. Additionally, water-based interventions have beneficial effects on blood pressure [57, 58] and bone metabolism [59]. Of course, not only the content (water-based interventions or strength training) but also the loading scheme (e.g., training duration, frequency, volume etc.) differed between the studies. Thus, there might be multiple factors responsible for the observed consistency in effect size between water-based training and strength training. In middle-aged adults above 50 years, muscle strength is also increased by step aerobic training. In addition, physically inactive individuals benefit from strength training or strength–endurance training. Accordingly, in specific subgroups, effects on muscle strength can be achieved through different interventions. Given that only a minority of people over the age of 50 engage in regular strength training [60], it is crucial to both promote strength training and offer attractive alternatives to reach a broader population.

Balance/ functional training, 3D training, general PA and whole-body vibration did not significantly affect lower limb muscle strength compared to the control group. This could be because these types of interventions do not correspond to the principles of the training of muscle strength. To adapt structurally and functionally and increase muscle strength, our organisms need overloads and specific, biomechanically relevant stimuli [61]. Accordingly, these interventions cannot be recommended for improving muscle strength in middle-aged adults, based on the current evidence.

### Effects of PA on Different Domains of Strength

When maximum strength was analyzed, strong effects of strength training alone or combined with step aerobic or endurance exercise were evident. Maximum strength is the most prevalent domain of muscle strength in our research and a crucial component for many functional activities such as jumping, running or changing direction [62]. However, recent studies discuss a more dominant role of muscle power in terms of physical function [63, 64] and prevention of falls [65].

Despite the relationship between maximum strength and muscle power [66, 67], muscle power was not significantly improved by strength training in our analysis. Only functional/balance training presented significant effects on muscle power and small effect sizes (SMD = 0.46). This is surprising, as de Resende-Neto et al. [68] found equivalent effects of functional training (multi-functional exercises according to the participants’ daily needs) and machine-based resistance training on muscle power. They concluded that the physiological stress has greater impact on functional performance than the specific type of strength training in older women. However, our results indicate that balance/functional training might be specifically effective to increase muscle power in middle-aged adults. This is consistent with the results of a meta-analysis by Moran et al. demonstrating that jump training, a functional exercise, can improve muscle power in older adults [33]. All in all, the number of studies on muscle power (n = 10) was too small to draw a conclusion on the most effective type of training for this strength domain. Given its importance for functional status [69], there is an urgent need to fill this lack of evidence with high-quality studies.

### Effects of PA on Postural Balance

Based on our analysis, 3D training, strength training and strength training combined with step aerobic can be considered effective in improving postural balance in middle age and can be recommended as a countermeasure to prevent age-related balance decline. Strength training improved balance also in subgroups at increased risk of falls and functional decline (inactive, older, female middle-aged adults). These results are in line with previous meta-analyses showing significant effects of resistance training [72] and 3D training [73] on postural balance in older populations. Our review highlights that these effects are also evident in middle age. Moreover, our findings suggest that people over the age of 50 years reap a greater benefit from these types of training, as compared to those below the age of 50 years. We speculate that older participants have more room for improving balance control via training. On the same note, our findings may be attributed to the fact that we included only 16 studies with a mean age below 50 years. Of these, only seven measured postural balance, again highlighting the lack of evidence in middle-aged adults. Choy et al. [18] showed that deficits in balance begin at age 40 and steadily increase. Therefore, when aiming to prevent balance disorders, this age is a critical time point, and the lack of evidence is problematic.

In our analysis, balance/functional training was effective exclusively in the subgroup analysis of higher aged, inactive women but showed no significant effects on overall balance in the total sample. This is surprising since previous meta-analyses presented effects of balance training on balance performance in youth [74], young adults [75] and older adults [76]. There could be several reasons our results do not reflect this: (1) We found only seven studies conducting balance/functional training and measuring postural balance. (2) Balance tests applied in clinical studies are very heterogeneous. To obtain an overall balance score, we included only the most relevant of each study for functional capacity [35]. Therefore, significant results may not have been considered in the primary analysis. (3) The grouping of functional and balance training according to ProFaNE might have blurred the results: a standardized categorization of interventions is required when conducting a meta-analysis. Functional and balance training are difficult to separate as most functional activities also require balance skills. However, primarily functional training such as stair climbing [77] or jumping exercises [48] seems more likely to lead to significant changes in muscle strength, while primary balance training such as single-leg stance is more likely to improve specific skills of postural balance. At the same time, postural balance training is highly task-specific [78–80]. Accordingly, grouping functional and balance training into a single category could lead to an underestimation of their respective effects on strength and balance. Instead, a differentiated consideration of postural balance training according to the subdomains of postural balance [32] might be more appropriate. All in all, these facts might have blurred the analysis and led to an underestimation of balance/functional training to improve postural balance.

Water-based exercise is recommended for older adults with balance impairment [81] and appears to be effective in improving balance in older adults [82] and individuals with neurological diseases [83, 84]. Our analysis discovered no significant effects on postural balance in middle-aged adults. However, given the revealed impact on muscle strength, water-based exercises are an interesting intervention for preventing functional decline and falls; further studies are needed to explore their actual potential. Strength–endurance training was also not effective in improving postural balance. Two strength–endurance training studies [85, 86] were included in the analysis of postural balance. While Park et al. [85] found significant effects on backward tandem walking time over six meters in postmenopausal women, Chilibeck et al. [86] found no effects on one-legged standing time with eyes closed in postmenopausal obese women. Accordingly, current evidence does not support a conclusion on the impact of strength–endurance training on postural balance, mainly since these two studies used very different outcome measures for different balance domains. Considering the positive effects of endurance training on motor learning [87], further studies should investigate the effectiveness of this mixed intervention type on balance and other fall risk factors. In line with previous meta-analyses in non-frail populations, whole-body vibration did not significantly affect postural balance in middle-aged adults [88, 89].

### Effects of PA on Different Domains of Postural Balance

To the best of our knowledge, this is the first systematic review and meta-analysis examining the effects of PA on subdomains of postural balance in middle-aged adults. Studies by Muehlbauer et al. [90–92] and other authors [78–80] emphasize postural balance to be highly task-specific. Thus, we also analyzed the included studies regarding the different domains of balance defined by Shumway-Cook and Woollacott [32]. In line with previous reviews on older adults [76, 89], static balance was the most widely tested balance domain in our review on middle-aged adults.

Previous studies and meta-analyses found significant effects of several physical intervention types on different postural balance domains in young and older adults [74–76, 89, 93, 94]. Therefore, it is surprising that only strength training showed significant effects on static and dynamic balance but no other intervention type (such as balance/functional training, step aerobic etc.). One reason for this may be that some studies [48, 53, 95, 96] used balance measures that may not be sensitive for healthy middle-aged adults, such as measures of the center of pressure displacements during static bipedal stance [18, 97] or habitual gait speed [98]. The application of center of pressure measures during single-legged stance with eyes closed, bipedal stance on a foam surface [18] and maximum gait speed appear to be more valid measures [98]. Despite the relatively high number of 23 data points for static balance and nine data points for dynamic balance, current evidence supports only strength training to improve static and dynamic balance capacity, highlighting the importance of strength training in middle-aged adults.

Proactive balance was the second most frequently assessed balance domain (20 data points; e.g., timed up and go). Previous studies have demonstrated effects on this domain by strength training [99] and balance training [76] in older adults. Consistent with these findings, our results indicate that strength training, strength–aerobic training and 3D training [100] are effective interventions in middle-aged adults. Both step aerobic exercise and 3D training involve a high extent of movement planning, anticipation of postural disturbance and efficient transfer of bodyweight from one part of the body to another. These specific abilities are closely related to proactive balance [32]. Based on this, we recommend 3D training, strength training, or strength training combined with step aerobic training to improve proactive balance. However, further high-quality studies are needed to confirm this.

Reactive balance, which is closely related to falls [101] and probably the most critical balance domain for fall prevention, was only measured in one study [102]. Based on this, no conclusion can be drawn on the effects of PA interventions on reactive balance in middle-aged adults. This is not surprising as reactive balance is severely underrepresented in the clinic [103], and there has been relatively little research on reactive balance testing [101]. We included only one study by Deibert et al. [102], who found significant effects of strength training on postural balance following unannounced mediolateral perturbations in subjects standing on a posturomed platform [104]. Emerging technologies [105, 106] offer measures that are probably more valid to assess reactive balance and likely to be adaptable to middle-aged adults. Future studies should address this task.

### Effects of PA on Falls

Despite an extensive search for this review, no study investigating the effects of PA on falls or injury-related falls in middle-aged adults was found. Ferreira et al. [27] identified one study measuring falls [107] that was not included in our analysis because it was an endurance intervention only. Pereira et al. [107] conducted an eight-week walking program that showed no significant effect on fall rates at the ten-year follow-up. In summary, there is a significant lack of evidence, which is highly concerning given the immense impact of falls on middle-aged people.

### Follow-up Measurements

An insufficient number of studies examined the maintenance of training effects after the interventions were completed. Individual studies indicate that training effects on lower limb maximum strength remain significantly improved for at least 8 [53, 54] to 26 weeks [52], while muscle power does not. Since muscle power may be of particular importance for tasks of daily living, such as climbing stairs [69], interventions are needed to achieve lasting effects on this strength domain. Also, improvements in proactive balance can last 26 weeks after completing the intervention [54]. Dynamic and static balance can even improve after eight weeks [53]. However, only three studies examined muscle strength and postural balance maintenance. Hence, results must be interpreted with caution. Further studies are needed to confirm these results so that efficient exercise interventions with long-term effects on lower limb muscle strength and postural balance can be planned.

### Study Quality and Populations

The methodological quality of most of the included studies (71.2%) was low. However, some quality aspects, such as blinding the participating subjects, are often difficult or impossible to ensure when conducting PA studies. In addition, many studies in this review also lacked other aspects, such as concealed allocation, sound statistical analysis and reporting according to established standards, which urgently need to be addressed in future studies.

At 68.2%, the proportion of studies that included women exclusively was high, whereas only 18.2% of studies included men exclusively. These rates are comparable to those of Ferreira et al. (2012) [27], where 69.5% of studies included women only and 9.5% included men only. Also, the number of studies examining subjects with an average age of at least 50 years (69.7%) was considerably higher than those with younger subjects. This imbalance of sex and age could be because menopause is known to have a major impact on quality of life, metabolism and risk of chronic conditions [108]. Regarding the consequences of menopause, PA is recognized as an essential prevention tool [108]. In contrast, the impact of functional impairments and falls in middle-aged men and women [3], their long-term effects [6] and the potential of early prevention have been neglected.

### Heterogeneity

Heterogeneity in our primary analyses ranged from 79.3 to 88.1% and is therefore considered substantial. In our subanalyses, heterogeneity varied widely from 0 to 88.1%. Some subanalyses showed only low to moderate heterogeneity. Neither the performance of subanalyses nor the search for structural similarities of outliers provided explanations for the overall heterogeneous results. Therefore, we consider that this is likely due to differences between studies in specific characteristics of their interventions (e.g., period, intensity) and target groups, which warrants further evaluation. Since we could not find structural reasons for the considerable heterogeneity, we decided to conduct the meta-analysis, taking into account heterogeneity (*I*^2^ ≥ 75%). However, this high heterogeneity is consistent with earlier meta-analyses of PA interventions [76, 109] and seems to be a general problem in this field of research.

### Limitations of the Review

Despite our thorough search process, we are aware that some relevant studies may not have been included, especially when not published in English or German. To standardize the classification of interventions, we referred to an established paradigm for fall prevention [31, 110]. We also formed new categories to reduce subjectivity when more than one exercise category was met [31] and when the new category seemed to make the analysis more precise. Nevertheless, there remains a certain subjectivity within the classification. The number of studies we found for step aerobic training, 3D training, strength–aerobic training, whole-body vibration, water-based training and general physical activity was tiny. Final conclusions on the effectiveness of these intervention types require further investigations.

### Recommendations for the Practice of Early Prevention of Functional Decline and Falls in Middle-Aged Adults

Based on our meta-analysis, we provide the following PA recommendations:

#### Lower limb muscle strength:

- Strength training, strength–aerobic training and strength–endurance training increase lower limb muscle strength.
- Step aerobic, whole-body vibration, 3D training and general PA cannot be recommended to improve muscle strength
- In middle-aged women over 50 years of age, water-based training may be an equivalent alternative to strength training for improving lower limb strength
- In physically inactive populations, strength training and balance/functional training can be applied to increase lower limb muscle strength
- Balance/functional exercises are beneficial for improving muscle power

#### Postural Balance

- 3D training, and strength training either alone or combined with step aerobic, can be recommended to improve postural balance
- Strength–endurance training, whole-body vibration and general PA cannot be recommended to improve postural balance
- Balance/functional exercises can improve balance in inactive women over the age of 50 years

### Implications for Future Research

Based on our systematic review, we provide the following recommendations for future research in middle-aged adults:

- There is an urgent need to explore the short- and long-term effectiveness of PA interventions on falls and injurious falls
- Further studies are needed on the effects of different PA interventions on lower limb muscle strength and postural balance and their short- and long-term value
- Future studies need to pay more attention to adhering to quality standards to obtain high-quality data.
- Little is known about the validity of postural balance assessments in middle-aged adults. Validation studies and uniform balance test sets are needed to enable meta-analyses
- Rather than considering balance/functional training as a single category, differentiated grouping interventions based on postural balance domains [32] might increase the informative value of future studies.

## Conclusion

Strength training improves muscle strength and postural balance in middle-aged adults and can be considered a central pillar for preventing the functional decline in this age group. Different intervention types show effects in specific subdomains and subpopulations and can also be recommended. These findings are essential to address the severe loss of lower limb muscle strength in middle age, a period of particular importance for early prevention of falls and loss of function in broad populations worldwide. Future guidelines should consider the enormous potential of targeted physical activity programs for early prevention of functional decline and falls. In addition, there is a need for more high-quality studies to investigate the effects of different types of PA intervention on strength, balance and falls in middle-aged adults. This work presents the basis for developing a new paradigm of early prevention, which could lead to a significant reduction of functional decline and fall rates, and two critical problems in health systems worldwide.

## Supplementary Information

**Additional file 1.** Additional information on eligibility criteria; Definition of intervention types; Criteria for deciding on the most relevant outcome; Included and excluded studies in full-text screening; Individual study characteristics; Full results; PEDro scores of included studies; Funnel plots.

## Data Availability

The data extracted from the included studies, the analytic code and other materials used in the review are available from the corresponding author on reasonable request. A review protocol was not prepared.

## Abbreviations

PA: Physical activity
PEDro: Physiotherapy Evidence Database
PRISMA: Preferred Reporting Items for Systematic Reviews and Meta-Analyses
SMD: Standardized mean differences
3D training: Three-dimensional training
95% CI: 95% Confidence interval
