# Supplementary material for: Effects of Physical Activity Interventions on Strength, Balance and Falls in Middle-Aged Adults: A Systematic Review and Meta-Analysis
Source: Sports Med Open. 2023 Jul 19;9:61. doi: 10.1186/s40798-023-00606-3 (PMC10356733; doi:10.1186/s40798-023-00606-3)
Supplement: Supplementary file 1 — Additional file 1. Additional information on eligibility criteria; Definition of intervention types; Criteria for deciding on the most relevant outcome; Included and excluded studies in full-text screening; Individual study characteristics; Full results; PEDro scores of included studies; Funnel plots. [file 40798_2023_606_MOESM1_ESM.docx]

**Electronic Supplementary Material**

**Article title:** EArly - Effect of physical activity on strength, balance and falls in middle-aged adults: a systematic review and meta-analysis

**Journal name**: Sports Medicine - Open

Michael Adams^1, 2^, Katharina Gordt-Oesterwind^1,2,3^, Martin Bongartz^4^, Samuel Zimmermann^5^, Svenja Seide^5^ Volker Braun^6^, Michael Schwenk^1,2,7^

1 Network Aging Research, Heidelberg University, 69115 Heidelberg, Germany

2 Institute of Sports and Sports Sciences, Heidelberg University, 69117 Heidelberg, Germany

3 Unit Digitale Geriatrie, Geriatric Center of Heidelberg University Hospital, Medical Faculty of Heidelberg University, 69120 Heidelberg, Germany

4 Department of Geriatric Research, Agaplesion Bethanien Hospital, Geriatric Center at Heidelberg University, [69126 Heidelberg](https://www.bing.com/local?lid=YN6740x1996224626631563108&id=YN6740x1996224626631563108&q=AGAPLESION+BETHANIEN+KRANKENHAUS+HEIDELBERG&name=AGAPLESION+BETHANIEN+KRANKENHAUS+HEIDELBERG&cp=49.39146423339844%7e8.691166877746582&ppois=49.39146423339844_8.691166877746582_AGAPLESION+BETHANIEN+KRANKENHAUS+HEIDELBERG), Germany

5 Institute of Medical Biometry, Heidelberg University Hospital, 69120 Heidelberg, Germany

6 Medical Faculty Mannheim, Heidelberg University, 68167 Mannheim, Germany

7 Human Performance Research Centre, Department of Sport Science, University of Konstanz, 78464 Konstanz, Germany

**Corresponding author:**

Michael Adams (michael.adams@issw.uni-heidelberg.de

**Supplement 1: Search strategy**

**PubMed / Ovid Medline**

| (  "motor activity"[mh] OR  "sports"[mh] OR  "dancing"[mh] OR  "exercise therapy"[mh] OR  "Exercise Movement Techniques"[mh] OR  "Video Games"[Mesh] **OR**  training[tiab] OR  sports[tiab] OR  dance*[tiab] OR  walk*[tiab] OR  gymnastic*[tiab] OR  Exergam*[tiab] OR  "Video Gam*"[tiab] OR  Video-based[tiab] OR  Kinect[tiab] OR  Wii[tiab] OR  "Sony EyeToy"[tiab] OR  IREX[tiab] OR  "Dance Dance Revolution"[tiab] OR  **(**physical*[tiab] **AND** (fit[tiab] OR fitness[tiab] OR train*[tiab] OR exercis*[tiab] OR activ*[tiab])**)**  )  AND  (  "Muscle Strength"[mh] OR  "postural balance"[mh] OR  "posture"[mh:noexp] OR  gait[mh] OR  "Proprioception"[mh:noexp] OR  "strength*"[tiab] OR  Balance*[tiab] OR  postur*[tiab] OR  gait[tiab] OR  propriocepti*[tiab] OR  sensorimotor[tiab] OR  neuromuscular[tiab] OR  "Accidental Falls"[mh] OR  fall[tiab] OR  falls[tiab] OR  falling[tiab] OR  faller*[tiab]  ) |
| --- |
| AND  (  "Controlled Clinical Trial"[pt] OR  "controlled clinical trials as topic"[mh:noexp] OR  **"clinical trial*"[tiab]** OR  random*[tiab] OR  placebo[tiab] OR  **trial[ti]**  )  AND  (  "Middle Aged"[mh] OR  "Middle Age*"[tiab] OR  Midlife[tiab] OR  "40 to"[tiab] OR  "45 to"[tiab] OR  "50 to"[tiab] OR  "40 year*"[tiab] OR  "45 year*"[tiab] OR  "50 year*"[tiab] OR  "40 59 year*"[tiab] OR  "40 60 year*"[tiab] OR  "40 64 year*"[tiab] OR  "40 65 year*"[tiab] OR  "45 59 year*"[tiab] OR  "45 60 year*"[tiab] OR  "45 64 year*"[tiab] OR  "45 65 year*"[tiab] OR  "50 59 year*"[tiab] OR  "50 60 year*"[tiab] OR  "50 64 year*"[tiab] OR  "50 65 year*"[tiab] OR  "aged 40"[tiab] OR  "aged 45"[tiab] OR  "aged 50"[tiab]  )  NOT  ((infant[mh] OR child[mh] OR adolescent[mh] OR "young adult"[mh] OR aged[mh]) NOT "middle aged"[mh])  NOT  (  patient*[tw] OR  hospital*[tw]  )  AND 1900:2020/10/23[edat] |

**COCHRANE LIBRARY**

| ( training OR sports OR dance* OR walk* OR gymnastic* OR Exergam* OR Video **NEXT** Gam* OR Video-based OR Kinect OR Wii OR "Sony EyeToy" OR IREX OR "Dance Dance Revolution" OR **(**physical* **AND** (fit OR fitness OR train* OR exercis* OR activ*)**)** ):ti,ab,kw AND |
| --- |
| ( "strength*" OR Balance* OR postur* OR gait OR propriocepti* OR sensorimotor OR neuromuscular OR fall OR falls OR falling OR faller* ):ti,ab,kw AND |
| ( Middle **NEXT** Age* OR Midlife OR "40 to" OR "45 to" OR "50 to" OR 40 NEXT year* OR 45 NEXT year* OR 50 NEXT year* OR 40 NEXT 59 NEXT year* OR 40 NEXT 60 NEXT year* OR 40 NEXT 64 NEXT year* OR 40 NEXT 65 NEXT year* OR 45 NEXT 59 NEXT year* OR 45 NEXT 60 NEXT year* OR 45 NEXT 64 NEXT year* OR 45 NEXT 65 NEXT year* OR 50 NEXT 59 NEXT year* OR 50 NEXT 60 NEXT year* OR 50 NEXT 64 NEXT year* OR 50 NEXT 65 NEXT year* OR "aged 40" OR "aged 45" OR "aged 50" ):ti,ab,kw |
| NOT ( patient* OR hospital* ):ti,ab,kw AND (**embase**:an NOT pubmed:an) |

**CINAHL**

| ((MH "Dancing+") OR (MH "Physical Fitness+") OR (MH "Sports+") OR (MH "Motor Activity+") OR (MH "Exercise+") OR (MH "Therapeutic Exercise+") OR (MH "Video Games+") OR  "training" OR "sports" OR dance* OR walk* OR gymnastic* OR Exergam* OR "Video Gam*" OR "Video-based" OR "Kinect" OR "Wii" OR "Sony EyeToy" OR "IREX" OR "Dance Dance Revolution" OR **(**physical* **AND** ("fit" OR "fitness" OR train* OR exercis* OR activ*)**)** ) AND |
| --- |
| ((MH "Muscle Strength+") OR (MH "Balance, Postural") OR (MH "Posture") OR (MH "Gait+") OR (MH "Proprioception") OR (MH "Accidental Falls") OR  "strength*" OR Balance* OR postur* OR "gait" OR propriocepti* OR "sensorimotor" OR "neuromuscular" OR "fall" OR "falls" OR "falling" OR faller* ) AND |
| ((MH "Clinical Trials+") OR  "clinical trial*" OR random* OR "placebo" OR (**TI** "trial"))  AND |
| ((MH "Middle Age") OR  "Middle Age*" OR "Midlife" OR "40 to" OR "45 to" OR "50 to" OR "40 year*" OR "45 year*" OR "50 year*" OR "40 59 year*" OR "40 60 year*" OR "40 64 year*" OR "40 65 year*" OR "45 59 year*" OR "45 60 year*" OR "45 64 year*" OR "45 65 year*" OR "50 59 year*" OR "50 60 year*" OR "50 64 year*" OR "50 65 year*" OR "aged 40" OR "aged 45" OR "aged 50" ) |
| NOT ( patient* OR hospital* ) |

**WEB OF SCIENCE**

| TS=( "training" OR "sports" OR dance* OR walk* OR gymnastic* OR Exergam* OR "Video Gam*" OR "Video-based" OR "Kinect" OR "Wii" OR "Sony EyeToy" OR "IREX" OR "Dance Dance Revolution" OR **(**physical* **AND** ("fit" OR "fitness" OR train* OR exercis* OR activ*)**)** ) AND |
| --- |
| TS=( "strength*" OR Balance* OR postur* OR "gait" OR propriocepti* OR "sensorimotor" OR "neuromuscular" OR "fall" OR "falls" OR "falling" OR faller* ) AND |
| (  TS="clinical trial*" OR TS=random* OR TS="placebo" OR **TI=**"trial"  )  AND |
| TS=( "Middle Age*" OR "Midlife" OR "40 to" OR "45 to" OR "50 to" OR "40 year*" OR "45 year*" OR "50 year*" OR "40 59 year*" OR "40 60 year*" OR "40 64 year*" OR "40 65 year*" OR "45 59 year*" OR "45 60 year*" OR "45 64 year*" OR "45 65 year*" OR "50 59 year*" OR "50 60 year*" OR "50 64 year*" OR "50 65 year*" OR "aged 40" OR "aged 45" OR "aged 50" ) |
| NOT TS=( patient* OR hospital* ) |

**Supplement 2: Additional information on eligibility criteria**

**2.1 Health condition: Diseases and risk factors for disease**

*List of diseases excluded at screening and risk factors for diseases accepted in our analysis.*

| **Diseases (Exclusion)** | **Risk factors (Inclusion)** |
| --- | --- |
| Any acute illness, acute pain or acute medical condition.  Neurological Diseases   - Parkinson, Multiple Sclerosis - Stroke - Amyothrophic lateral sclerosis - Cerebral palsy - Fibromyalgia   Ortopaedic Diseases   - Osteoarthritis - Herniated disc - Skoliosis - Impingement syndrome - Chronic pain   Post-surgical rehabilitation   - Fractures - Endoprosthesis, Arthroplasty - Amputees   Pulmonary Diseases   - COPD - Asthma - Pneumonia   Cardiovascular Diseases   - Coronary Heart Disease - Peripheral Artery Disease   Metabolic Diseases   - Diabetes Mellitus Type I + II - Osteoporosis - Cancer (also cancer survivors)   Psychiatric Diseases/ Cognitive Impairments   - Depression - Dementia - Bipolarity - Anorexia, Bulimia nervosa | Metabolic risk factors   - Metabolic syndromeInsulin resistance - Hypertonia - Overweigth, Obesity - Dyslipidemia - Pre-Diabetes - Osteopenia |

**2.2. Nutrition: Accepted and non-accepted supplements**

| **Non-accepted supplements (Exclusion)** | **Accepted supplements (Inclusion)** |
| --- | --- |
| Soy protein [1]  Kreatinase [2] | Calcium  Vitamins |

*Non-accepted supplements influence the effect of the physical activity intervention on our outcomes of interest (muscle strength, postural balance, falls). Accepted supplements are suggested not to influence these outcomes.*

References

Chilibeck, Philip D.; Kaviani, Mojtaba; Candow, Darren G.; Zello, Gordon A. (2017): Effect of creatine supplementation during resistance training on lean tissue mass and muscular strength in older adults: a meta-analysis. In: *Open access journal of sports medicine* 8, S. 213–226. DOI: 10.2147/OAJSM.S123529.

Orsatti, Fábio L.; Maestá, Nailza; Oliveira, Erick P. de; Nahas Neto, Jorge; Burini, Roberto C.; Nunes, Paulo R. P. et al. (2018): Adding Soy Protein to Milk Enhances the Effect of Resistance Training on Muscle Strength in Postmenopausal Women. In: *Journal of Dietary Supplements* 15 (2), S. 140–152. DOI: 10.1080/19390211.2017.1330794.

**Supplement 3: Definition of Intervention Types**

|  | **Exercise category** | **ProFaNE description [3]** | **Sherrington et al. 2020 [4]** | **Definition in this review** |
| --- | --- | --- | --- | --- |
| 1 | Strength training | „The term „Resistance Training“ covers all types of weight training i.e. contracting the muscles against a resistance to ‘overload’ and bring about a training effect in the muscular system. The resistance is an external force, which can be ones own body placed in an unusual relationship to gravity (e.g. prone back extension) or an external resistance (e.g. free weight). All forms of strength training should be based on an *assessment* of the participant’s abilities prior to starting the program; *tailoring* the intervention to the individual‘s abilities and *progression* of the exercise program as ability improves.“ | „Selected as exercise category if the intervention met the baseline assessment, tailoring and progression criteria. Selected as primary category for interventions where additional resistance was used or where it was clear that overload was sufficient without external resistance and where the intervention focus and most time spent was on exercise in this category.“ | No additions were made. |
| 2 | Balance/ Functional training | *„Gait training* involves specific correction of walking technique (e.g., posture, stride length and cadence) and changes of pace, level and direction. *Balance* *Training* involves the efficient transfer of bodyweight from one part of the body to another or challenges specific aspects of the balance systems (e.g.vestibular systems). Balance retraining activities range from the reeducation of basic functional movement patterns to a wide variety of dynamic activities that target more sophisticated aspects of balance. *Functional training* utilises functional activities as the training stimulus, and is based on the theoretical concept of task specificity. All gait, balance and functional training should be based on an *assessment* of the participant’s abilities prior to starting the program; *tailoring* of the intervention to the individuals abilities; and *progression* of the exercise program as ability improves.“ | „Selected as exercise category if the intervention met the baseline assessment, tailoring and progression criteria. Selected as primary category for interventions where most exercises were conducted standing and where the intervention focus and most time spent was on exercise in this category.“ | No additions were made. |
| 3 | 3-Dimensional (3D) training | „3D training involves constant movement in a controlled, fluid, repetitive way through all 3 spatial planes or dimensions (forward and back, side to side, and up and down). Tai Chi and Qi Gong incorporate specific weight transferences and require upright posture and subtle changes of head position and gaze direction. Dance involves a wide range of dynamic movement qualities, speeds and patterns.“ | „Selected as exercise category if the intervention involved Tai Chi or dance. Selected as primary category for interventions where the intervention focus and most time spent was on exercise in this category.“ | Selected as exercise category if the intervention involved Tai Chi, Yoga or dance, but not aerobic (please compare category 7). Selected as category for interventions where the intervention focus and most time spent was on exercise in this category. |
| 4 | General Physical Activity | *„Physical Activity* is any bodily movement produced by skeletal muscle contraction resulting in a substantial increase in energy expenditure. Physical activity has both | „Selected as exercise category if the intervention included unstructured physical activity. We classed programmes that included unstructured walking as this category. Selected as primary | No additions were made. |
|  |  | an occupational, transporational and recreational  components and includes pursuits like golf, tennis, and swimming. It also includes other active pastimes like gardening, cutting wood, and carpentry. Physical activity can provide progressive health benefits and is a catalyst for improving health attitudes, health habits, and lifestyle. Increasing habitual physical activity should be with specific recommendations as to duration, frequency and intensity if a physical or mental health improvement is indicated.“ | category for interventions where the intervention focus and most time spent was on exercise in this category.“ |  |
| 5 | Strength-endurance training  = Strength training (to increase strength, hypertrophy and power) combined with aerobic training (to improve endurance) in a single programme  [5]. | NA | NA | Selected as exercise category if exercises during the intervention combined strength training (as defined above in category 1) and endurance training (as defined in the ProFaNE taxonomy and by Sherrington et al. or if the focus of the intervention and most of the time was spent on exercises in this category.  *ProFaNE:*  *Endurance training* is aimed at cardiovascular conditioning and is aerobic in nature and simultaneously increases the heart rate and the return of blood to the heart.  Sherrington et al. [4]:  Selected as exercise category if the intervention focused on structured aerobic training. We classed programmes that included treadmill walking as this category. Selected as category for interventions where the intervention focus and most time spent was on exercise in this category. |
| 6 | Whole Body Vibration | NA | NA | Selected as exercise category if participants were standing or exercising on a vibration platform during the intervention or if the focus of the intervention and most of the time was spent on exercises in this category. |
| 7 | Step Aerobic | NA | NA | Selected as an exercise category if the exercises during the intervention consisted of dynamic, rhythmic, repetetive movements with dance elements and had the aim of increasing fitness, or if the focus of the intervention and most of the time was on exercises in this category. |
| 8 | Strength-Aerobic exercise | NA | NA | Selected as exercise category if the intervention combined strength training (as defined above in category 1) and step aerobic (as defined above in category 7) or if the focus of the intervention and most of the time is spent on exercises in this category. |
| 9 | Waterbased exercise | NA | NA | Selected as exercise category if the exercises were excecuted in a pool environment or if the focus of the intervention and most of the time is spent on exercises in this category. |

NA – not applicable.

References

1. Lamb SE, Becker C, Gillespie LD, Smith JL, Finnegan S, Potter R, Pfeiffer K. Reporting of complex interventions in clinical trials: development of a taxonomy to classify and describe fall-prevention interventions. Trials. 2011;12:125. doi:10.1186/1745-6215-12-125.

2. Sherrington C, Fairhall N, Wallbank G, Tiedemann A, Michaleff ZA, Howard K, et al. Exercise for preventing falls in older people living in the community: an abridged Cochrane systematic review. Br J Sports Med. 2020;54:885–91. doi:10.1136/bjsports-2019-101512.

3. Wilson JM, Marin PJ, Rhea MR, Wilson SMC, Loenneke JP, Anderson JC. Concurrent training: a meta-analysis examining interference of aerobic and resistance exercises. Journal of Strength and Conditioning Research. 2012;26:2293–307. doi:10.1519/JSC.0b013e31823a3e2d.

**Supplement 4: Criteria for deciding on the most relevant outcome**

| **Multiple measurements** | **Reasoning*** |
| --- | --- |
| **Global strength (e.g. leg press)** vs.  specific muscle groups (e.g. knee extension) | (a, b) Activities of daily living and reactive fall strategies require limb movements through contractions of muscle chains rather than isolated muscle groups. |
| **Extensor** vs. flexor muscle strength | (a) Weak extensor muscles such as the m. quadriceps femoris are particularly strongly associated with the risk of falls in the elderly [6, 7]  (c) In most of the included studies, the muscle strength of the extensor muscles was measured. |
| Lower limb strength measurements:  Unilateral vs. **bilateral** | (c) In most of the included studies, the muscle strength was measured bilaterally. |
| Single leg stance: **Right leg** vs. left leg | (f) Balance performance is not influenced by the use of the dominant or non-dominant leg [8]. Accordingly, it can be assumed that the choice between the left and the right leg is equivalent. |
| Stance with eyes open vs. **eyes closed** | (e) Maintaining balance with eyes closed is more difficult than with eyes open, and postural instability is more likely to be detected in middle-aged adults [9]. |
| Stance on hard surface vs. **soft surface** | (b) Maintaining balance on soft surfaces is more difficult than on hard surfaces, and postural instability is more likely to be detected in middle-aged adults [9]. |
| **Mediolateral** vs. anteroposterio displacement | (a) Mediolateral balance disorders are stronger associated with the risk of falls in the elderly [10–12]. |
| Average gait speed vs. **maximal gait speed** | (e) Normative data suggest that maximum gait speed is reduced in middle-aged adults compared with younger generations, but the average gait speed of women and men in their 40s and 50s is not lower compared with those in their 20s [13]. |
| **Concentric** vs. isometric strength | (a) Reactive fall prevention requires rapid stepping movements. Muscle strength in muscle contractions that actually lead to limb movement (eccentric or concentric) might therefore be more relevant for fall prevention than strength in a muscle contraction that does not lead to limb movement (isometric). |
| Center of pressure (CoP) measurement:  **Unilateral** vs. bilateral stance | (e) Maintaining balance in unilateral stance is more difficult than in bilateral stance, and postural instability is more likely to be detected in middle-aged adults [9]. |
| Center of pressure (CoP) measurement:  **95% confidence ellipse (mm2)** vs. Rambling (mm2) vs. Trembling (mm2) | (f) No evidence suggesting superiority of any of these CoP measurements was found. |
| Dynamometer velocity: **180°/s** vs. 60°/s | (a) Reactive fall prevention requires rapid stepping movements. No evidence showing that slower movements might be more relevant than fast movements was found. |
| Star-excursion balance tests (SEBT) vs.  **Functional reach test (FRT)** | (d) The FRT has a single outcome that can easily be included in the analysis. The SEBT has six outcome measures and it is unclear whether it is valid to use an average of these measures. |

*The bold mark reflects the criterion chosen to be more relevant in the context of this review.*

**The authors selected the outcome measures that (a) they thought were most relevant to falls prevention, (b) they thought were most relevant for activities of daily living, (c) that were used particularly frequently in the included studies to reduce heterogeneity, (d) that were favorable in terms of data analysis, (e) were more challenging and therefore more likely to be accurate for middle-aged adults. In some cases (f) the choice seemed to make no difference but was standardized to reduce heterogeneity.*

**Supplement 5: Included and excluded studies in fulltext screening**

| \| **Abbreviation** \| **Description** \| **Number of studies** \| \| --- \| --- \| --- \| \| no fulltext \| *Full-text was not available* \| 24 \| \| included \| *Study was included* \| 66 \| \| age \| *The mean age or confidence intervals were not appropriate* \| 186 \| \| health \| *The study population was not healthy according to our definition (please see supplement 3)* \| 18 \| \| master runners \| *The study population were master runners* \| 1 \| \| no RCT \| *The study design was no randomized controlled trial* \| 14 \| \| control \| *The control group was not adequate or results from the control group were not reported* \| 4 \| \| outcome \| *The study measured no outcome with relevance for our analysis* \| 30 \| \| intervention \| *The intervention applied did not fit with our eligibility criteria* \| 6 \| \| language \| *The study was not available in English or German* \| 11 \| \| missing data \| *The data report were insufficient to include for our analysis* \| 19 \| \| Data appears elsewhere \| *The data were already reported in other publication we had included in our analysis* \| 5 \| | | | |
| --- | --- | --- | --- | --- | --- | --- | --- | --- | --- | --- | --- | --- | --- | --- | --- | --- | --- | --- | --- | --- | --- | --- | --- | --- | --- | --- | --- | --- | --- | --- | --- | --- | --- | --- | --- | --- | --- | --- | --- | --- | --- | --- |
| ***Author*** | ***Year*** | ***Title*** | ***Included / Reason for exclusion*** |
| *Abbasi et al.* | *2012* | *Effects of aquatic balance training and detraining on neuromuscular performance and balance in healthy middle aged male* | *no fulltext* |
| *Abe, T. D., D. V.; Pollock, M. L.; Garzarella, L.* | *2000* | *Time course for strength and muscle thickness changes following upper and lower body resistance training in men and women* | *age* |
| *Adams, K. J. S., A. M.; Berning, J. M.; Sevene-Adams, P. G.; Barnard, K. L.; Shimp-Bowerman, J.* | *2001* | *Progressive strength training in sedentary, older African American women* | *included* |
| *Agner, V. F. a. C. G., Marcia Carvalho; Taffarel, Andre Andriolli; Mourão, Camila Baldini; da Silva, Isabel Paulo; da Silva, Sara Pereira; Peccin, Maria Stella; Jr.Lombardi, Império* | *2018* | *Effects of concurrent training on muscle strength in older adults with metabolic syndrome: A randomized controlled clinical trial* | *age* |
| *Almarzouki, R. B., G.; Lohman, E.; Bradley, B.; Nelson, T.; Alqabbani, S.; Alonazi, A.; Daher, N.* | *2020* | *Improved balance in middle-aged adults after 8 weeks of a modified version of Otago Exercise Program: A randomized controlled trial* | *included* |
| *Alonso-Domínguez, R.; Sánchez-Aguadero, N.; Llamas-Ramos, I.; Lugones-Sánchez, C.; González-Sánchez, S.; Gómez-Marcos, M.; García-Ortiz, L.* | *2021* | *Effect of an intensive intervention on the increase of physical activity and the decrease of sedentary lifestyle in inactive postmenopausal* | *Missing data* |
| *Alvani, E.; Shirvani, H.; Shamsoddini, A.* | *2021* | *Neuromuscular exercises on pain intensity, functional disability, proprioception, and balance of military personnel with chronic low back pain* | *age* |
| *Amaro-Gahete, F. J. D.-l., O. A.; Jurado-Fasoli, L.; Dote-Montero, M.; Gutiérrez, Á; Ruiz, J. R.; Castillo, M. J.* | *2019* | *Changes in Physical Fitness After 12 Weeks of Structured Concurrent Exercise Training, High Intensity Interval Training, or Whole-Body Electromyostimulation Training in Sedentary Middle-Aged Adults: A Randomized Controlled Trial* | *missing data* |
| *Ando, D. H., Y.; Suzuki, K.; Yamagata, Z.* | *2009* | *Effects of exercise training on circulating high molecular weight adiponectin and adiponectin oligomer composition: a randomized controlled trial* | *control* |
| *Andrusaitis et al.* | *2011* | *Trunk stabilization among women with chroniclower back pain: a randomized, controlled, andblinded pilot study* | *missing data* |
| *Anguera, J.A.; Volponi, J.J.; Simon, A.J.; Gallen, C.; Rolle, C.; Anguera-Singka, R.; Pitsch, E.; Thompson, C.; Gazzaley, A.* | *2021* | *Integrated cognitive and physical fitness training enhances attention abilities in older adults* | *age* |
| *Anek, A. B., N.* | *2015 (a)* | *Effects of Circuit Aerobic Step Exercise Program on Musculoskeletal for Prevention of Falling and Enhancement of Postural Balance in Postmenopausal Women* | *included* |
| *Anek, A. K., V.; Bunyaratavej, N.* | *2011* | *Effects of the circuit box jumping on bone resorption, health-related to physical fitness and balance in the premenopausal women* | *no fulltext* |
| *Anek, A. K., V.; Bunyaratavej, N.* | *2015 (b)* | *Effects of Aerobic Step Combined with Resistance Training on Biochemical Bone Markers, Health-Related Physical Fitness and Balance in Working Women* | *included* |
| *Anton, M. M. C.-C., M. Y.; DeVan, A. E.; Neidre, D. B.; Cook, J. N.; Tanaka, H.* | *2006* | *Resistance training increases basal limb blood flow and vascular conductance in aging humans* | *missing data* |
| [*Aragão*](https://europepmc.org/search?query=AUTH%3A%22Jos%C3%A9%20C%20Arag%C3%A3o-Santos%22)*-Santos, J. C. D. R.-N., A. G.; Nogueira, A. C.; Feitosa-Neta, M. D. L.; Brandão, L. H.; Chaves, L. M.; Da Silva-Grigoletto, M. E.* | *2019* | *The effects of functional and traditional strength training on different strength parameters of elderly women: a randomized and controlled trial* | *age* |
| *Araújo, J. P. N., G. R.; Loenneke, J. P.; Bemben, M. G.; Laurentino, G. C.; Batista, G.; Silva, J. C.; Freitas, E. D.; Sousa, M. S.* | *2015* | *The effects of water-based exercise in combination with blood flow restriction on strength and functional capacity in post-menopausal women* | *included* |
| *Araujo, T. B. S., N. A.; Costa, J. N.; Pereira, M. M.; Safons, M. P.* | *2011* | *Effect of equine-assisted therapy on the postural balance of the elderly* | *age* |
| *Areeudomwong, P. S., S.; Phuttanurattana, N.; Sripoom, P.; Buttagat, V.; Keawduangdee, P.* | *2019* | *Balance and functional fitness benefits of a Thai boxing dance program among community-dwelling older adults at risk of falling: A randomized controlled study* | *age* |
| *Ashari et al.* | *2016* | *Effectiveness of Individualized Home-Based Exercise on Turning and Balance Performance Among Adults Older than 50 yrs* | *age* |
| *Asikainen, T. M. S., J. H.; Pasanen, M. E.; Oja, P.; Rinne, M. B.; Miilunpalo, S. I.; Nygård, C. H.; Vuori, I. M.* | *2006* | *Effect of brisk walking in 1 or 2 daily bouts and moderate resistance training on lower-extremity muscle strength, balance, and walking performance in women who recently went through menopause: a randomized, controlled trial* | *included* |
| *Avelar, N. C. B., A. C.; Alcântara, M. A.; Gomes, W. F.* |  | *Effectiveness of aquatic and non-aquatic lower limb muscle endurance training in the static and dynamic balance of elderly people* | *age* |
| *Azarpaikan, A. T. T., Hamidreza* | *2018* | *Effect of somatosensory and neurofeedback training on balance in older healthy adults: a preliminary investigation* | *age* |
| *Baldon Rde, M. L., D. F.; Carvalho, L. P.; Wun, P. Y.; Santiago, P. R.; Serrão, F. V.* | *2012* | *Effect of functional stabilization training on lower limb biomechanics in women* | *age* |
| *Baltich, J. E., C. A.; Whittaker, J. L.; Nigg, B. M.* | *2017* | *Running injuries in novice runners enrolled in different training interventions: a pilot randomized controlled trial* | *age* |
| *Baltich, J. E., Carolyn A.; Stefanyshyn, Darren; Nigg, Benno M.* | *2014* | *The effects of isolated ankle strengthening and functional balance training on strength, running mechanics, postural control and injury prevention in novice runners: design of a randomized controlled trial* | *age* |
| *Barene, S.; Holtermann, A.; Oseland, H.; Brekke, O-L.; Krustrup, P.* | *2016* | *Effects on muscle strength, maximal jump height, flexibility and postural sway after soccer and Zumba exercise among female hospital employees: a 9-month randomised controlled trial* | *missing data* |
| *Batrakoulis, A.; Jamurtas, A.; Tsimeas, P.; Poulios, A.; Perivoliotis, K.; Syrou, N.; Papanikolaou, K.; Draganidis, D.; Deli, C.; Metsios, G.; Angelopoulos, T.; Feito, Y., Fatouros, I.* | *2022* | *Hybrid-type, multicomponent interval training upregulates musculoskeletal fitness of adults with overweight and obesity in a volume-dependent manner: A 1-year dose-response randomised controlled trial* | *included* |
| *Behrens, M. M.-M., A.; Bruhn, S.* | *2014* | *Effect of Plyometric Training on Neural and Mechanical Properties of the Knee Extensor Muscles* | *age* |
| *Bemben, D. A. F., N. L.; Bemben, M. G.; Nabavi, N.; Koh, E. T.* | *2000* | *Musculoskeletal responses to high- and low-intensity resistance training in early postmenopausal women* | *included* |
| *Bento, P. C. P., G.; Ugrinowitsch, C.; Rodacki, A. Lf* | *2012* | *The effects of a water-based exercise program on strength and functionality of older adults* | *age* |
| *Bergström, I. g., B. M.; Pyykkö, I.* | *2007* | *Training or EPT in perimenopause on balance and flushes* | *missing data* |
| *Berryman, N. M., D. B.; Bosquet, L.* | *2010* | *Effect of plyometric vs. dynamic weight training on the energy cost of running* | *age* |
| *Bezerra, E. S. O., Lbdr; de Moura, B. M.; Willardson, J. M.; Simão, R.; Moro, A. R. P.* | *2018* | *Mixed Session Periodization as a New Approach for Strength, Power, Functional Performance, and Body Composition Enhancement in Aging Adults* | *age* |
| *Bizheh et al.* | *2011* | *The acute effects of strength training on inflammatory markers predicting atherosclerosis: a study on inactive middle-aged men* | *language* |
| *Bocalini, D. S. S., A. J.; dos Santos, L.; Murad, N.; Levy, R. F.* | *2009* | *Strength training preserves the bone mineral density of postmenopausal women without hormone replacement therapy* | *age* |
| *Bogaerts, A. V., S.; Delecluse, C.; Claessens, A. L.; Boonen, S.* | *2007* | *Effects of whole body vibration training on postural control in older individuals: a 1 year randomized controlled trial* | *age* |
| *Bolam, K. A. S., T. L.; Jenkins, D. G.; Galvao, D. A.; Taaffe, D. R.* | *2016* | *The Osteogenic Effect of Impact-Loading and Resistance Exercise on Bone Mineral Density in Middle-Aged and Older Men: A Pilot Study* | *age* |
| *Bolton, K. L. E., T.; Wark, J.; Wee, E.; Matthews, B.; Kelly, A.; Craven, R.; Kantor, S.; Bennell, K. L.; Bolton, Karen L.; Egerton, Thorlene; Wark, John; Wee, Elin; Matthews, Bernadette; Kelly, Anne; Craven, Robyn; Kantor, Sue; Bennell, Kim L.* | *2012* | *Effects of exercise on bone density and falls risk factors in post-menopausal women with osteopenia: a randomised controlled trial* | *included* |
| *Bonfante, I. L. C.-M., M. P.; Brunelli, D. T.; Gáspari, A. F.; Duft, R. G.; Lopes, W. A.; Bonganha, V.; Libardi, C. A.; Cavaglieri, C. R.* | *2017* | *Combined training, FNDC5/irisin levels and metabolic markers in obese men: A randomised controlled trial* | *included* |
| *Boraczyński, M. T. B., T. W.; Wójcik, Z.; Gajewski, J.; Laskin, J. J.* | *2020* | *The effects of a 6-month moderate-intensity Hatha yoga-based training program on health-related fitness in middle-aged sedentary women: a randomized controlled study* | *age* |
| *Borba-Pinheiro, C. J. D., E. H.; Vale, R. G.; Drigo, A. J.; Carvalho, M. C.; Tonini, T.; Meza, E. I.; Figueiredo, N. M.* | *2016* | *Resistance training programs on bone related variables and functional independence of postmenopausal women in pharmacological treatment: A randomized controlled trial* | *age* |
| *Borges, E. G. C., S. A.; Vale, R. G.; Cruz, T. H.; Carvalho, M. C.; Pinto, F. M.; Dantas, E. H.* | *2012* | *The effect of ballroom dance on balance and functional autonomy among the isolated elderly* | *age* |
| *Bouillon, L. E. S., D. K.; Driver, A. C.* | *2009* | *Comparison of training between 2 cycle ergometers on dynamic balance for middle-aged women* | *intervention* |
| *Brandon, L. J. B., L. W.; Lloyd, A.; Gaasch, D. A.* | *2004* | *Resistive training and long-term function in older adults* | *age* |
| *Brand , R. S., W.; Grossman, K.; Duhnsen, R.* | *2006* | *Effects of a physical exercise intervention on employees'perceptions quality of life: a randomized controlled trial* | *outcome* |
| *Brown, J. C. S., K. H.* | *2015* | *Weight lifting and appendicular skeletal muscle mass among breast cancer survivors: a randomized controlled trial* | *health* |
| *Brunelli D et al.* | *2015* | *Combined Training Reduces Subclinical Inflammation in Obese Middle-Age Men* | *included* |
| *Burger, C. S., V.; Lindner, C.; Radlinger, L.; Elfering, A.* | *2012* | *Stochastic resonance training reduces musculoskeletal symptoms in metal manufacturing workers: a controlled preventive intervention study* | *age* |
| *Campos De Oliveira, L. A. D. A. P.-O., Deise; Alves Do Prado, Rafaela Claudia; Pereira D'avanso De Oliveira, Danielle; Del Antônio, Tiago; De Oliveira, Rodrigo Franco; Gonçalves De Oliveira, Raphael* | *2016* | *Effects of Pilates on postural balance and functional autonomy of elderly: a randomized controlled trial* | *age* |
| *Carrasco, M. V., M.* | *2012* | *Water training in postmenopausal women: effect on muscular strength* | *missing data* |
| *Chang et al.* | *2016* | *Effects of 24-week Tai Chi exercise on the knee andankle proprioception of older women* | *age* |
| *Chasland, L.; Yeap, B.; Maiorana, A.; Chan, Y.; Maslen, B.; Cooke, B.; Dembo, L.; Naylor, L.; Green, D.* | *2021* | *Testosterone and exercise: effects on fitness, body composition, and strength in middle-to-older aged men with low-normal serum testosterone levels* | *included* |
| *Chavarrias, M. C.-V., J.; Barrantes-Martín, B.; Pérez-Gómez, J.* | *2019* | *Effects of 8-week of fitness classes on blood pressure, body composition, and physical fitness* | *age* |
| *Cheng et al.* | *2020* | *Effect of Tai Chi exercise with the same frequency and different exercise duration on the bone mineral density of older women* | *no fulltext* |
| *Cherup, N. R., K.; Potiaumpai, M.; Widdowson, K.; Jaghab, A. M.; Chowdhari, S.; Armitage, C.; Seeley, A.; Signorile, J.* |  | *Improvements in cognition and associations with measures of aerobic fitness and muscular power following structured exercise* | *age* |
| *Chi, C. I.* | *2015* | *Tai Chi and Baduanjin improve cognitive function and balance capability in elderly adults* | *no fulltext* |
| *Chilibeck, P. D. V., H.; Pierson, R.; Case, A.; Olatunbosun, O.; Whiting, S. J.; Beck, T. J.; Pahwa, P.; Biem, H. J.* |  | *Effect of exercise training combined with isoflavone supplementation on bone and lipids in postmenopausal women: a randomized clinical trial* | *included* |
| *Chow et al.* | *1987* | *Effect of two randomised exercise programmes on bone mass of healthy postmenopausal women* | *outcome* |
| *Colado, J. C. T., N. T.* | *2008* | *Effects of a short-term resistance program using elastic bands versus weight machines for sedentary middle-aged women* | *included* |
| *Colado, J. C. T., N. T.; Tella, V.; Saucedo, P.; Abellán, J.* | *2009* | *Effects of aquatic resistance training on health and fitness in postmenopausal women* | *included* |
| *Conceição, M. S. B., V.; Vechin, F. C.; Berton, R. P.; Lix; rão, M. E.; Nogueira, F. R.; de Souza, G. V.; Chacon-Mikahil, M. P.; Libardi, C. A.* | *2013* | *Sixteen weeks of resistance training can decrease the risk of metabolic syndrome in healthy postmenopausal women* | *included* |
| *Cormie, P. M., G. O.; McBride, J. M.* | *2007* | *Power versus strength-power jump squat training: influence on the load-power relationship* | *age* |
| *Correa, C. S. T., B. C.; Bittencourt, A.; Lemos, L.; Marques, N. R.; Radaelli, R.; Kruger, R. L.; Reischak-Oliveira, A.; Pinto, R. S.* | *2014* | *Effects of high and low volume of strength training on muscle strength, muscle volume and lipid profile in postmenopausal women* | *age* |
| *Correa, C. S. T., Bruno Costa; Cobos, Roberto Carlos Rebolledo; Macedo, Rodrigo Cauduro Oliveira; Kruger, Renata Lopes; Carteri, R; all Bruce Kreismann; Radaelli, Régis; Gross, Julia Silveira; Pinto, Ronei Silveira; Reischak-Oliveira, Álvaro* | *2015* | *High-volume resistance training reduces postprandial lipaemia in postmenopausal women* | *included* |
| *Cortez-Cooper, M. Y. A., M. M.; Devan, A. E.; Neidre, D. B.; Cook, J. N.; Tanaka, H.* | *2008* | *The effects of strength training on central arterial compliance in middle-aged and older adults* | *missing data* |
| *Costa, R. R. K., A. C.; Reichert, T.; Prado, A. K. G.; Coconcelli, L.; Buttelli, A. C. K.; Pereira, L. F.; Masiero, M. P. B.; Meinerz, A. P.; Conceição, M. O.; Sbeghen, I. L.; Kruel, L. F. M.* | *2018* | *Water-based aerobic training improves strength parameters and cardiorespiratory outcomes in elderly women* | *age* |
| *Cotofana, S. R.-D., S.; Hudelmaier, M.; Himmer, M.; Wirth, W.; Sänger, A. M.; Eckstein, F.* | *2010* | *Effects of exercise intervention on knee morphology in middle-aged women: a longitudinal analysis using magnetic resonance imaging* | *included* |
| *Coubard et al.* | *2014* | *One month of contemporary dance modulates fractal posture in aging* | *age* |
| *Cox et al.* | *1996* | *DETERMINANTS OF CHANGE IN BLOOD PRESSURE DURING S.W.E.A.T.: THE SEDENTARY WOMEN EXERCISE ADHERENCE TRIAL* | *no RCT* |
| *Cristea, A. K., M. T.; Häkkinen, K.; Mero, A.; Alén, M.; Sipilä, S.; Viitasalo, J. T.; Koljonen, M. J.; Suominen, H.; Larsson, L.* | *2008* | *Effects of combined strength and sprint training on regulation of muscle contraction at the whole-muscle and single-fibre levels in elite master sprinters* | *age* |
| *Cunha, P. M. N., J. P.; Tomeleri, C. M.; Nascimento, M. A.; Schoenfeld, B. J.; Antunes, M.; Gobbo, L. A.; Teixeira, D.; Cyrino, E. S.* | *2020* | *Resistance Training Performed With Single and Multiple Sets Induces Similar Improvements in Muscular Strength, Muscle Mass, Muscle Quality, and IGF-1 in Older Women: A Randomized Controlled Trial* | *age* |
| *Cussler, E. C. L., T. G.; Going, S. B.; Houtkooper, L. B.; Metcalfe, L. L.; Flint-Wagner, H. G.; Harris, R. B.; Teixeira, P. J.* | *2003* | *Weight lifted in strength training predicts bone change in postmenopausal women* | *outcome* |
| *Ćwirlej-Sozańska, A. W.-S., Agnieszka; Wilmowska-Pietruszyńska, Anna; Drużbicki, Mariusz; Wołoszyn, Natalia; Guzik, Agnieszka; Sozański, Bernard* | *2018* | *Evaluation of the Effect of 16 Weeks of Multifactorial Exercises on the Functional Fitness and Postural Stability of a Low-Income Elderly Population* | *age* |
| *Da Silva Chaves, L. M. D. R.-N., Antônio Gomes; Costa Nogueira, Albernon; Aragão-Santos, José Carlos; Albuquerque Br; ão, Le; ro Henrique; Da Silva-Grigoletto, Marzo Edir* | *2017* | *Influence of functional and traditional training on muscle power, quality of movement and quality of life in the elderly: a randomized and controlled clinical trial* | *age* |
| *Dalager, T. J., J. B.; Sjøgaard, G.* | *2017* | *Intelligent Physical Exercise Training in a Workplace Setting Improves Muscle Strength and Musculoskeletal Pain: A Randomized Controlled Trial* | *outcome* |
| *Daly, R. M.; Dalla Via, J.; Fyfe, J.J.; Nikander, R.; Kukuljan, S.* | *2021* | *Effects of exercise frequency and training volume on bone changes following a multi-component exercise intervention in middle aged and older men: Secondary analysis of an 18-month randomized controlled trial* | *Age* |
| *Davidson, L. E. H., R.; Kilpatrick, K.; Kuk, J. L.; McMillan, K.; Janiszewski, P. M.; Lee, S.; Lam, M.; Ross, R.* | *2009* | *Effects of exercise modality on insulin resistance and functional limitation in older adults: a randomized controlled trial* | *age* |
| *de Carvalho Bastone, A. r. N., Luciana Neri; de Souza Moreira, Bruno; Rosa, Iramaya Francielle; Ferreira, Gabrielle Bemfica; Santos, Dayane Deyse Lee; Monteiro, Nancy Krysna Sancha Silva; Alves, Michelle Dullya; ra, Rômulo Amaral; de Lira, Elane Marinho* | *2020* | *Independent and combined effect of home-based progressive resistance training and nutritional supplementation on muscle strength, muscle mass and physical function in dynapenic older adults with low protein intake: A randomized controlled trial* | *age* |
| *de Jong, J. L., K. A.; Stevens, M.; de Greef, M. H.; Rispens, P.; King, A. C.; Mulder, T.* | *2006* | *Six-month effects of the Groningen active living model (GALM) on physical activity, health and fitness outcomes in sedentary and underactive older adults aged 55-65* | *included* |
| *de Ruiter, C. J. H., Vana; Icke, Chris; Groen, Bart; Gemmink, Anne; Smilde, Hiltsje; de Haan, Arnold* | *2012* | *The effects of imagery training on fast isometric knee extensor torque development* | *age* |
| *Deibert, P. S., F.; König, D.; Vitolins, M. Z.; Dickhuth, H. H.; Gollhofer, A.; Berg, A.* | *2011* | *Soy protein based supplementation supports metabolic effects of resistance training in previously untrained middle aged males* | *included* |
| *De Oliveira Júnior, G.; De Sousa, J.; Carneiro, M.; Martins, F.; Santagnello, S.; Orsatti, F.* | *2021* | *Resistance training-induced improvement in exercise tolerance is not dependent on muscle mass gain in post-menopausal women* | *age* |
| *Devaraj, S.; Rockette-Wagner, B.; Miller, R.; Arena, V.; Napoleone, J.; Conroy, M.; Kriska, A.;* | *2021* | *The Impact of a Yearlong Diabetes Prevention Program-Based Lifestyle Intervention on Cardiovascular Health Metrics* | *age* |
| *Del Vecchio, L. S., R.; Reaburn, P.; Macgregor, C.; Meerkin, J.; Villegas, J.; Korhonen, M. T.* | *2019* | *Effects of Combined Strength and Sprint Training on Lean Mass, Strength, Power, and Sprint Performance in Masters Road Cyclists* | *no RCT* |
| *Delecluse, C. C., V.; Roelants, M.; Verschueren, S.; Derave, W.; Ceux, T.; Eijnde, B. O.; Seghers, J.; Pardaens, K.; Brumagne, S.; Goris, M.; Buekers, M.; Spaepen, A.; Swinnen, S.; Stijnen, V.* | *2004* | *Exercise programs for older men: mode and intensity to induce the highest possible health-related benefits* | *age* |
| *De Sevilla, G.; Guido; O.; De la Cruz, M.; Fernández, A.; Alejo, L.; Martínez, M.; Pérez-Ruiz, M.* | *2021* | *Adherence to a Lifestyle Exercise and Nutrition Intervention in University Employees during the COVID-19 Pandemic: A Randomized Controlled Trial* | *age* |
| *Dobbs, T. J. S., S. R.; Conger, S. A.* | *2018* | *Improving Power Output in Older Adults Using Plyometrics in a Body Mass-Supported Treadmill* | *age* |
| *Donath, L. F., O.; Schefer, Y.; Roth, R.; Zahner, L.* | *2015* | *Repetitive daily point of choice prompts and occupational sit-stand transfers, concentration and neuromuscular performance in office workers: an RCT* | *age* |
| *Donges, C. E. D., R.* | *2012* | *Effects of resistance or aerobic exercise training on total and regional body composition in sedentary overweight middle-aged adults* | *no RCT* |
| *Donges, C. E. D., R.; Guelfi, K. J.; Smith, G. C.; Adams, D. R.; Edge, J. A.* | *2013* | *Comparative effects of single-mode vs. duration-matched concurrent exercise training on body composition, low-grade inflammation, and glucose regulation in sedentary, overweight, middle-aged men* | *included* |
| *Dos Santos, E. S. A., R. Y.; Filho, I. G.; Lopes, N. L.; Panelli, P.; Nascimento Dda, C.; Collier, S. R.; Prestes, J.* | *2014* | *Acute and chronic cardiovascular response to 16 weeks of combined eccentric or traditional resistance and aerobic training in elderly hypertensive women: a randomized controlled trial* | *age* |
| *Duft, R. G. C., A.; Bonfante, I. L. P.; Brunelli, D. T.; Chacon-Mikahil, M. P. T.; Cavaglieri, C. R.* | *2017* | *Metabolomics Approach in the Investigation of Metabolic Changes in Obese Men after 24 Weeks of Combined Training* | *outcome* |
| *Elavsky, S. M., E.* | *2007* | *Exercise and self-esteem in menopausal women: a randomized controlled trial involving walking and yoga* | *outcome* |
| *Elliott, K. J. S., C.; Cable, N. T.* | *2002* | *Effects of resistance training and detraining on muscle strength and blood lipid profiles in postmenopausal women* | *included* |
| *Emilio, E. J. M.-L. H.-C., Fidel; Jiménez-Lara, Pilar M.; Latorre-Román, Pedro; Martínez-Amat, Antonio* | *2014* | *The Association of Flexibility, Balance, and Lumbar Strength with Balance Ability: Risk of Falls in Older Adults* | *no RCT* |
| *Evans, W.* | *1997* | *Functional and metabolic consequences of sarcopenia* | *no RCT* |
| *Espuch-Oliver et al.* | *2019* | *Effects of different exercise modalities on S-Klotho plasma levels in middle-aged sedentary adults* | *outcome* |
| *Fang, Y. Y. H., C. Y.; Hsu, M. C.* | *2019* | *Effectiveness of a physical activity program on weight, physical fitness, occupational stress, job satisfaction and quality of life of overweight employees in high-tech industries: a randomized controlled study* | *age* |
| *Federici, A. B., S.; Rocchi, M. B.* | *2005* | *Does dance-based training improve balance in adult and young old subjects? A pilot randomized controlled trial* | *age* |
| *Ferreira, F. R. A., G.; Confessor, Y. Q.; Gagliardi, J. F. L.; Neto, T. L. D.* | *2013* | *Acute Effect of Extensors Knee Unilateral on Leg Extension Machine with and without Stimulation on the Vibrating Platform* | *age* |
| *Ferreira, D.; Christofoletti, G.; Campos, D.; Janducci, A.; Candanedo, M.; Ansai, J.* | *2022* | *Effects of Aquatic Physical Exercise on Motor Risk Factors for Falls in Older People During the COVID-19 Pandemic: A Randomized Controlled Trial* | *age* |
| *Figueroa, A. G., S. B.; Milliken, L. A.; Blew, R. M.; Sharp, S.; Teixeira, P. J.; Lohman, T. G.* | *2003* | *Effects of exercise training and hormone replacement therapy on lean and fat mass in postmenopausal women* | *health* |
| *Figueroa, A. K., R.; Madzima, T. A.; Wong, A.* | *2014* | *Effects of whole-body vibration exercise training on aortic wave reflection and muscle strength in postmenopausal women with prehypertension and hypertension* | *Data appears elsewhere* |
| *Figueroa, A. K., R.; Madzima, T. A.; Wong, A.* | *2014* | *Whole-body vibration exercise training reduces arterial stiffness in postmenopausal women with prehypertension and hypertension* | *included* |
| *Figueroa, A. P., S. Y.; Seo, D. Y.; Sanchez-Gonzalez, M. A.; Baek, Y. H.* | *2011* | *Combined resistance and endurance exercise training improves arterial stiffness, blood pressure, and muscle strength in postmenopausal women* | *included* |
| *Finianos, B.* | *2021* | *The effects of a 1-year recreational football protocol on bone mineral density and physical performance parameters in a group of healthy inactive 50 years old men* | *no fulltext* |
| *Flandez, J. B. o., N.; Gargallo, P.; Fernández-Garrido, J.; Vargas-Foitzick, R. A.; Devis-Devis, J.; Colado, J. C.* | *2017* | *Metabolic and Functional Profile of Premenopausal Women With Metabolic Syndrome After Training With Elastics as Compared to Free Weights* | *included* |
| *Foley, A. H., S.; Barnard, R.* | *2011* | *Effectiveness of once-weekly gym-based exercise programmes for older adults post discharge from day rehabilitation: a randomised controlled trial* | *age* |
| *Frye, B. S., S.; Kemarskaya, T.; Pruchno, R.* | *2007* | *Tai chi and low impact exercise: effects on the physical functioning and psychological well-being of older people* | *age* |
| *Fu, S. C., N. L.; Nitz, J.* | *2009* | *Controlling balance decline across the menopause using a balance-strategy training program: a randomized, controlled trial* | *included* |
| *Fukuchi, R. K. S., Darren J.; Stirling, Lisa; Ferber, Reed* | *2016* | *Effects of strengthening and stretching exercise programmes on kinematics and kinetics of running in older adults: a randomised controlled trial* | *missing data* |
| *Gába, A. C., R.; Svoboda, Z.; Chmelík, F.; Pelclová, J.; Lehnert, M.; Frömel, K.* | *2016* | *The effect of brisk walking on postural stability, bone mineral density, body weight and composition in women over 50 years with a sedentary occupation: a randomized controlled trial* | *intervention* |
| *Galantino, M. L. B., T. M.; Eissler-Russo, J. L.; Holbrook, M. L.; Mogck, E. P.; Geigle, P.; Farrar, J. T.* | *2004* | *The impact of modified Hatha yoga on chronic low back pain: a pilot study* | *health* |
| *Galiano-Castillo, N. C.-V., I.; Fernández-Lao, C.; Ariza-García, A.; Díaz-Rodríguez, L.; Del-Moral-Ávila, R.; Arroyo-Morales, M.* | *2016* | *Telehealth system: A randomized controlled trial evaluating the impact of an internet-based exercise intervention on quality of life, pain, muscle strength, and fatigue in breast cancer survivors* | *health* |
| *García-Pinillos, F. L.-F., Carlos; Latorre-Román, Pedro A.; Pantoja-Vallejo, Antonio; Ramirez-Campillo, Rodrigo* | *2020* | *Jump-Rope Training: Improved 3-km Time-Trial Performance in Endurance Runners via Enhanced Lower-Limb Reactivity and Foot-Arch Stiffness* | *age* |
| *Gargallo, P. C., J. C.; Juesas, A.; Hern; o-Espinilla, A.; Estañ-Capell, N.; Monzó-Beltran, L.; García-Pérez, P.; Cauli, O.; Sáez, G. T.* | *2018* | *The Effect of Moderate- Versus High-Intensity Resistance Training on Systemic Redox State and DNA Damage in Healthy Older Women* | *age* |
| *Gates, N. J. V., Michael; Sachdev, Perminder S.; Singh, Nalin A.; Baune, Bernhard T.; Brodaty, Henry; Suo, Chao; Jain, Nidhi; Wilson, Guy C.; Wang, Yi; Baker, Michael K.; Williamson, Dominique; Foroughi, Nasim; Fiatarone Singh, Maria A.* | *2011* | *Study of Mental Activity and Regular Training (SMART) in at risk individuals: a randomised double blind, sham controlled, longitudinal trial* | *age* |
| *Gennuso, K. P. Z., K.; Cashin, S. E.; Strath, S. J.* | *2013* | *Resistance training congruent with minimal guidelines improves function in older adults: a pilot study* | *age* |
| *Gill, A. A. V., V. L.; Shuster, J. J.; Notelovitz, M.* | *1984* | *A well woman's health maintenance study comparing physical fitness and group support programs* | *intervention* |
| *Gillett, P. A. C., M. S.; White, A. T.; Martinson, L.* | *1995* | *Responses of 49- to 59-year-old sedentary, overweight women to four months of exercise conditioning and/or fitness education* | *included* |
| *Giorgi, M. P., M.* | *2015* | *EFFETTI INDOTTI DALLA CORE STABILITY SUGLI SPRINTER: UNO STUDIO PILOTA* | *language* |
| *Grabiner, M. D. B., M. L.; Gatts, S.; Marone, J.; Troy, K. L.* | *2012* | *Task-specific training reduces trip-related fall risk in women* | *age* |
| *Granacher, U. W., C.; Rueck, N.; Esposito, C.; Roth, R.; Zahner, L.* | *2011* | *Promoting balance and strength in the middle-aged workforce* | *included* |
| *Hagstrom, A. D. M., P. W.; Lonsdale, C.; Papalia, S.; Cheema, B. S.; Toben, C.; Baune, B. T.; Fiatarone Singh, M. A.; Green, S.* | *2016* | *The effect of resistance training on markers of immune function and inflammation in previously sedentary women recovering from breast cancer: a randomized controlled trial* | *health* |
| *Hall, C. D. H.-G., L.; Tusa, R. J.; Herdman, S. J.* | *2010* | *Efficacy of gaze stability exercises in older adults with dizziness* | *age* |
| *Hamrick, I. M., Paul; Christopher, Nate; Smith, Paul D.* | *2017* | *Yoga's effect on falls in rural, older adults* | *age* |
| *Han, K. R., Mark D.* | *2011* | *Effects of 4 Weeks of Elastic-Resistance Training on Ankle-Evertor Strength and Latency* | *age* |
| *Hass, C. J. G., L.; de Hoyos, D. V.; Connaughton, D. P.; Pollock, M. L.* | *2001* | *Concurrent improvements in cardiorespiratory and muscle fitness in response to total body recumbent stepping in humans* | *missing data* |
| *Hawkins et al* | *2002* | *The relationship between bone adaptations to resistance exercise and reproductive-hormone levels* | *no RCT* |
| *Head, P. A., B.; Browne, D.; Campkin, T.; Barcellona, M.* | *2015* | *Effect of practical blood flow restriction training during bodyweight exercise on muscular strength, hypertrophy and function in adults: a randomised controlled trial* | *age* |
| *Heinonen, A. K., P.; Sievänen, H.; Oja, P.; Pasanen, M.; Rinne, M.; Uusi-Rasi, K.; Vuori, I.* | *1996* | *Randomised controlled trial of effect of high-impact exercise on selected risk factors for osteoporotic fractures* | *age* |
| *Heinonen, A. M., Jyri; Kannus, Pekka; Uusi-Rasi, Kirsti; Nik; er, Riku; Kontulainen, Saija; Sievänen, Harri* | *2012* | *Effects of High-Impact Training and Detraining on Femoral Neck Structure in Premenopausal Women: A Hip Structural Analysis of an 18-Month Randomized Controlled Exercise Intervention with 3.5-Year Follow-Up* | *age* |
| *Helge, E. W. A., P.; Jakobsen, M. D.; Sundstrup, E.; ers, M. B.; Karlsson, M. K.; Krustrup, P.* | *2010* | *Recreational football training decreases risk factors for bone fractures in untrained premenopausal women* | *age* |
| *Helge, E. W. e., M. B.; Hornstrup, T.; Nielsen, J. J.; Blackwell, J.; Jackman, S. R.; Krustrup, P.* | *2014* | *Street football is a feasible health-enhancing activity for homeless men: Biochemical bone marker profile and balance improved* | *age* |
| *Herrero, F. S. J., A. F.; Fleck, S. J.; Balmer, J.; Pérez, M.; Cañete, S.; Earnest, C. P.; Foster, C.; Lucía, A.* | *2006* | *Combined aerobic and resistance training in breast cancer survivors: A randomized, controlled pilot trial* | *health* |
| *Hespel et al.* | *1988* | *Changes in erythrocyte sodium and plasma lipids associated with physical training* | *no fulltext* |
| *Ho, H. C. Y. M., M. W.; Wan, A.; Yew, C. W.; Lam, T. H.* | *2020* | *A cluster randomized controlled trial of a positive physical activity intervention* | *age* |
| *Holviala, J. H., A.; Alen, M.; Sallinen, J.; Kraemer, W.; Häkkinen, K.* | *2014* | *Effects of prolonged and maintenance strength training on force production, walking, and balance in aging women and men* | *included* |
| *Hu, M. F., T.; Sedliak, M.; Zhou, W.; Alen, M.; Cheng, S.* | *2008* | *Seasonal variation of red blood cell variables in physically inactive men: effects of strength training* | *age* |
| *Hu, M. F., T.; Zou, L.; Perhonen, M.; Sedliak, M.; Alen, M.; Cheng, S.* | *2009* | *Effects of strength training on work capacity and parasympathetic heart rate modulation during exercise in physically inactive men* | *age* |
| *Hughes, D. C. D., N.; Gonzalez, K.; Boggess, T.; Morris, R. M.; Ramirez, A. G.* | *2015* | *Effect of a six-month yoga exercise intervention on fitness outcomes for breast cancer survivors* | *health* |
| *Hui, G.* | *2022* | *Effect of air volleyball on muscle function of middle-aged and elderly people (2022)* | *no fulltext* |
| *Humphries, B. M., K.; Newton, R. U.; Humphries, N.* | *2001* | *Identifying bone mass and muscular changes* | *no fulltext* |
| *Hurst, C.; Weston, K.; Weston, M.* | *2019* | *The effect of 12 weeks of combined upper- and lower-body high-intensity interval training on muscular and cardiorespiratory fitness in older adults* | *age* |
| *Huschtscha, Z.; Parr, A.; Porter, J.; Costa, R.* | *2021* | *The Effects of a High-Protein Dairy Milk Beverage With or Without Progressive Resistance Training on Fat-Free Mass, Skeletal Muscle Strength and Power, and Functional Performance in Healthy Active Older Adults: A 12-Week Randomized Controlled Trial* | *age* |
| *Hsu, K.-J.; Chien, K.-Y.; Tsai, S.-C.; Tsai, Y.-S.; Liao, Y.-H.; Chen, J.-J.; Chen, Y.-R.; Chiao-Nan Chen* | *2021* | *Effects of Exercise Alone or in Combination with High-Protein Diet on Muscle Function, Aerobic Capacity, and Physical Function in Middle-Aged Obese Adults: A Randomized Controlled Tria* | *intervention* |
| *Jakobsen, M. D. S., E.; Krustrup, P.; Aagaard, P.* | *2011* | *The effect of recreational soccer training and running on postural balance in untrained men* | *age* |
| *Janzen, C. L. C., P. D.; Davison, K. S.* | *2006* | *The effect of unilateral and bilateral strength training on the bilateral deficit and lean tissue mass in post-menopausal women* | *included* |
| *Jay, K. F., D.; Hansen, K.; Zebis, M. K.; Andersen, C. H.; Mortensen, O. S.; Andersen, L. L.* | *2011* | *Kettlebell training for musculoskeletal and cardiovascular health: a randomized controlled trial* | *outcome* |
| *Jhang, L. Y. H., H. S.; Hsu, Y.; Liu, W. M.* | *2020* | *Lower Extremity Exercise Improves Functional Fitness, Physiological Indexes, Exercise Self-Efficacy, Sleep Quality, and Mental Health in Middle-Aged and Older Individuals* | *language* |
| *Jiang, Y.* | *2022* | *Influence of Square Dancing on motor function of middle-aged and elderly women* | *Age* |
| *Jiménez-García, J. D. M.-A., A.; De la Torre-Cruz, M. J.; Fábrega-Cuadros, R.; Cruz-Díaz, D.; Aibar-Almazán, A.; Achal; abaso-Ochoa, A.; Hita-Contreras, F.* | *2019* | *Suspension Training HIIT Improves Gait Speed, Strength and Quality of Life in Older Adults* | *Age* |
| *Jin, L. L., Ran; Chen, Jing; Xue, Qinbo; Yang, Yueqin* | *2017* | *Surface Electromyography Analysis of the Lower Extremities of Subjects Participating in Baduanjin Exercises* | *Age* |
| *Jørgensen, M. B. E.-A., J.; Sjøgaard, G.; Holtermann, A.; Søgaard, K.* | *2011* | *A randomised controlled trial among cleaners--effects on strength, balance and kinesiophobia* | *included* |
| *Józefowski, P. B., A.; Szafraniec, R.; Kensicka, A.* | *2015* | *[The impact of group therapeutic exercises on postural stability and the risk of falls of postmenopausal women]* | *language* |
| *Kak, H. B. C., S. H.; Lee, Y. H.; Cho, B. J.; Kim, J. W.; Oh, B. D.; Koh, H. W.* | *2013* | *A study of effect of the compound physical activity therapy on muscular strength in obese women* | *included* |
| *Kalapotharakos, V. I. T., S. P.; Smilios, I.; Michalopoulos, M.; Gliatis, J.; Godolias, G.* | *2005* | *Resistance training in older women: effect on vertical jump and functional performance* | *age* |
| *Kaminski, T. W. B., B. D.; Powers, M. E.; Hubbard, T. J.; Ortiz, C.; Kaminski, T. W.; Buckley, B. D.; Powers, M. E.; Hubbard, T. J.; Ortiz, C.* | *2003* | *Effect of strength and proprioception training on eversion to inversion strength ratios in subjects with unilateral functional ankle instability...including commentary by Mattacola CG* | *age* |
| *Kampshoff, C. S. C., M. J.; Brug, J.; Twisk, J. W.; Schep, G.; Nijziel, M. R.; van Mechelen, W.; Buffart, L. M.* | *2015* | *Randomized controlled trial of the effects of high intensity and low-to-moderate intensity exercise on physical fitness and fatigue in cancer survivors: results of the Resistance and Endurance exercise After ChemoTherapy (REACT) study* | *health* |
| *Karabulut, M. B., D. A.; Sherk, V. D.; Anderson, M. A.; Abe, T.; Bemben, M. G.* | *2011* | *Effects of high-intensity resistance training and low-intensity resistance training with vascular restriction on bone markers in older men* | *no RCT* |
| *Karakiriou, S. K. D., Helen T.; Smilios, Ilias G.; Volaklis, Konstantinos A.; Tokmakidis, Savvas P.* | *2012* | *Effects of vibration and exercise training on bone mineral density and muscle strength in post-menopausal women* | *missing data* |
| *Karatrantou, K. G., V.; Häkkinen, K.; Zafeiridis, A.* | *2017* | *Health-Promoting Effects of Serial vs. Integrated Combined Strength and Aerobic Training* | *included* |
| *Karavirta, L. H., A.; Sillanpää, E.; García-López, D.; Kauhanen, A.; Haapasaari, A.; Alen, M.; Pakarinen, A.; Kraemer, W. J.; Izquierdo, M.; Gorostiaga, E.; Häkkinen, K.* | *2011* | *Effects of combined endurance and strength training on muscle strength, power and hypertrophy in 40-67-year-old men* | *included* |
| *Karavirta, L. T., M. P.; Laaksonen, D. E.; Nyman, K.; Laukkanen, R. T.; Kinnunen, H.; Häkkinen, A.; Häkkinen, K.* | *2009* | *Heart rate dynamics after combined endurance and strength training in older men* | *Data appears elsewhere* |
| *Kaukiainen, A. N., C.; Virtanen, P.; Saloniemi, A.* | *2002* | *Physical activity intervention among unemployed male construction workers* | *age* |
| *Kawasaki, T. S., C. V.; Ozoe, N.; Higaki, H.; Kawasaki, J.* | *2011* | *A long-term, comprehensive exercise program that incorporates a variety of physical activities improved the blood pressure, lipid and glucose metabolism, arterial stiffness, and balance of middle-aged and elderly Japanese* | *age* |
| *Kell, R. T. R., A. D.; Barden, J. M.* | *2011* | *The response of persons with chronic nonspecific low back pain to three different volumes of periodized musculoskeletal rehabilitation* | *missing data* |
| *Kemmler, W. E., K.; Lauber, D.; Weineck, J.; Hensen, J.; Kalender, W. A.* | *2002* | *Exercise effects on fitness and bone mineral density in early postmenopausal women: 1-year EFOPS results* | *outcome* |
| *Kilen, A. B., Jonathan; Bejder, Jacob; Breenfeldt Andersen, Andreas; Bonne, Thomas Christian; Larsen, Pernille Dyeremose; Carlsen, Andreas; Egelund, Jon; Nybo, Lars; Mackey, Abigail Louise; Olsen, Niels Vidiendal; Aachmann-Andersen, Niels Jacob; Andersen, Jesper Løvind; Nordsborg, Nikolai Baastrup* | *2020* | *Impact of low-volume concurrent strength training distribution on muscular adaptation* | *age* |
| *Kim, S. B., M. G.; Bemben, D. A.* | *2012* | *Effects of an 8-month yoga intervention on arterial compliance and muscle strength in premenopausal women* | *included* |
| *Klentrou, P. S., J.; Roy, B.; Ladouceur, M.* | *2007* | *Effects of exercise training with weighted vests on bone turnover and isokinetic strength in postmenopausal women* | *included* |
| *Kloubec, J. A.* | *2010* | *Pilates for improvement of muscle endurance, flexibility, balance, and posture* | *included* |
| *Kolbe-Alex; er, T. L. L., E. V.; Charlton, K. E.* | *2006* | *Effectiveness of a community based low intensity exercise program for older adults* | *age* |
| *Ko et al.* | *2017* | *Whole-body vibration training improvesbalance control and sit-to-standperformance among middle-aged andolder adults: a pilot randomized controlledtrial* | *age* |
| *Kostic, R. U., S.; Purenovic-Ivanovic, T.; Miletic, D.; Katsora, G.; Pantelic, S.; Milanovic, Z.* | *2015* | *The effects of dance training program on the postural stability of middle aged women* | *included* |
| *Kraemer, W. J. K., M.; Ratamess, N. A.; Volek, J. S.; McCormick, M.; Bush, J. A.; Nindl, B. C.; Gordon, S. E.; Mazzetti, S. A.; Newton, R. U.; Gómez, A. L.; Wickham, R. B.; Rubin, M. R.; Häkkinen, K.* | *2001* | *Resistance training combined with bench-step aerobics enhances women's health profile* | *age* |
| *Yang Y, Verkuilen JV, Rosengren KS et a* | *2007* | *Effects of Yoga and an Ayurveda preparation on gait, balance and mobility in older persons* | *age* |
| *Krustrup, P. H., P. R.; Andersen, L. J.; Jakobsen, M. D.; Sundstrup, E.; ers, M. B.; Christiansen, L.; Helge, E. W.; Pedersen, M. T.; Søgaard, P.; Junge, A.; Dvorak, J.; Aagaard, P.; Bangsbo, J.* | *2010* | *Long-term musculoskeletal and cardiac health effects of recreational football and running for premenopausal women* | *age* |
| *Kubo, K. I., Y.; Suzuki, S.; Komuro, T.; Shirasawa, H.; Ishiguro, N.; Shukutani, Y.; Tsunoda, N.; Kanehisa, H.; Fukunaga, T.* | *2008* | *Effects of 6 months of walking training on lower limb muscle and tendon in elderly* | *age* |
| *Kukuljan, S. N., C. A.; Bass, S. L.; ers, K.; Nicholson, G. C.; Seibel, M. J.; Salmon, J.; Daly, R. M.* | *2009* | *Effects of a multi-component exercise program and calcium-vitamin-D3-fortified milk on bone mineral density in older men: a randomised controlled trial* | *age* |
| *Kukuljan, S. N., C. A.; ers, K.; Daly, R. M.* | *2009* | *Effects of resistance exercise and fortified milk on skeletal muscle mass, muscle size, and functional performance in middle-aged and older men: an 18-mo randomized controlled trial* | *age* |
| *Kukuljan, S. N., C. A.; ers, K. M.; Nicholson, G. C.; Seibel, M. J.; Salmon, J.; Daly, R. M.* | *2011* | *Independent and combined effects of calcium-vitamin D3 and exercise on bone structure and strength in older men: an 18-month factorial design randomized controlled trial* | *age* |
| *Lau, C. Y., Ruby; Woo, Jean* | *2015* | *Effects of a 12-Week Hatha Yoga Intervention on Cardiorespiratory Endurance, Muscular Strength and Endurance, and Flexibility in Hong Kong Chinese Adults: A Controlled Clinical Trial* | *outcome* |
| *Lau, C. Y., Ruby; Woo, Jean* | *2015* | *Effects of a 12-Week Hatha Yoga Intervention on Cardiorespiratory Endurance, Muscular Strength and Endurance, and Flexibility in Hong Kong Chinese Adults: A Controlled Clinical Trial* | *no RCT* |
| *Lera Orsatti, F. P. N., Eliana Aguiar; Nahas-Neto, Jorge; Lera Orsatti, Cláudio; Santos Teixeira, Altamir* | *2013* | *Efeito do treinamento contrarresistência e isoflavona na densidade mineral óssea em mulheres na pós-menopausa* | *language* |
| *Levinger, I. G., C.; Hare, D. L.; Jerums, G.; Selig, S.* | *2007* | *The effect of resistance training on functional capacity and quality of life in individuals with high and low numbers of metabolic risk factors* | *health* |
| *Levinger, I. G., C.; Matthews, V.; Hare, D. L.; Jerums, G.; Garnham, A.; Selig, S.* | *2008* | *BDNF, metabolic risk factors, and resistance training in middle-aged individuals* | *outcome* |
| *Levinger, I. S., S.; Goodman, C.; Jerums, G.; Stewart, A.; Hare, D. L.* | *2011* | *Resistance training improves depressive symptoms in individuals at high risk for type 2 diabetes* | *outcome* |
| *Li, B.* | *2021* | *Effects of Health Qigong in Improving the Cervical and Lumbar Disc Disease and Mental Health Status of Sedentary Young and Middle-Aged Faculties* | *age* |
| *Li, J. X. X., D. Q.; Hong, Y.* | *2009* | *Changes in muscle strength, endurance, and reaction of the lower extremities with Tai Chi intervention* | *age* |
| *Libardi, C. A. D. S., G. V.; Cavaglieri, C. R.; Madruga, V. A.; Chacon-Mikahil, M. P. T.* | *2012a* | *Effect of Resistance, Endurance, and Concurrent Training on TNF-alpha, IL-6, and CRP* | *included* |
| *Libardi C., Bonganha V, et al.* | *2012b* | *The periodized resistance training promotes similar changes in lipid profile in middle-aged men and women* | *included* |
| *Libardi, C. A. S., G. V.; Gáspari, A. F.; Dos Santos, C. F.; Leite, S. T.; Dias, R.; Frollini, A. B.; Brunelli, D. T.; Cavaglieri, C. R.; Madruga, V. A.; Chacon-Mikahil, M. P.* | *2011* | *Effects of concurrent training on interleukin-6, tumour necrosis factor-alpha and C-reactive protein in middle-aged men* | *Data appears elsewhere* |
| *Lijnen et al.* | *1989* | *Effect of prolonged physical exercise on intra-erythrocyte and plasma potassium* | *age* |
| *Lin, Y.-A.; Chen, L.-H.; Chen, F.-P.; Wong, A.; Hsu, C.-C.; Chen, J.-Y.* | *2021* | *The Effectiveness of a Group Kickboxing Training Program on Sarcopenia and Osteoporosis Parameters in Community-Dwelling Adults Aged 50-85 Years* | *health* |
| *Liphardt et al.* | *2015* | *Bone quality in osteopenic postmenopausal women is notimproved after 12 months of whole-body vibration training* | *included* |
| *Liu, S. Y. Z., X.; Sun, J. B.; Li, Q. N.; Liu, Y.; Lian, Z. Q.; Zhang, D. R.* | *2017* | *The effects of 10-week core strength training on the body balance in the middle-aged men* | *language* |
| *Liu, X. M., Y. D.; Burton, N. W.; Chang, J. H.; Brown, W. J.* | *2011* | *Qi-gong mind-body therapy and diabetes control a randomized controlled trial* | *missing data* |
| *Lixandrão, M. E. D., F.; Chacon-Mikahil, M. P.; Cavaglieri, C. R.; Ugrinowitsch, C.; Bottaro, M.; Vechin, F. C.; Conceição, M. S.; Berton, R.; Libardi, C. A.* | *2016* | *Time Course of Resistance Training-Induced Muscle Hypertrophy in the Elderly* | *age* |
| *Lo, Yi-Pang; Chiang, S.-L.; Lin, C.-H.; Liu, H.-C.; Chiang, L.-C.* | *2021* | *Effects of Individualized Aerobic Exercise Training on Physical Activity and Health-Related Physical Fitness among Middle-Aged and Older Adults with Multimorbidity: A Randomized Controlled Trial* | *age* |
| *Lu, X. H.-C., C. W.; Tsang, W. W.* | *2013* | *Effects of Tai Chi training on arterial compliance and muscle strength in female seniors: a randomized clinical trial* | *age* |
| *Lubans, D. R. M., C. M.; Lubans, N. J.; Lonsdale, C. C.* | *2013* | *Pilot randomized controlled trial: elastic-resistance-training and lifestyle-activity intervention for sedentary older adults* | *age* |
| *Maiorana, A. O. D., G.; Dembo, L.; Goodman, C.; Taylor, R.; Green, D.* | *2001* | *Exercise training, vascular function, and functional capacity in middle-aged subjects* | *outcome* |
| *Marcus, R. L. L., P. C.; Dibble, L. E.; Hill, L.; McClain, D. A.* | *2009* | *Increased strength and physical performance with eccentric training in women with impaired glucose tolerance: a pilot study* | *included* |
| *Marston, K. J. P., J. J.; Rainey-Smith, S. R.; Gordon, N.; Teo, S. Y.; Laws, S. M.; Sohrabi, H. R.; Martins, R. N.; Brown, B. M.* | *2019* | *Resistance training enhances delayed memory in healthy middle-aged and older adults: A randomised controlled trial* | *included* |
| *Matsugaki, R. S., M.; Itoh, H.; Matsushima, Y.; Saeki, S.* | *2019* | *Effects of a Physical Therapist Led Workplace Personal-Fitness Management Program for Manufacturing Industry Workers: A Randomized Controlled Trial* | *intervention* |
| *Maynar, M. R., M. C.; Muñoz, D.; Álvarez, J. L.; Grijota, F. J.; Caballero, M. J.* | *2015* | *Effects of a physical activity program on the urinary collagen crosslinks in pre- and postmenopausal women* | *outcome* |
| *Mazini Filho, M. L. A., Felipe J.; Gama de Matos, Dihogo; Costa Moreira, Osvaldo; PatrocÍNio de Oliveira, Cláudia E.; Rezen De de Oliveira Venturini, Gabriela; Magalhaes Curty, Victor; Menezes Touguinha, Henrique; Caputo Ferreira, Maria E.* | *2018* | *Circuit strength training improves muscle strength, functional performance and anthropometric indicators in sedentary elderly women* | *age* |
| *McElroy, J. A. G., T.; Hair, E. C.; Mathews, K. J.; Redman, S. D.; Williams, A.* | *2016* | *Obese But Fit: The Relationship of Fitness to Metabolically Healthy But Obese Status among Sexual Minority Women* | *no RCT* |
| *Megakli, T. V., S. P.; Thogersen-Ntoumani, C.; Theodorakis, Y.* | *2017* | *Impact of aerobic and resistance exercise combination on physical self-perceptions and self-esteem in women with obesity with one-year follow-up* | *age* |
| *Mesquita, L. S. d. C., F. T.; Freire, L. S.; Neto, O. P.; Zângaro, R. A.* | *2015* | *Effects of two exercise protocols on postural balance of elderly women: a randomized controlled trial* | *age* |
| *Mikhael, M. O., Rhonda; Amsen, Fleur; Greene, David; Singh, Maria A. Fiatarone* | *2010* | *Effect of standing posture during whole body vibration training on muscle morphology and function in older adults: a randomised controlled trial* | *age* |
| *Minges, K. E. C., G.; Unglik, E.; Dunstan, D. W.* | *2011* | *Evaluation of a resistance training program for adults with or at risk of developing diabetes: an effectiveness study in a community setting* | *age* |
| *Miyatake, N. T., K.; Wada, J.; Nishikawa, H.; Morishita, A.; Suzuki, H.; Kunitomi, M.; Makino, H.; Kira, S.; Fujii, M.* | *2003* | *Daily exercise lowers blood pressure and reduces visceral adipose tissue areas in overweight Japanese men* | *no RCT* |
| *Mohammadi et al.* | *2018* | *Effects of Different Modes of Exercise Training on Body Composition and Risk Factors for Cardiovascular Disease in Middle-aged Men* | *outcome* |
| *Moitinho-Silva, et al.* | *2021* | *Short-term physical exercise impacts on the human holobiont obtained by a randomised intervention study* | *No RCT* |
| *Monninkhof, E. M. P., P. H.; Schuit, A. J.* | *2007* | *Design of the sex hormones and physical exercise (SHAPE) study* | *outcome* |
| *Moreira, L. F., F. C.; dos Santos, R. N.; Teixeira, L. R.; Kruel, L. F.; Lazaretti-Castro, M.* | *2013* | *High-intensity aquatic exercises (HydrOS) improve physical function and reduce falls among postmenopausal women* | *age* |
| *Moreira, O. C. L., G. S.; de Matos, D. G.; Mazini-Filho, M. L.; Aidar, F. J.; Silva, S. F.; de Oliveira, C. E.* | *2018* | *Impact of two hydrogymnastics class methodologies on the functional capacity and flexibility of elderly women* | *age* |
| *Moreno-* *Muñoz, M.; Hita-Contreras, F.; Estudillo-Martínez, M.; Aibar-Almazán, A.; Castellote-Caballero, Y.; Bergamin, M.; Gobbo, S.; Cruz-Díaz, D.* | *2021* | *The Effects of Abdominal Hypopressive Training on Postural Control and Deep Trunk Muscle Activation: A Randomized Controlled Trial* | *included* |
| *Morganti, C. M. N., M. E.; Fiatarone, M. A.; Dallal, G. E.; Economos, C. D.; Crawford, B. M.; Evans, W. J.* | *1995* | *Strength improvements with 1 yr of progressive resistance training in older women* | *Data appears elsewhere* |
| *Moro, T. T., G.; Bianco, A.; Gottardi, A.; Gottardi, G. B.; Faggian, D.; Plebani, M.; Marcolin, G.; Paoli, A.* | *2017* | *High intensity interval resistance training (HIIRT) in older adults: Effects on body composition, strength, anabolic hormones and blood lipids* | *age* |
| *Mortell, R.; Tucker, L.* | *1993* | *Effects of a 12-week resistive training program in the home using the body bar on dynamic and absolute strength of middle-age women* | *control* |
| *Mulla, D. M. W., E. G.; Chopp-Hurley, J. N.; Kaip, L.; Jarvis, R. S.; Stephens, A.; Keir, P. J.; Maly, M. R.* | *2018* | *The Effects of Lower Extremity Strengthening Delivered in the Workplace on Physical Function and Work-Related Outcomes Among Desk-Based Workers: A Randomized Controlled Trial* | *age* |
| *Müller, A. M. K., S.; Morris, T.* |  | *Text Messaging for Exercise Promotion in Older Adults From an Upper-Middle-Income Country: Randomized Controlled Trial* | *age* |
| *Munn, J. H., R. D.; Hancock, M. J.; evia, S. C.* | *2005* | *Resistance training for strength: effect of number of sets and contraction speed* | *age* |
| *Musanti, R.* | *2012* | *A study of exercise modality and physical self-esteem in breast cancer survivors* | *health* |
| *Nassif, H. B., N.; Guillaume, M.; Delore-Milles, E.; Tafflet, M.; Buchholz, F.; Toussaint, J. F.* | *2011* | *Evaluation of a randomized controlled trial in the management of chronic lower back pain in a French automotive industry: an observational study* | *outcome* |
| *Nct* | *2002* | *Strength Training for Obesity Prevention* | *no fulltext* |
| *Nct* | *2006* | *Physical Activity and Breast Cancer Risk in Postmenopausal Women: the SHAPE Study* | *no fulltext* |
| *Nct* | *2019* | *Effect of a Multi-component Training Program on the Elderly* | *no fulltext* |
| *Nct* | *2020* | *Plyometric, Proprioceptive and Strength Exercises in Rugby Players* | *no fulltext* |
| *Nelson, M. E. F., M. A.; Morganti, C. M.; Trice, I.; Greenberg, R. A.; Evans, W. J.* | *2015* | *Effects of high-intensity strength training on multiple risk factors for osteoporotic fractures. A randomized controlled trial* | *included* |
| *Neves, L. M. F., A. C.; Rossi, F. E.; Diniz, T. A.; Codogno, J. S.; Gobbo, L. A.; Gobbi, S.; Freitas, I. F., Jr.* | *2015* | *Functional training reduces body fat and improves functional fitness and cholesterol levels in postmenopausal women: a randomized clinical trial* | *included* |
| *Nguyen, M. H. K., A.* | *2012* | *A randomized controlled trial of Tai chi for balance, sleep quality and cognitive performance in elderly Vietnamese* | *age* |
| *Nicholson, V. P. M., M. R.; Slater, G. J.; Kerr, A.; Burkett, B. J.* | *2015* | *Low-Load Very High-Repetition Resistance Training Attenuates Bone Loss at the Lumbar Spine in Active Post-menopausal Women* | *age* |
| *Nielsen, T.-T.;* [*Møller*](https://www.tandfonline.com/author/M%C3%B8ller%2C+Trine+K)*, T.; Olesen, N.; Zebis, M.; Ritz, C.; Nordsborg, N.; Hansen, P.; Krustrup, P.* | *2022* | *Improved metabolic fitness, but no cardiovascular health effects, of a low-frequency short-term combined exercise programme in 50-70-year-olds with low fitness: A randomized controlled trial* | *age* |
| *Nikseresht, M.* | *2018* | *Comparison of Serum Cytokine Levels in Men Who are Obese or Men Who are Lean: Effects of Nonlinear Periodized Resistance Training and Obesity* | *age* |
| *Norris, R. C., D.; Cochrane, R.* | *1990* | *The effects of aerobic and anaerobic training on fitness, blood pressure, and psychological stress and well-being* | *outcome* |
| *Nunes, P. R. B., L. C.; Oliveira, A. A.; Furlanetto Júnior, R.; Martins, F. M.; Orsatti, C. L.; Resende, E. A.; Orsatti, F. L.* | *2016* | *Effect of resistance training on muscular strength and indicators of abdominal adiposity, metabolic risk, and inflammation in postmenopausal women: controlled and randomized clinical trial of efficacy of training volume* | *age* |
| *Nunes, P. R. P. B., L. C.; Oliveira, A. A.; Furlanetto, R., Jr.; Martins, F. M.; Resende, Eamr; Orsatti, F. L.* | *2019* | *Muscular Strength Adaptations and Hormonal Responses After Two Different Multiple-Set Protocols of Resistance Training in Postmenopausal Women* | *Data appears elsewhere* |
| *Nunes, P. R. P. O., A. A.; Martins, F. M.; Souza, A. P.; Orsatti, F. L.* | *2017* | *Effect of resistance training volume on walking speed performance in postmenopausal women: A randomized controlled trial* | *included* |
| *Okubo, Y. S., Daina L.; Brodie, Matthew A.; Duran, Lionne; Lord, Stephen R.* | *2019* | *Effect of Reactive Balance Training Involving Repeated Slips and Trips on Balance Recovery Among Older Adults: A Blinded Randomized Controlled Trial* | *age* |
| *Orsatti, F. L. N., E. A.; Maesta, N.; Nahas-Neto, J.; Burini, R. C.* | *2008* | *Plasma hormones, muscle mass and strength in resistance-trained postmenopausal women* | *age* |
| *Osti, F. R. d. S., C. R.; Teixeira, L. A.* | *2018* | *Improvement of Balance Stability in Older Individuals by On-Water Training* | *age* |
| *Palumbo, M.; Wu, G.; Shaner-McRae, H.; Rambur, B.; McIntosh, B.* | *2012* | *Tai Chi for older nurses: A workplay wellness pilot study* | *included* |
| *Paoli, A. P., F.; Bargossi, A. M.; Marcolin, G.; Guzzinati, S.; Neri, M.; Bianco, A.; Palma, A.* | *2010* | *Effects of three distinct protocols of fitness training on body composition, strength and blood lactate* | *included* |
| *Paolillo, F. R. C., A. V.; Paolillo, A. R.; Borghi-Silva, A.; Arena, R.; Kurachi, C.; Bagnato, V. S.* | *2014* | *Phototherapy during treadmill training improves quadriceps performance in postmenopausal women* | *intervention* |
| *Park, M. S.; Kim, K. S.* | *2014* | *Effects of Yoga Exercise Program on Response of Stress, Physical Fitness and Self-esteem in the Middle-aged Women* | *language* |
| *Park et al.* | *2015* | *The Effects of Combined Exercise on Health-Related Fitness,Endotoxin, and Immune Function of Postmenopausal Womenwith Abdominal Obesity* | *included* |
| *Parra, R.; Karabulut, M.* | *2021* | *Comparing Neuromuscular Adaptations In Middle-aged Males Following 8 Weeks Of Blood Flow Restriction Training* | *No fulltext* |
| *Pareja-Blanco, F. A.-C., J. A.; Sáez de Villarreal, E.* | *2021* | *Combined Squat and Light-Load Resisted Sprint Training for Improving Athletic Performance* | *age* |
| *Patti, A. B., A.; Karsten, B.; Montalto, M. A.; Battaglia, G.; Bellafiore, M.; Cassata, D.; Scoppa, F.; Paoli, A.; Iovane, A.; Messina, G.; Palma, A.* | *2017* | *The effects of physical training without equipment on pain perception and balance in the elderly: A randomized controlled trial* | *age* |
| *Peake, J. M. K., S.; Nowson, C. A.; ers, K.; Daly, R. M.* | *2011* | *Inflammatory cytokine responses to progressive resistance training and supplementation with fortified milk in men aged 50+ years: an 18-month randomized controlled trial* | *age* |
| *Pellecchia et al.* | *2005* | *Dual-Task Training Reduces Impact of CognitiveTask on Postural Sway* | *age* |
| *Perchthaler, D. G., S.; Hein, T.* | *2015* | *Evaluation of a six-week whole-body vibration intervention on neuromuscular performance in older adults* | *included* |
| *Pereira de Paiva, E. B. L., Fabiano; Mendonça Marinho, Samantha* | *2019* | *Dança de salão na prevenção de quedas em idosos: estudo caso controle* | *age* |
| *Peres Campos, A. L. r. d. S. D. P., Lourenço; Schüler Cavalli, Adriana; da Rosa Afonso, Mariângela; Gomes Schild, José Franscisco; Fossati Reichert, Felipe* | *2013* | *Effects of concurrent training on health aspects of elderly women* | *age* |
| *Pérez-Idárraga, A. r. V. G., Katerine; Gallo Villegas, Jaime; Arenas Sosa, Mónica; Quintero Velásquez, Mario A.* | *2015* | *Intervención con rumba y educación nutricional para modificar factores de riesgo cardiovascular en adultos con síndrome metabólico* | *language* |
| *Petkova, V.; Aleksandrova, V.* | *2022* | *Aquatic Physical Activity as a Health Prevention in 55-60-Year-Old Women with Early Osteoporosis* | *no fulltext* |
| *Piacentini, M. F. D. I., G.; Comotto, S.; Spedicato, A.; Vernillo, G.; La Torre, A.* | *2013* | *Concurrent strength and endurance training effects on running economy in master endurance runners* | *master runners* |
| *Pincivero, D. M. C., R. M.* | *2004* | *The effects of rest interval length and training on quadriceps femoris muscle. Part I: knee extensor torque and muscle fatigue* | *age* |
| *Pirauá, A.; de Oliveira, V.; Cavalcante, B.; Beltrão, N.; Batista, G.; Pitangui, A.; Araújo, R.* | *2021* | *Effects of 24 weeks strength training with and without unstable devices on strength, flexibility and quality of life in older women: A secondary analysis from randomized controlled trial* | *No fulltext* |
| *Pirouzi, S. M., Ali Reza; Fallahzadeh, Fatemeh; Fallahzadeh, Mohammad Amin* | *2014* | *Effectiveness of Treadmill Training on Balance Control in Elderly People: A Randomized Controlled Clinical Trial* | *age* |
| *Porter, M. M. N., M. E.; Singh, M. A. F.; Layne, J. E.; Morganti, C. M.; Trice, I.; Economos, C. D.; Roubenoff, R.; Evans, W. J.* | *2002* | *Effects of long-term resistance training and detraining on strength and physical activity in older women* | *no RCT* |
| *Potier, T. G. A. e., C. M.; Seynnes, O. R.* | *2009* | *Effects of eccentric strength training on biceps femoris muscle architecture and knee joint range of movement* | *age* |
| *Prieto Saborit, J. A. N. H. e., P.; Ruiz Fern; ez, L.; Del Valle Soto, M.* | *2014* | *The influence of intervention methodology on the physical condition of sedentary older adults* | *no fulltext* |
| *Pullyblank, K. S., D.; Folta, S. C.; Paul, L.; Nelson, M. E.; Graham, M.; Marshall, G. A.; Eldridge, G.; Parry, S. A.; Mebust, S.; Seguin, R. A.* | *2020* | *Effects of the Strong Hearts, Healthy Communities Intervention on Functional Fitness of Rural Women* | *age* |
| *Purath, J. K., Colleen S.; McPherson, Sterling; Ainsworth, Barbara* | *2013* | *A randomized controlled trial of an office-based physical activity and physical fitness intervention for older adults* | *age* |
| *Ramirez-Campillo, R. D., D.; Martinez-Salazar, C.; Valdés-Badilla, P.; Delgado-Floody, P.; Méndez-Rebolledo, G.; Cañas-Jamet, R.; Cristi-Montero, C.; García-Hermoso, A.; Celis-Morales, C.; Moran, J.; Buford, T. W.; Rodriguez-Mañas, L.; Alonso-Martinez, A. M.; Izquierdo, M.* | *2016* | *Effects of different doses of high-speed resistance training on physical performance and quality of life in older women: a randomized controlled trial* | *age* |
| *Rathod, S. S., Nehal* | *2015* | *The Effect of Training Core Stabilizers in Clerks with Low Back Pain* | *control* |
| *Razian, M.; Hosseinzadeh, M.; Behm, D.; Sardroodian, M.* | *2020* | *Effect of leg dominance on ipsilateral and contralateral limb training adaptation in middle-aged women after unilateral sensorimotor and resistance exercise training* | *No fulltext* |
| *Reis, J. G. C., G. C.; Schmidt, A.; Ferreira, C. H.; Abreu, D. C.* | *2012* | *Do muscle strengthening exercises improve performance in the 6-minute walk test in postmenopausal women?* | *included* |
| *Rendon, A. A. L., E. B.; Thorpe, D.; Johnson, E. G.; Medina, E.; Bradley, B.* | *2012* | *The effect of virtual reality gaming on dynamic balance in older adults* | *age* |
| *Rockette-Wagner, B.; Miller, R.; Eaglehouse, Y.; Arena, V.; Kramer, M.; Kriska, A.* | *2020* | *Leisure Sedentary Behavior Levels and Meeting Program Goals in a Community Lifestyle Intervention for Diabetes Prevention* | *No fulltext* |
| *Rodrigues, B. G. S. C., S. A.; Torres, N. V. O.; de Oliveira, E. M.; Dantas, E. H. M.* | *2010* | *Pilates method in personal autonomy, static balance and quality of life of elderly females* | *age* |
| *Roelants, M. D., C.; Verschueren, S. M.* | *2004* | *Whole-body-vibration training increases knee-extension strength and speed of movement in older women* | *age* |
| *Rogers, C. E. K., C.; Larkey, L. K.; Ainsworth, B. E.* | *2012* | *A randomized controlled trial to determine the efficacy of Sign Chi Do exercise on adaptation to aging* | *age* |
| *Rogge, A. K. R., B.; Zech, A.; Hötting, K.* | *2018* | *Exercise-induced neuroplasticity: Balance training increases cortical thickness in visual and vestibular cortical regions* | *age* |
| *Rogge, A. K. R., B.; Zech, A.; Nagel, V.; Holl; er, K.; Braumann, K. M.; Hötting, K.* | *2017* | *Balance training improves memory and spatial cognition in healthy adults* | *age* |
| *Rossi, L. P. B. a., Michelle; Pereira, Rafael; Silveira Gomes, Anna Raquel* | *2014* | *The Effects of a Perturbation-Based Balance Training on Neuromuscular Recruitment and Functional Mobility in Community-Dwelling Older Women* | *age* |
| *Rudolfsson, T. D., M.; Häger, C.; Björklund, M.* | *2014* | *Effects of neck coordination exercise on sensorimotor function in chronic neck pain: a randomized controlled trial* | *health* |
| *Rustaden, A. M. H., L. A. H.; Paulsen, G.; Bø, K.* | *2017* | *Effects of BodyPump and resistance training with and without a personal trainer on muscle strength and body composition in overweight and obese women-A randomised controlled trial* | *age* |
| *Ryan et al.* | *1985* | *Effects of strength training on bone mineral density: hormonal and bone turnover relationships* | *age* |
| *rz4yq, R. B. R.* | *2015* | *Effects of two bodybuilding programs in health and physical performance* | *no fulltext* |
| *Sales, M. P., R.; Hill, K. D.; Levinger, P.* | *2017* | *A Novel Exercise Initiative for Seniors to Improve Balance and Physical Function* | *age* |
| *Sallinen J, Fogelholm M, Volek J, Kraemer W, Alen M, Häkkinen K* | *2007* | *Effects of Strength Training and Reduced Trainingon Functional Performance and Metabolic Health Indicators in Middle-Aged Men* | *included* |
| *Sañudo, B. G.-N., Á; Álvarez-Barbosa, F.; de Hoyo, M.; Del Pozo, J.; Rogers, M. E.* | *2019* | *Effect of Flywheel Resistance Training on Balance Performance in Older Adults. A Randomized Controlled Trial* | *age* |
| *Saucedo Rodrigo, P. A. A., J.; Gómez Jara, P.; Leal Hernández, M.; Ortega Toro, E.; Colado, J. C.; Sáinz de Bar; a Andújar, P.* | *2008* | *Effects of a structured exercise programme on cardiovascular risk programmes in post-menopausal women. CLIDERICA study* | *outcome* |
| *Scharhag-Rosenberger, F. M., T.; Wegmann, M.; Ruppenthal, S.; Kaestner, L.; Morsch, A.; Hecksteden, A.* | *2014* | *Irisin does not mediate resistance training-induced alterations in resting metabolic rate* | *outcome* |
| *Schlenstedt, C. A., M.; Mancini, M.; Deuschl, G.; Weisser, B.* | *2017* | *The effect of unilateral balance training on postural control of the contralateral limb* | *age* |
| *Schmitz, K. H. A., R. L.; Yee, D.* | *2002* | *Effects of a 9-month strength training intervention on insulin, insulin-like growth factor (IGF)-I, IGF-binding protein (IGFBP)-1, and IGFBP-3 in 30-50-year-old women* | *outcome* |
| *Schmitz, K. H. J., M. D.; Kugler, K. C.; Jeffery, R. W.; Leon, A. S.* | *2003* | *Strength training for obesity prevention in midlife women* | *missing data* |
| *Scholz, U. B., C.* | *2014* | *A Dyadic Action Control Trial in Overweight and Obese Couples (DYACTIC)* | *outcome* |
| *Schroeder, E. C. F., W. D.; Sharp, R. L.; Lee, D. C.* | *2019* | *Comparative effectiveness of aerobic, resistance, and combined training on cardiovascular disease risk factors: A randomized controlled trial* | *age* |
| *Seguin-Fowler, R. A. S., David; Graham, Meredith L.; Eldridge, Galen D.; Marshall, Grace A.; Folta, Sara C.; Pullyblank, Kristin; Nelson, Miriam E.; Paul, Lynn* | *2020* | *The Strong Hearts, Healthy Communities Program 2.0: An RCT Examining Effects on Simple 7* | *age* |
| *Sellami, M.; Ben Abderrahmen, A.; Dhahbi, W.; Hayes, L.D.; Zouhal, H.* | *2021* | *Hemoglobin, hematocrit and plasma volume variations following combined sprint and strength: Effect of advanced age* | *outcome* |
| *Seguin-Fowler, R.; Eldridge, G.; Rethorst, C.; Graham, M.; Demment, M.; Strogatz, D.; Folta, S.; Maddock, J.; Nelson, M.; Ha, S.* | *2022* | *Improvements and Maintenance of Clinical and Functional Measures Among Rural Women: Strong Hearts, Healthy Communities-2. 0 Cluster Randomized Trial* | *age* |
| *Sen, E. I. E., S.; Eskiyurt, N.* | *2020* | *Effects of whole-body vibration and high impact exercises on the bone metabolism and functional mobility in postmenopausal women* | *health* |
| *Sforzo, G. A. M., B. G.; Black, D.; Luniewski, D.; Scriber, K. C.* | *1995* | *Resilience to exercise detraining in healthy older adults* | *age* |
| *Shaw, B. S. G., M.; McIntyre, S.; Shaw, I.* | *2016* | *Anthropometric and cardiovascular responses to hypertrophic resistance training in postmenopausal women* | *included* |
| *Shirazi, K. K. W., L. M.; Niknami, S.; Hidarnia, A.; Torkaman, G.; Gilchrist, M.; Faghihzadeh, S.* | *2007* | *A home-based, transtheoretical change model designed strength training intervention to increase exercise to prevent osteoporosis in Iranian women aged 40-65 years: a randomized controlled trial* | *included* |
| *Sillanpää, E. H., A.; Nyman, K.; Mattila, M.; Cheng, S.; Karavirta, L.; Laaksonen, D. E.; Huuhka, N.; Kraemer, W. J.; Häkkinen, K.* | *2008* | *Body composition and fitness during strength and/or endurance training in older men* | *missing data* |
| *Sillanpää, E. H., A.; Punnonen, K.; Häkkinen, K.; Laaksonen, D. E.* | *2009* | *Effects of strength and endurance training on metabolic risk factors in healthy 40-65-year-old men* | *missing data* |
| *Sillanpää, E. H., A.; Laaksonen, D. E.; Karavirta, L.; Kraemer, W. J.; Häkkinen, K.* | *2010* | *Serum basal hormone concentrations, nutrition and physical fitness during strength and/or endurance training in 39 -- 64-year-old women* | *missing data* |
| *Sillanpää, E. L., D. E.; Häkkinen, A.; Karavirta, L.; Jensen, B.; Kraemer, W. J.; Nyman, K.; Häkkinen, K.* | *2009* | *Body composition, fitness, and metabolic health during strength and endurance training and their combination in middle-aged and older women* | *missing data* |
| *Sinaki, M. C., J. C.; Phillips, B. E.; Clarke, B. L.* | *2004* | *Site specificity of regular health club exercise on muscle strength, fitness, and bone density in women aged 29 to 45 years* | *age* |
| *Singh, J. A. S., K. H.; Petit, M. A.* | *2009* | *Effect of resistance exercise on bone mineral density in premenopausal women* | *included* |
| *Sipilä, S. T., D. R.; Cheng, S.; Puolakka, J.; Toivanen, J.; Suominen, H.* | *2001* | *Effects of hormone replacement therapy and high-impact physical exercise on skeletal muscle in post-menopausal women: a randomized placebo-controlled study* | *included* |
| *Siriphorn, A. S., Siriporn Vongsaiyat; Sawatthuk, Kittaphon; Temvorasub, Kanjana; Auttawut, Malinee* | *2019* | *Exercise using a foam bead bag improves balance and lower extremity strength in older adults...38th Scientific Meeting of the Physiotherapy Research Society, April 26, 2019, London South Bank University, UK* | *age* |
| *Siu, P.; Yu, A.; Chin, E.; Yu, D.; Hui, S.; Woo, J.; Gonf, D.; Wei, C.; Irwin, M.* | *2021* | *Effects of Tai Chi or Conventional Exercise on Central Obesity in Middle-Aged and Older Adults : A Three-Group Randomized Controlled Trial* | *age* |
| *Skargren, E. O., B.* | *1996* | *Effects of an exercise program on musculoskeletal symptoms and physical capacity among nursing staff* | *age* |
| *Smith, M. F. E., Mistrelle; Middleton, Geoff; Mur-Gatroyd, Paul M.; Gee, Thomas I.* | *2017* | *Effects of Resistance Band Exercise on Vascular Activity and Fitness in Older Adults* | *included* |
| *Soori et al.* | *2017* | *Effects of regular physical activity on levels of nesfatin-1, neuropeptideY and cortisol in obese men* | *language* |
| *Souza, D. B., M.; Vieira, C. A.; Martins, W. R.; Cadore, E. L.; Gentil, P.* | *2019* | *Minimal dose resistance training with elastic tubes promotes functional and cardiovascular benefits to older women* | *age* |
| *Souza, G. V. L., C. A.; Rocha Junior, J.; Madruga, V. A.; Chacon-Mikahil, M. P.* | *2012* | *Effect of concurrent training on components of the metabolic syndrome in middle-aged men* | *language* |
| *Sparrow, D. G., D. J.; Demolles, D.; Fielding, R. A.* | *2011* | *Increases in muscle strength and balance using a resistance training program administered via a telecommunications system in older adults* | *age* |
| *Speck, R. M. G., C. R.; Hormes, J. M.; Ahmed, R. L.; Lytle, L. A.; Hwang, W. T.; Schmitz, K. H.* | *2010* | *Changes in the Body Image and Relationship Scale following a one-year strength training trial for breast cancer survivors with or at risk for lymphedema* | *health* |
| *Spennewyn, K. C.* | *2008* | *Strength outcomes in fixed versus free-form resistance equipment* | *control* |
| *Spiliopoulou, S. I. A., I. G.; Tsigganos, G.; Economides, D.; Kellis, E.* | *2010* | *Vibration effects on static balance and strength* | *missing data* |
| *Stania, M. K., P.; Sobota, G.; Polak, A.; Bacik, B.; Juras, G.* | *2017* | *The effect of the training with the different combinations of frequency and peak-to-peak vibration displacement of whole-body vibration on the strength of knee flexors and extensors* | *age* |
| *Steinberg, M; Cartwright, C.; Peel, N.; Williams, G.* | *2000* | *A sustainable programme to prevent falls and near falls in community dwelling older people: results of a randomised trial* | *age* |
| *Stensvold, D. T., A. E.; Skaug, E. A.; Aspenes, S.; Stølen, T.; Wisløff, U.; Slørdahl, S. A.* | *2010* | *Strength training versus aerobic interval training to modify risk factors of metabolic syndrome* | *health* |
| *Stewart, K. J. B., A. C.; Hees, P. S.; Tayback, M.; Ouyang, P.; Jan de Beur, S.* | *2005* | *Exercise effects on bone mineral density relationships to changes in fitness and fatness* | *age* |
| *Stewart, K. J. B., A. C.; Turner, K.; Lim, J. G.; Hees, P. S.; Shapiro, E. P.; Tayback, M.; Ouyang, P.* | *2005* | *Exercise and risk factors associated with metabolic syndrome in older adults* | *age* |
| *Stewart, K. J. B., A. C.; Turner, K. L.; Fleg, J. L.; Hees, P. S.; Shapiro, E. P.; Tayback, M.; Ouyang, P.* | *2005* | *Effect of exercise on blood pressure in older persons: a randomized controlled trial* | *age* |
| *Sukkeaw et al.* | *2017* | *A Comparison between the Effects of Aerobic DanceTraining on Mini-Trampoline and Hard Wooden Surfaceon Bone Resorption, Health-Related Physical Fitness,Balance, and Foot Plantar Pressure in Thai WorkingWomen* | *no RCT* |
| *Suksom, D. P., Y.; Soogarun, S.; Sapwarobol, S.* | *2014* | *Step aerobic combined with resistance training improves cutaneous microvascular reactivity in overweight women* | *no fulltext* |
| *Sun et al.* | *2005* | *Effect of shadow boxing on the physical quality of middle-aged and old women* | *no fulltext* |
| *Suttanon, P. P., Pagamas; Krootnark, Kitsana; Aranyavalai, Thanyaporn* | *2018* | *Effectiveness of falls prevention intervention programme in community-dwelling older people in Thailand: Randomized controlled trial* | *age* |
| *Taaffe, D. R. S., S.; Cheng, S.; Puolakka, J.; Toivanen, J.; Suominen, H.* | *2005* | *The effect of hormone replacement therapy and/or exercise on skeletal muscle attenuation in postmenopausal women: a yearlong intervention* | *included* |
| *Taati, B.; Arazi, H.; Kheirkhah, J.* | *2021* | *Interaction effect of green tea consumption and resistance training on office and ambulatory cardiovascular parameters in women with high-normal/stage 1 hypertension* | *outcome* |
| *Taddei, U. T. M., A. B.; Ribeiro, F. I. A.; Bus, S. A.; Sacco, I. C. N.* | *2020* | *Effects of a foot strengthening program on foot muscle morphology and running mechanics: A proof-of-concept, single-blind randomized controlled trial* | *included* |
| *Taddei, U. T. M., A. B.; Ribeiro, F. I. A.; Inoue, R. S.; Bus, S. A.; Sacco, I. C. N.* | *2018* | *Effects of a therapeutic foot exercise program on injury incidence, foot functionality and biomechanics in long-distance runners: Feasibility study for a randomized controlled trial* | *no RCT* |
| *Takeshima, N., Mohammod M.; Rogers, Michael E.; Rogers, Nicole L.; Naoko, Sengoku; Daisuke, Koizumi; Yukiko, Kitabayashi; Aiko, Imai; Aiko, Naruse* | *2013* | *Effects of Nordic Walking compared to Conventional Walking and Band-Based Resistance Exercise on Fitness in Older Adults* | *age* |
| *Teixeira de Carvalho, F. d. A. M., L. S.; Pereira, R.; Neto, O. P.; Amaro Zangaro, R.* | *2017* | *Pilates and Proprioceptive Neuromuscular Facilitation Methods Induce Similar Strength Gains but Different Neuromuscular Adaptations in Elderly Women* | *age* |
| *Teixeira, P. J. G., S. B.; Houtkooper, L. B.; Metcalfe, L. L.; Blew, R. M.; Flint-Wagner, H. G.; Cussler, E. C.; Sardinha, L. B.; Lohman, T. G.* | *2003* | *Resistance training in postmenopausal women with and without hormone therapy* | *outcome* |
| *Teoman, N. O., A.; Acar, B.* | *2004* | *The effect of exercise on physical fitness and quality of life in postmenopausal women* | *health* |
| *Tesch, P. A. T., J. T.; Ekberg, A.* | *2004* | *Hypertrophy of chronically unloaded muscle subjected to resistance exercise* | *age* |
| *Thomas, K. J. T., J. B.; Martin, M. S.* | *2004* | *Does participation in light to moderate strength and endurance exercise result in measurable physical benefits for older adults?* | *age* |
| *Thomas, M. K., Michael* | *2016* | *The Effects of Slackline Balance Training on Postural Control in Older Adults* | *age* |
| *Tomeleri, C.; Ribeiro, A.; Nunes, J.; Schoenfeld, B.; Souza, M.; Schiavoni, D.; Junior, P.; Cavaglieri, C.; Cunha, P.; Venturini, D.; Barbosa, D.; Cyrino, E.* | *2020* | *Influence of Resistance Training Exercise Order on Muscle Strength, Hypertrophy, and Anabolic Hormones in Older Women: A Randomized Controlled Trial* | *age* |
| *Tsaih, P. L. S., Y. L.; Hu, M. H.* | *2012* | *Low-intensity task-oriented exercise for ambulation-challenged residents in long-term care facilities: a randomized, controlled trial* | *age* |
| *Tsourlou, T. G., V.; Kellis, E.; Stavropoulos, N.; Kellis, S.* | *2003* | *The effects of a calisthenics and a light strength training program on lower limb muscle strength and body composition in mature women* | *included* |
| *Uusi-Rasi K, et al.* | *2003* | *Effect of alendronate and exercise on bone and physical performance of postmenopausal women: a randomized controlled trial* | *included* |
| *Uritani, D.; Matsumoto,D.; Asano, Y.; Yoshizaki, K.; Nishida, Y.; Shima, M.* | *2013* | *Effects of regular exercise and nutritional guidance on body composition, blood pressure, muscle strength and health-related quality of life in community-dwelling Japanese women* | *age* |
| *Huang, T, Yang; Chia-Yih, Liu* | *2011* | *Reducing the fear of falling among community-dwelling elderly adults through cognitive-behavioural strategies and intense Tai Chi exercise: a randomized controlled trial* | *age* |
| *Van Roie, E. D., C.; Opdenacker, J.; De Bock, K.; Kennis, E.; Boen, F.* | *2010* | *Effectiveness of a lifestyle physical activity versus a structured exercise intervention in older adults* | *age* |
| *Vannozzi, G. P., F.; Caserotti, P.; Cappozzo, A.* | *2008* | *A neurofuzzy inference system based on biomechanical features for the evaluation of the effects of physical training* | *age* |
| *Velthuis, M. J. S., A. J.; Peeters, P. H.; Monninkhof, E. M.* | *2009* | *Exercise program affects body composition but not weight in postmenopausal women* | *outcome* |
| *Verhagen, E. v. d. B., A.; Twisk, J.; Bouter, L.; Bahr, R.; van Mechelen, W.* | *2004* | *The effect of a proprioceptive balance board training program for the prevention of ankle sprains: a prospective controlled trial* | *age* |
| *Verschueren, S. M. R., M.; Delecluse, C.; Swinnen, S.; erschueren, D.; Boonen, S.* | *2004* | *Effect of 6-month whole body vibration training on hip density, muscle strength, and postural control in postmenopausal women: a randomized controlled pilot study* | *age* |
| *Vojciechowski, A. S. B., Simone; Melo Filho, Jarbas; Rabito, Estela Iraci; Amaral, Maryelle Paula do; Gomes, Anna Raquel Silveira* | *2018* | *Effects of physical training with the Nintendo Wii Fit Plus® and protein supplementation on musculoskeletal function and the risk of falls in pre-frail older women: Protocol for a randomized controlled clinical trial (the WiiProtein study)* | *age* |
| *Vorup, J. T., Jonas; Gunnarsson, Thomas; Ravnholt, Tanja; Dalsgaard, Sarah; Bangsbo, Jens; Gunnarsson, Thomas P.* | *2016* | *Effect of speed endurance and strength training on performance, running economy and muscular adaptations in endurance-trained runners* | *age* |
| *Wagner, P. B., W.; Sygusch, R.* | *2004* | *The seven-sequence intervention: sedentary adults on their way to fitness and health* | *outcome* |
| *Wanderley, F. A. C. O., Nórton Luis; Marques, Elisa; Moreira, Pedro; Oliveira, José; Carvalho, Joana* | *2015* | *Aerobic Versus Resistance Training Effects on Health-Related Quality of Life, Body Composition, and Function of Older Adults* | *age* |
| *Wang, J. T., S.; Cao, L.* | *2015* | *Exercise training at the maximal fat oxidation intensity improved health-related physical fitness in overweight middle-aged women* | *outcome* |
| *Wang, H.; Zhang, T.; Lu, M.; Zeng, Y.; Xiao, Y.; Ren, X.; Zhang, P.* | *2021* | *Effects of Physical Activity and Counselling Interventions on Health Outcomes among Working Women in Shanghai* | *Outcome* |
| *Wharton, W.; Jeong, L.; Ni, L.; Bay, A.; Shin, R.; McCullough, L.; Silverstein, H.; Hart, A.; Swieboda, D.; Hu, W.; Hackney, M.* | *2021* | *A Pilot randomized clinical trial of adapted tango to improve cognition and psychosocial function in African American women with family history of Alzheimer's disease (ACT trial)* | *age* |
| *Wayne, P. M. K., D. P.; Buring, J. E.; Connors, E. M.; Bonato, P.; Yeh, G. Y.; Cohen, C. J.; Mancinelli, C.; Davis, R. B.* | *2012* | *Impact of Tai Chi exercise on multiple fracture-related risk factors in post-menopausal osteopenic women: a pilot pragmatic, randomized trial* | *outcome* |
| *Wei, N. N., Gabriel Y. F.* | *2018* | *The effect of whole body vibration training on quadriceps voluntary activation level of people with age-related muscle loss (sarcopenia): a randomized pilot study* | *age* |
| *Wei, N. P., Marco Y. C.; Ng, Shamay S. M.; Ng, Gabriel Y. F.* | *2017* | *Optimal frequency/time combination of whole-body vibration training for improving muscle size and strength of people with age-related muscle loss (sarcopenia): A randomized controlled trial* | *age* |
| *Wen, H. J. H., T. H.; Li, T. L.; Chong, P. N.; Ang, B. S.* | *2017* | *Effects of short-term step aerobics exercise on bone metabolism and functional fitness in postmenopausal women with low bone mass* | *included* |
| *Westlake, K. P. C., E. G.* | *2007* | *Sensory-specific balance training in older adults: effect on proprioceptive reintegration and cognitive demands* | *age* |
| *Wherry, S. J. A., C. D.; Swan, P. D.* | *2019* | *Feasibility of a Home-Based Balance Intervention in Middle-Aged Women Using Wii Fit Plus (R)* | *no fulltext* |
| *Whiteford, J. A., T. R.; Dhaliwal, S. S.; James, A. P.; Woodhouse, J. J.; Price, R.; Prince, R. L.; Kerr, D. A.* | *2010* | *Effects of a 1-year randomized controlled trial of resistance training on lower limb bone and muscle structure and function in older men* | *age* |
| *Williams et al.* | *1997* | *Effects of group exercise on cognitive functioning and mood in older women* | *age* |
| *Winters, K. M. S., C. M.* | *2000* | *Detraining reverses positive effects of exercise on the musculoskeletal system in premenopausal women* | *age* |
| *Winters-Stone, K. M. D., J.; Bennett, J. A.; Nail, L. M.; Leo, M. C.; Schwartz, A.* | *2012* | *The effect of resistance training on muscle strength and physical function in older, postmenopausal breast cancer survivors: a randomized controlled trial* | *health* |
| *Winters-Stone, K. M. L., F.; Horak, F.; Luoh, S. W.; Bennett, J. A.; Nail, L.; Dieckmann, N.* | *2012* | *Comparison of tai chi vs. strength training for fall prevention among female cancer survivors: study protocol for the GET FIT trial* | *health* |
| *Wittke, A. v. S., S.; Hettchen, M.; Fröhlich, M.; Giessing, J.; Lell, M.; Scharf, M.; Bebenek, M.; Kohl, M.; Kemmler, W.* | *2017* | *Protein Supplementation to Augment the Effects of High Intensity Resistance Training in Untrained Middle-Aged Males: The Randomized Controlled PUSH Trial* | *included* |
| *Wong, A. F., A.; Son, W. M.; Chernykh, O.; Park, S. Y.* | *2018* | *The effects of stair climbing on arterial stiffness, blood pressure, and leg strength in postmenopausal women with stage 2 hypertension* | *included* |
| *x3tkf, R. B. R.* | *2017* | *Physical Activity for men in Andropause* | *no fulltext* |
| *Liu, X, Gao; Bing-xiang, Yin; Xiang-yu, Yang; Ding-xi, Bai* | *2016* | *Efficacy of Ba Duan Jin in Improving Balance* | *age* |
| *Yang, H. J. C., Kuei‐Min; Chen, Ming‐De; Wu, Hui‐Chuan; Chang, Wen‐Jane; Wang, Yueh‐Chin; Huang, Hsin‐Ting* | *2015* | *Applying the transtheoretical model to promote functional fitness of community older adults participating in elastic band exercises* | *age* |
| *Yasuda, T. F., K.; Sato, Y.; Yamasoba, T.; Nakajima, T.* | *2014* | *Effects of detraining after blood flow-restricted low-intensity training on muscle size and strength in older adults* | *age* |
| *Yu, J.-H. L., Gyu-Chang* | *2012* | *Effect of core stability training using pilates on lower extremity muscle strength and postural stability in healthy subjects* | *age* |
| *yxds, R. B. R.* | *2016* | *Effects of exercise training on the human body* | *no fulltext* |
| *Zapata-Lamana, R. C., I.; Díaz, E.; Saavedra, C.; Monsalves, M.* | *2015* | *Efectos del entrenamiento fuerza-resistencia en el control de glucosa y función muscular en mujeres adultas sedentarias de Los Ángeles, Chile* | *language* |
| *Zhao, J. Z., L.; Tian, Y.* | *2007* | *Effect of 6 months of Tai Chi Chuan and calcium supplementation on bone health in females aged 50-59 years* | *included* |
| *Zhou, J. C., Shuwan; Cong, Yan; Qin, Meiqin; Sun, Wei; Lian, Jianhua; Yao, Jian; Li, Weiping; Hong, Youlian* | *2015* | *Effects of 24 weeks of Tai Chi Exercise on Postural Control among Elderly Women* | *age* |
| *Zink-Rückel, C.; Kohl, M.; Willert, S.; von Stengel, S.; Kemmler, W.* | *2021* | *Once-Weekly Whole-Body Electromyostimulation Increases Strength, Stability and Body Composition in Amateur Golfers. A Randomized Controlled Study* | *age* |
| *Zou et al.* | *2019* | *Superior Effects of Modified Chen-Style Tai Chi versus 24-Style Tai Chi on Cognitive Function, Fitness, and Balance Performance in Adults over 55* | *age* |

**Supplement 6: Individual study characteristics**

| **Study authors** | **Study ID** | **N (included sex)** | **Mean age in ears (SD/SE)** | **Body compsition / Physical inactivity** | **Intervention** | **Classification** | **Domain of strength / balance: Test modality** |
| --- | --- | --- | --- | --- | --- | --- | --- |
| Adams et al. (2001) [14] | 1 | 19 (w) | 51.17 (±7.38) | NA / Inactive | Primary exercises: leg press, bench press; Assistance exercises: dumbbell lunge, hamstring curl, dumbbell incline press, dumbbell row, lat pull-down, triceps press-down, and dumbbell biceps curl. | Strength training | Maximum strength: 1-RM test for leg press  Strength-endurance:  Leg press, maximal number of repetitions of 70% of 1-RM  Power:  Modified Wingate bicycle test |
| Almarzouki et al. (2020) [15] | 2 | 52 (w+m) | 54.4 (±5.4) | Overweighted / NA | Balance exercises: standing on one foot, walking heel to toe, heel walking. Strength exercises: side-kicks, wall-squats | Balance / Functional training | Proactive Balance: Y-Balance test |
| Anek & Bunyaratavej (2015) [16] | 4 | 52 (w) | 50.81 (±3.13) | Normal-weighted / NA | Circuit aerobic step exercise | Aerobic | Maximum strength: 1-RM test on a leg dynamometer  Static Balance:  CoP displacement standing on a hard surfaces with eyes open |
| Anek et al. (2015) [17] | 5 | 60 (w) | 40.6 (±3.6) | Normal-weighted / NA | Exp1: Aerobic step exercise  Exp2: Resistance aerobic exercise  Exp3: Combined aerobic step with resistance exercise training | Exp1: Aerobic  Exp2: Aerobic  Exp3: Strength-Aerobic | Maximum strength: Leg extension on a weight machine  Static Balance:  CoP displacement standing on a hard surfaces with eyes open |
| Araújo et al. (2015) [18] | 6 | 28( w) | 53.9 (±3.8) | Overweighted / NA | Exp1: Exercises in a swimming pool for the lower limbs Exp2: Exercises in a swimming pool for the lower limbs combined with blood flow restriction | Waterbased training | Maximum strength:  1-RM test of knee extension on a weight machine  Power:  5-times-sit-to-stand  Proactive Balance: Timed-up-and-go test  Dynamic Balance:  Normal gait speed on a 6 meter walk |
| Asikainen et al. (2006) [19] | 7 | 134 (w) | 57.3 (± 4.3) | NA/Inactive | Exp1: brisk walking in 1 bout and resistance training Exp2: brisk walking in 2 bouts and resistance training | Strength-endurance training | Maximum strength:  One-leg squat test  Static balance:  One-leg standing balance-test |
| Batrakoulis et al. (2022) [20] | 98 | 97 (w+m) | 44.76 (± 5.13) | Obese/Inactive | 1-year hybrid-type, multicomponent interval training programme (DoIT) once weekly (Exp1), twice weekly (Exp2) or thrice weekly (Exp3) | Strength training | Maximum strength: One-repetition maximum, horizontal leg press.  Muscle Power:  Chair Squat Test  Static Balance:  Modified Romberg Test |
| Bemben et al. (2000) [21] | 9 | 25 (w) | 51.5 (±1.77) | Exp1: Overweighted/ NA  Exp2: Normal-weighted / NA | Exp1: High load resistance training Exp2: High repetition resistance training | Strength training | Maximum strength:  1-RM test for leg press |
| Bolton et al. (2012) [22] | 11 | 39 (w) | 58 (±5.15) | Overweighted / NA | Resistance training, impact loading and balance exercises | Strength training | Maximum strength: Maximum voluntary isometric knee extension on a iskinetic dynamometer  Static Balance:  Mediolateral CoP motion amplitudes during bilateral stance with eyes open |
| Bonfante et al. (2017) [23] | 12 | 22 (m) | 49.1 (±5.8) | Obese / Inactive | Concurrent resistance and endurance (walking/running) training | Strength-endurance training | Maximum strength:  1-RM test for leg press |
| Brunelli et al. (2015) [24] | 91 | 30 (m) | 48.73 (±1.5) | Obese / Inactive | Combined training of resistance exercise (leg press, leg extension, leg curl, bench press, lateral pulldown, and arm cur) and aerobic training (walking or running) | Strength-endurance training | Maximum strength:  1RM-test for leg press |
| Chasland et al. (2021) [25] | 99 | 40 (m) | 58.9 (±5.26) | Obese / NA | Machine-based cable resistance exercises alternated with aerobic cycling | Strength-endurance training | Maximum strength:  One-repetition maximum test (1RM), leg press |
| Chilibeck et al. (2013) [26] | 95 | 174 (w) | 55.89 (±6.72) | Overweighted / NA | Weight training (hack squat, hip abduction, adduction, flexion, and extension on a multihip machine, hamstrings curl, quadriceps extension, back extension, abdomen flexion, bench press, lat-pull down,  shoulder press, biceps curl, and triceps extension) and 20 to 30 minutes of brisk walking | Strength-endurance training | Maximum strenght:  1-RM test for hack squat  Dynamic Balance:  Gait speed over 80 meter distance  Proactive Balance:  Backwards tandem walking test |
| Colado & Triplett (2008) [27] | 16 | 45 (w) | 53.73 (±4.36) | NA / NA | Exp1: Resistance training using elastic bands Exp2: Resistance training using weight machines | Strength training | Strenght-endurance: 60-second squat test |
| Colado et al. (2009) [28] | 17 | 46 (w) | 54 (±2.4) | Overweighted / NA | Exp1: Upper and lower body aquatic exercises using resistance devices  Exp2: Resistance training using elastic bands | Exp1: Waterbased training  Exp2: Strength training | Strenght-endurance:  60-second squat test |
| Conceição et al. (2013) [29] | 18 | 20 (w) | 53.2 (±4.9) | Overweighted / Inactive | Resistance training: Leg press, leg extension, leg curl, bench press, lat pulldown, lateral raise, triceps pushdown, arm curl, and basic abdominal crunch | Strength training | Maximum strength:  1-RM test for leg press |
| Correa et al. (2015) [30] | 19 | 36 (w) | 58.9 (±5.8) | Overweighted / NA | Exp1: High volume resistance training Exp2: Low volume resistance training | Strength training | Maximum strength:  1-RM test for knee extension |
| Cotofana et al. (2010) [31] | 21 | 20 (w) | 50.08 (±2.83) | Overweighted / Inactive | Resistance training (superslow and hypertrophy mode) | Strength training | Maximum strength:  Maximal force by maximal voluntary isometric contraction on a leg press |
| Deibert et al. (2011) [32] | 26 | 22 (m) | 55.62 (±5.1) | Overweighted / NA | Resistance training programme performed on fitness equipment  (pull down, leg press, bench press, back press, etc.) | Strength training | Reactive Balance:  One-leg stabilisation test |
| De Jong et al. (2006) [33] | 22 | 181 (w+m) | 59.14 (±2.57) | Overweighted / NA | GALM program: leisure-time physical activity program with the 15 most favourite recreational sports activities | General physical activity | Proactive Balance: Functional Reach test  Power:  10-times-sit-to-stand test |
| Donges et al. (2013) [34] | 29 | 34 (m) | 49.08 (±0.344) | Exp1: Overweighted / Inactive  Exp2: Obese / Inactive | Exp1: Whole-body training program (chest and shoulder press, seated rows, lat pulldown, leg press, leg curls, lunges, machine squats, and deadlifts) Exp2: Concurrent resistance and endurance training | Exp1: Strength training  Exp2: Strength-endurance training | Maximum strength:  5-RM test for 45° leg press |
| Duft et al. (2017) [35] | 30 | 22 (m) | 48.2 (±6.1) | Obese / Inactive | Resistance training combined with walking/running | Strength-endurance training | Maximum strength:  1-RM test for leg press |
| Elliot et al. (2002) [36] | 27 | 15 (w) | 55.67 (±3.57) | Overweighted / NA | Low intensity, progressive resistance training: leg press, bench press, knee extension, knee flexion, and lat pull-down | Strength training | Maximum strength:  10-RM test for leg leg press |
| Figueroa et al. (2011) [37] | 28 | 24 (w) | 54 (±1.58) | Normal-weighted / Inactive | Combined circuit resistance training on weight machines (chest press, leg press, shoulder press, abdominal crunch, leg curl, leg extension, low back extension, bicep curls, and triceps extension) and endurance training | Strength-endurance training | Maximum strength:  1-RM test for leg extension |
| Figueroa et al. (2014) [38] | 33 | 25 (w) | 56 (±1) | Obese / Inactive | Whole-body vibration exercise combined with leg exercises | Whole-body vibration | Maximum strenght:  8-RM test for leg press |
| Flandez et al. (2016) [39] | 34 | 62 (w) | 46.47 (±3.71) | Obese / NA | Exp1: Strength training program using elastic tubing Exp2: Strength training using free weights | Strength training | Maximum strength:  Half-squat test with a load cell  Static Balance:  Unipedal dominant-leg balance test  Proactive Balance:  3-minutes-timed-up-and-go test |
| Fu et al. (2009) [40] | 31 | 50 (w) | 51.76 (±5.46) | NA / NA | Balance-strategy  training | Balance / Functional training | Maximum strength: Quadriceps muscle strength measured by using a spring gauge  Static Balance:  Modified Clinical Test for the Sensory Integration of Balance (bilateral stance, firm surface, eyes open)  Proactive Balance:  Timed-up-and-go test |
| Gillett et al. (1995) [41] | 37 | 82 (w) | NA | Obese / Inactive | Supervised low impact dance exercise | Aerobic | Maximum strength:  1-RM test for leg press |
| Granacher et al. (2011) [42] | 39 | 32 (w+m) | 55.77 (±3.56) | Overweighted / NA | Static balance, dynamic balance and lower limbs' strength enhancing exercises conducted in the office | Balance / Functional training | Maximum strength:  Maximal isometric, isokinetic rate of torque development of the plantar flexors  Power:  Vertical ground reaction force during countermovement jumps  Static Balance:  Total CoP displacements under static conditions  Dynamic Balance:  Habitual gait speed on 12 meter walk |
| Holviala et al. (2014,women) [43] | 41a | 68 (w) | 58 (±6.31) | Overweighted / NA | Full-body programme for the lower and upper extremities and torso, mainly on weight machines | Strength training | Maximum strength:  1-RM test for leg press  Proactive Balance:  Moving a cursor at the straightest possible line on a fixed force platform  Dynamic Balance:  10 meter walking time at maximal velocity |
| Holviala et al. (2014, men) [43] | 41b | 58 (m) | 58.41 (±6) | Overweighted / NA | Full-body programme for the lower and upper extremities and torso, mainly on weight machines | Strength training | Maximum strength:  1-RM test for leg press  Proactive Balance:  Moving a cursor at the straightest possible line on a fixed force platform  Dynamic Balance:  10 meter walking time at maximal velocity |
| Janzen et al. (2006) [44] | 42 | 40 (w) | 57 (±7.11) | NA / NA | Exp1: Bilateral strength training (leg press, knee extension, and hamstring curl, lat pull-down, biceps curl, shoulder press, and chest press) Exp2: Unilateral strength training (see Exp1) | Strength training | Maximum strength:  1-RM test for leg press (bilateral) |
| Jørgensen et al. (2011) [45] | 43 | 195 (w) | 44.51 (±9) | Overweighted / NA | Functional coordination exercise (four point kneeling, prone plank, bridge, vertical plank, bodyblade and horizontal side support) | Strength training | Static Balance:  30 sec balance test on a force platform (95% confidence ellipse) |
| Kak et al. (2013) [46] | 44 | 40 (w) | 54.9 (±3.98) | NA / Inactive | Aerobic physical activity and resistance training | Strength-endurance training | Maximum strength:  NA |
| Karatrantou et al. (2017) [47] | 46 | 54 (w) | 46.7 (±4.5) | NA / Inactive | Exp1: Serial strength and endurance training Exp2: Integrated strength and aerobic training | Strength training + Aerobic | Maximum strength: Iskonetic peak torque of knee extensors  Power:  Squat jump test  Static Balance:  1-minute single limb stance test (eyes open, right leg)  Proactive Balance:  Timed-up-and-go test |
| Karavirta et al. (2011) [48] | 53 | 71 (m) | 55.77 (±6.91) | Overweighted / NA | Exp1: High-intensity strength training (seated calf raise, hip abduction or adduction, bench press, biceps curl, triceps pushdown, lateral pull-down, abdominal crunch, seated back extension) Exp2: Concurrent strength (see Exp1) and endurance (bicycle ergometer) training | Strength training | Maximum strength:  1-RM test for leg press  Power:  Maximum concentric power on dynamic leg press |
| Kim et al. (2012) [49] | 48 | 34 (w) | 44.38 (±1) | Overweighted / NA | Yoga | 3D | Maximum strength:  1-RM test for leg press |
| Klentrou et al. (2007) [50] | 49 | 16 (w) | 53.01 (±4.81) | NA / NA | Supervised, multimodal training program wearing weighted vests | Balance / Functional training | Maximum strength: Isokinetic peak torque in knee extension (180°/sec) |
| Kloubec et al. (2010) [51] | 50 | 44 (w+m) | 44.45 (±8.58) | Overweighted / NA | Pilates exercises | Strength training | Static Balance:  1-minute balance board test |
| Kostić et al. (2015) [52] | 51 | 63 (w) | NA | NA / NA | Greek folk dancing | 3D | Static Balance:  Single leg stance test  Proactive Balance: Functional Reach test |
| Libardi et al. (2012a) [53] | 52 | 35 (m) | 48.98 (±5.35) | Overweighted / NA | Exp1: Lower body exercises (leg press, leg extension, and leg curl) and upper body exercises (bench press, lateral pulldown, lateral raise, triceps pushdown, arm curl, and basic abdominal crunch) Exp2: Concurrent resistance (see Exp1) and endurance (walking/running) training | Exp1: Strength training  Exp2: Strength-endurance training | Maximum strength:  1-RM test for leg press |
| Libardi et al. (2012bmen) [54] | 94a | 26 (m) | 48.08 (±5.03) | Overweighted / Inactive | High volume, low intensity resistance training (leg press, leg extension and leg curl) and six upper body exercises  (bench press, lateral pulldown, lateral raise, triceps pushdown, arm curl, basic abdominal crunch) | Strength training | Maximum strenght:  1-RM test for leg press |
| Libardi et al. (2012bwomen) [54] | 94b | 24 (w) | 52.42 (±5.23) | Overweighted / Inactive | High volume, low intensity resistance training (leg press, leg extension and leg curl) and six upper body exercises  (bench press, lateral pulldown, lateral raise, triceps pushdown, arm curl, basic abdominal crunch) | Strength training | Maximum strenght:  1-RM test for leg press |
| Liphardt et al. (2015) [55] | 90 | 31 (w) | 58.83 (±4.06) | NA / NA | Low-level whole-body vibration | Whole-body vibration | Maximum strength:  Maximal isometric knee extension torque on a dynamometer  Static Balance:  Static postural stability on a force plate (eyes open, no foam) |
| Marcus et al. (2009) [56] | 54 | 16 (w) | 56.1 (±6.4) | NA / NA | Eccentric resistance exercise to the knee extensors | Strength training | Maximum strength:  Maximal isometric force measures of the knee extensors at 90° flexion. |
| Marston et al. (2019) [57] | 55 | 44 (w+m) | 57.48 (±6.61) | Overweighted / NA | Exp1: Highload, long rest resistance training (bench press, leg press, lat pull-down and leg curl) Exp2: Moderate-load, short rest resistance training (see Exp1) | Strength training | Maximum strength:  1-RM test for leg press |
| Moreno-Muñoz et al. (2021) [58] | 100 | 117 (w) | 45.65 (±8.86) | Normal-weighted / NA | Abdominal Hypopressive Training | Balance/ Functional training | Static Balance:  Assessed with a stabilometric platform (mediolateral mean displacements of the center of pressure) |
| Nelson et al. (1994) [59] | 58 | 39 (w) | 59.25 (±5.13) | Normal-weighted / Inactive | High-intensity strength training (hip extension, knee  extension, lateral pull-down, back extension, and abdominal flexion using resistance machines) | Strength training | Maximum strength:  1-RM test for leg press  Proactive Balance:  Timed backward tandem walk test over a 20-foot course |
| Neves et al. (2017) [60] | 59 | 50 (w) | 58.2 (±4.32) | Overweighted / Inactive | Functional exercise program: resistance training exercises, agility drills, coordination, balance exercises | Balance / Functional training | Proactive Balance:  Agility and dynamic balance test (from battery of motor tests from the AAHPERD) |
| Nunes et al. (2017) [61] | 61 | 23 (w) | 59.56 (±NA) | Overweighted / NA | High volume resistance training (i.e. spine flexion, extension, and  rotation; shoulder and elbow flexion and extension; hip and knee flexion and extension) | Strength training | Maximum strength:  Sum of lower limb 1-RM tests  Dynamic Balance:  Walking speed at one mile walk test |
| Palumbo et al. (2012)[62] | 97 | 11 (w) | 54.4 (NA) | NA / NA | Tai Chi group exercise | 3D | Maximum strength: Isometric knee extensor strength test, dynamometer  Proactive Balance:  Functional Reach Test |
| Paoli et al. (2010) [63] | 63 | 40 (NA) | 56 (±2.7) | NA / NA | Exp1: Circuit low intensity exercises with alternating endurance (treadmill) and resistance (back: underhand cable pulldowns, Chest: chest press, shoulders: lateral shulder raise; lower limbs: horizontal press; abdomen: abdominal crunches) exercise Exp2: Circuit high intensity exercises (see Exp1) | Strength-endurance training | Maximum strength:  6-RM test for horizontal leg press |
| Park et al. (2015) [64] | 88 | 20 (w) | 57.2 (±2.17) | Overweighted / NA | Combined Exercise Program using resistance equipment and a treadmill. | Strength-endurance training | Static Balance:  Closed-eyes one-legged standing time |
| Perchthaler et al. (2015) [65] | 70 | 21 (w+m) | 54.75 (±8.08) | Overweighted / NA | Whole-Body Vibration training | Whole-body vibration | Maximum strength:  Peak torque by maximal isokinetic knee extension  Power:  Vertical countermovement jump heigth |
| Reis et al. (2012) [66] | 65 | 51 (w) | 53.36 (±4.57) | NA / NA | Muscle strengthening exercises (quadriceps, hamstring, calf, tibialis anterior, gluteus maximus, and abdominal muscles) using ankle weights | Strength training | Maximum strenght:  1-RM test for quadriceps muscle |
| Sallinen et al. (2007) [67] | 92 | 39 (m) | 58.05 (±6.36) | Normal-weighted / NA | Whole body progressive strength training (bilateral leg press, bilateral/unilateral  knee extension, bilateral/ unilateral knee flexion, standing calf machine, machine bench press and pec decmachine, lateral pull down, elbow flexion and extension, abdominal crunch, trunk rotation and extension, leg adduction and abduction) | Strength training | Maximum strength:  1RM-test for horizontal leg press  Dynamic Balance:  Walking speed over 10 meter distance |
| Shaw et al. (2016) [68] | 68 | 37 (w) | 59.13 (±4.31) | Overweighted / NA | Hypertrophic resistance training (dumbbell pelvic lifts, machine leg presses, barbell squats, machine hip adduction, machine hip abduction, and machine standing calf raises) | Strength training | Maximum strenght:  6-RM test for leg press |
| Shirazi et al. (2006) [69] | 69 | 116 (w) | 53.17 (±8.35) | Overweighted / NA | Progressive, individually tailored exercise program, including a walking program as a weight-bearing exercise | Strength training | Maximum strength:  1-RM for knee extensors  Static Balance:  Functional Reach test |
| Singh et al. (2009) [70] | 72 | 54 (w) | 41.21 (±1.25) | Overweighted / NA | strength training (15 week supervised + 24 week unsupervised) using exercise equipment (squats, leg press, leg extension, seated leg curl, lat pulldowns) and free weight exercises (bench press, overhead press, biceps curls, and triceps extension) | Strength training | Maximum strength:  1-RM test for leg press |
| Sipilä et al. (2001) [71] | 76 | 27 (w) | NA | NA / NA | Physical training programme (skipping, bounding over soft hurdles, drop jumping and hopping) that included a supervised circuit training session twice a week and a series of exercises at home on 4 days per week interrupted by three high-impact aerobic dance periods | Balance / Functional training | Maximum strength:  Maximal isometric knee extension force  Power:  Vertical countermovement jump heigth |
| Smith et al. (2017) [72] | 77 | 16 (w+m) | 57.73 (±5.33) | NA / NA | 8-week homebased  resistanc-band-exercise programme (squat, stiff leg deadlift, hip abduction, reverse flies, chest press, side lateral raise, and biceps curl, bent-over row, chest flies, overhead press, and elbow kick-back) | Strength training | Strenght-endurance:  60-second squat test  Proactive Balance: Functional Reach test |
| Taaffe et al. (2005) [73] | 86 | 27 (w) | NA | NA / NA | High-impact training for the lower limbs (bounding, drop jumping, hopping and skipping), four resistance training exercise for upper body. Five training periods of 8–10 weeks were interspersed with three high-impact aerobic dance periods of 2 weeks. | Balance / Functional training | Maximum Strength: Isometric knee extension strength at a knee angle of 60° from full extension  Power: Vertical Countermovement jumping heigth  Dynamic Balance:  Running speed over 20 meter |
| Tsourlou et al. (2003) [74] | 82 | 31 (w) | 41.61 (±5.39) | Normal-weighted / Inactive | Exp1: Moderate-impact aerobic choreography and calisthenics (squats, static knee extensions–hip flexions (stationary lunges), and lunges)  Exp2: Moderate-impact aerobic choreography and exercises on weight machines (leg extensions,  leg curls, and hip extensions) | Strength training + Aerobic | Maximum strength:  Maximal isometric knee extension  Power:  Squat jump heigth |
| Uusi-Rasi et al. (2003) [75] | 93 | 76 (w) | 53.3 (±2.2) | Overweighted / NA | Multidirectional jumping  Exercises and calisthenics (stretching and nonimpact  exercises) | Balance / Functional training | Maximum strength:  Maximal isometric strength on a leg press dynamometer  Power:  Vertical countermovement jumping heigth  Static Balance:  Postural sway during static stance on a postural sway platform  Proactive Balance:  Figure-eight-running test |
| Wen et al. (2016) [76] | 96 | 46 (w) | 58.2 (±3.4) | Normal-weighted / NA | Group-based step aerobic exercise (conventional basic step, the V-step, the L-step, alternating step knee-lift sequences, alternating leg curl, side-leg, and leg-back patterns as well as arm movements: bicep curls, lateral raises at shoulder level and above the head) | Aerobic | Power:  Chair stand test  Proactive Balance:  8-feet-up-and-go test |
| Wittke et al. (2017) [77] | 84 | 80 (m) | 42.7 (±5.5) | Overweighted / NA | High intensity, low volume resistance exercise training ((latissimus back and front pulleys, front chin ups, seated rowing, back extension, inverse fly, hyperextension, sitting bench press, shoulder-press, military press, butterfly with extended arms, crunches, leg press, leg extension, leg curls, leg adduction, and abduction) | Strength training | Maximum strength:  Dynamic leg and hip extensor strength using an Isokinet |
| Wong et al. (2018) [78] | 83 | 41 (w) | 59 (±1) | Normal-weighted / Inactive | One to five stair climbs of 12 fligths on 4 days a week | Balance / Functional training | Maximum strength:  8-RM test for leg extension |
| Zhao et al. (2007) [79] | 85 | 78 (w) | 54.76 (±3.15) | NA / NA | Exp1: Tai chi chuan  Exp2: Tai chi chuan + calcium supplementation | 3-Dimensional intervention | Static Balance:  One leg standing time |

1RM: one-repetition-maximum; m: men; N: number of participants; NA: not applicable; SD: standard deviation; SE: standard error; Sess. Dur.: Session Duration; w: women; y: years

Supplement 7: Full results

Table of Contents

[1 Main results 64](#_Toc106981995)

[1.1 Overall-strength 64](#_Toc106981996)

[1.2 Overall-balance 66](#_Toc106981997)

[2 Subdomains of lower limb muscle strength 68](#_Toc106981998)

[2.1 Strength Max 68](#_Toc106981999)

[2.2 Muscle Power 71](#_Toc106982000)

[2.3 Strength Endurance 74](#_Toc106982001)

[3 Subdomains of postural balance 76](#_Toc106982002)

[3.1 Static balance 76](#_Toc106982003)

[3.2 Dynamic balance 79](#_Toc106982004)

[3.3 Proactive balance 81](#_Toc106982005)

[3.4 Reactive Balance 84](#_Toc106982006)

[4 Subgroup and sensitivity analyses 85](#_Toc106982007)

[4.1 Subgroup analyses 85](#_Toc106982008)

[4.1.1 Subgroup analyses for overall-strength 85](#_Toc106982009)

[4.1.1.1 Lower-aged subgroup 85](#_Toc106982010)

[4.1.1.2 Higher-aged subgroup 87](#_Toc106982011)

[4.1.1.3 Male populations 89](#_Toc106982012)

[4.1.1.4 Female populations 91](#_Toc106982013)

[4.1.1.5 Higher-aged women 93](#_Toc106982014)

[4.1.1.6 Inactive populations 95](#_Toc106982015)

[4.1.1.7 Inactive higher-aged women 97](#_Toc106982016)

[4.1.2 Subgroup analyses for postural balance 99](#_Toc106982017)

[4.1.2.1 Lower-aged subgroup 99](#_Toc106982018)

[4.1.2.2 Higher-aged subgroup 101](#_Toc106982019)

[4.1.2.3 Male populations 103](#_Toc106982020)

[4.1.2.4 Female populations 104](#_Toc106982021)

[4.1.2.5 Higher-aged women 106](#_Toc106982022)

[4.1.2.6 Inactive populations 108](#_Toc106982023)

[4.1.2.7 Inactive higher-aged women 110](#_Toc106982024)

[5 Sensitivity analyses 112](#_Toc106982025)

[5.1 PEDRO Score of 6 or higher 112](#_Toc106982026)

[5.1.1 Overall-strength 112](#_Toc106982027)

[5.1.2 Overall-balance 114](#_Toc106982028)

[5.1.3 Subdomains of muscle strength 116](#_Toc106982029)

[5.1.3.1 Maximum strength 116](#_Toc106982030)

[5.1.3.2 Muscle Power 118](#_Toc106982031)

[5.1.3.3 Strength-endurance 120](#_Toc106982032)

[5.1.4 Subdomains of postural balance 120](#_Toc106982033)

[5.1.4.1 Static balance 120](#_Toc106982034)

[5.1.4.2 Dynamic balance 122](#_Toc106982035)

[5.1.4.3 Proactive balance 123](#_Toc106982036)

# Main results

## Overall-strength

Number of studies: k = 52
Number of pairwise comparisons: m = 62
Number of treatments: n = 10
Number of active components: c = 8
Number of designs: d = 12

Results for combinations (additive model, random effects model):
 SMD 95%-CI z p-value
Step aerobic 0.3884 [-0.2248; 1.0016] 1.24 0.2145
control 0.0000 [ 0.0000; 0.0000] -- --
Balance/Functional 0.5742 [-0.0308; 1.1795] 1.86 0.0629
3-Dimensional 0.7907 [-0.6957; 2.2770] 1.04 0.2971
GenPhyAct -0.0395 [-1.3820; 1.3030] -0.06 0.9540
Strength 1.0151 [ 0.7319; 1.2984] 7.02 < 0.0001
Strength-Aerobic 1.4085 [ 0.8004; 2.0166] 4.54 < 0.0001
Strength-Endurance 0.9216 [ 0.4872; 1.3561] 4.16 < 0.0001
WBV 0.4669 [-0.4221; 1.3559] 1.03 0.3033
Waterbased 1.0768 [ 0.0586; 2.0949] 2.07 0.0382


Quantifying heterogeneity / inconsistency:
tau^2 = 0.4467; tau = 0.6684; I^2 = 79.3 [73.2%; 84.0%]

Heterogeneity statistics:
 Q df p-value
Additive model 237.08 49 0
Standard model 202.42 48 < 0.0001
Difference 34.66 1 < 0.0001


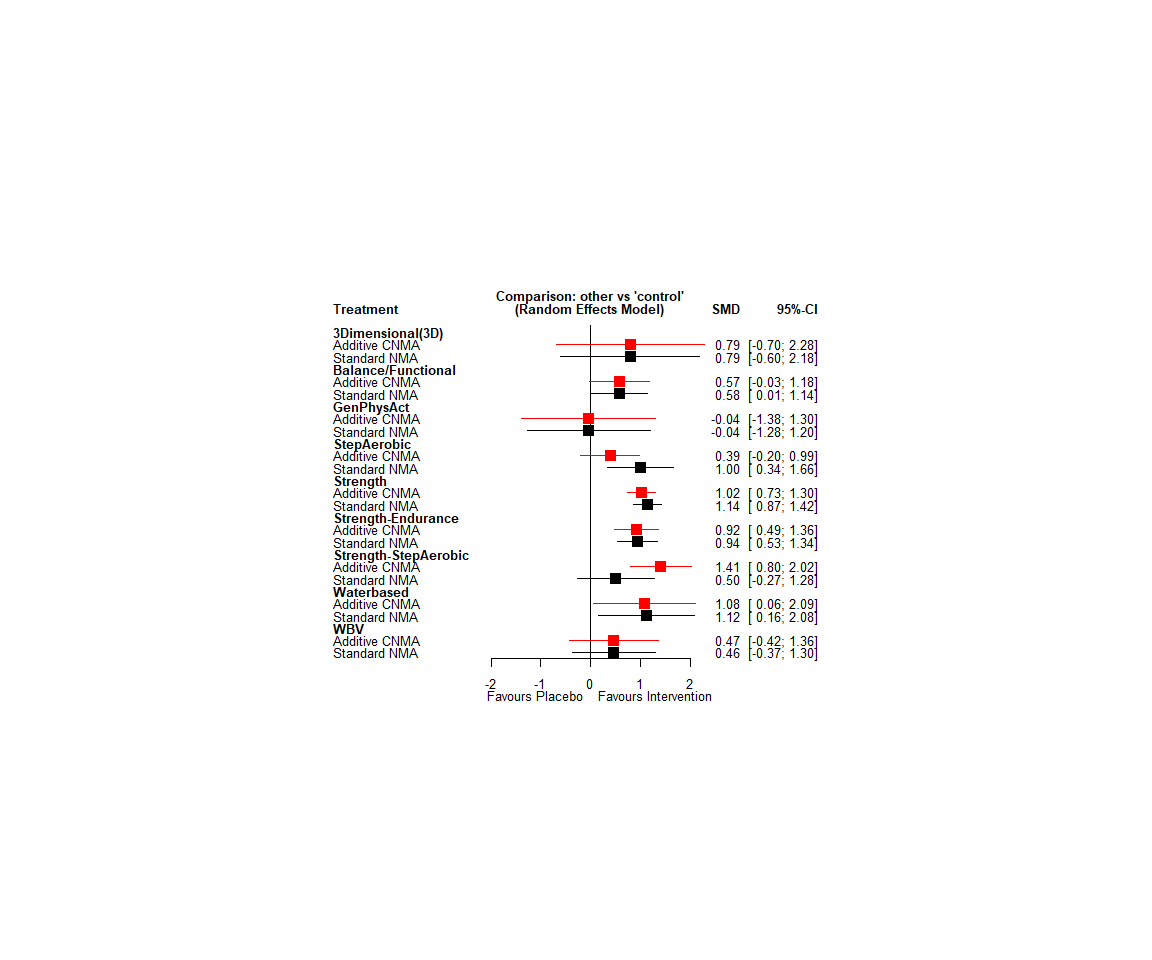


Supplementary Figure S1 Forest plot: Effects of physical activity interventions on overall-strength. GenPhysAct: General Physical Activity. WBV: Whole Body Vibration.

##

## Overall-balance

Number of studies: k = 30
Number of pairwise comparisons: m = 32
Number of treatments: n = 10
Number of active components: c = 8
Number of designs: d = 10

Results for combinations (additive model, random effects model):
 SMD 95%-CI z p-value
Step aerobic -0.1820 [-1.0043; 0.6403] -0.43 0.6645
control 0.0000 [ 0.0000; 0.0000] -- --
Balance/Functional 0.4822 [-0.1912; 1.1556] 1.40 0.1604
GenPhyAct -0.2129 [-1.7967; 1.3708] -0.26 0.7922
Strength 1.1605 [ 0.7017; 1.6912] 4.96 < 0.0001
Strength-Aerobic 0.9785 [ 0.1244; 1.8326] 2.25 0.0247
Strength-Endurance 0.1120 [-1.0733; 1.2972] 0.19 0.8531
WBV 0.1694 [-1.5405; 1.8793] 0.19 0.8461
Waterbased -0.2823 [-2.0430; 1.4784] -0.31 0.7533


Quantifying heterogeneity / inconsistency:
tau^2 = 0.6304; tau = 0.7939; I^2 = 88.1% [83.5%; 91.4%]

Heterogeneity statistics:
 Q df p-value
Additive model 192.70 23 0
Standard model 190.44 22 < 0.0001
Difference 2.27 1 0.1322


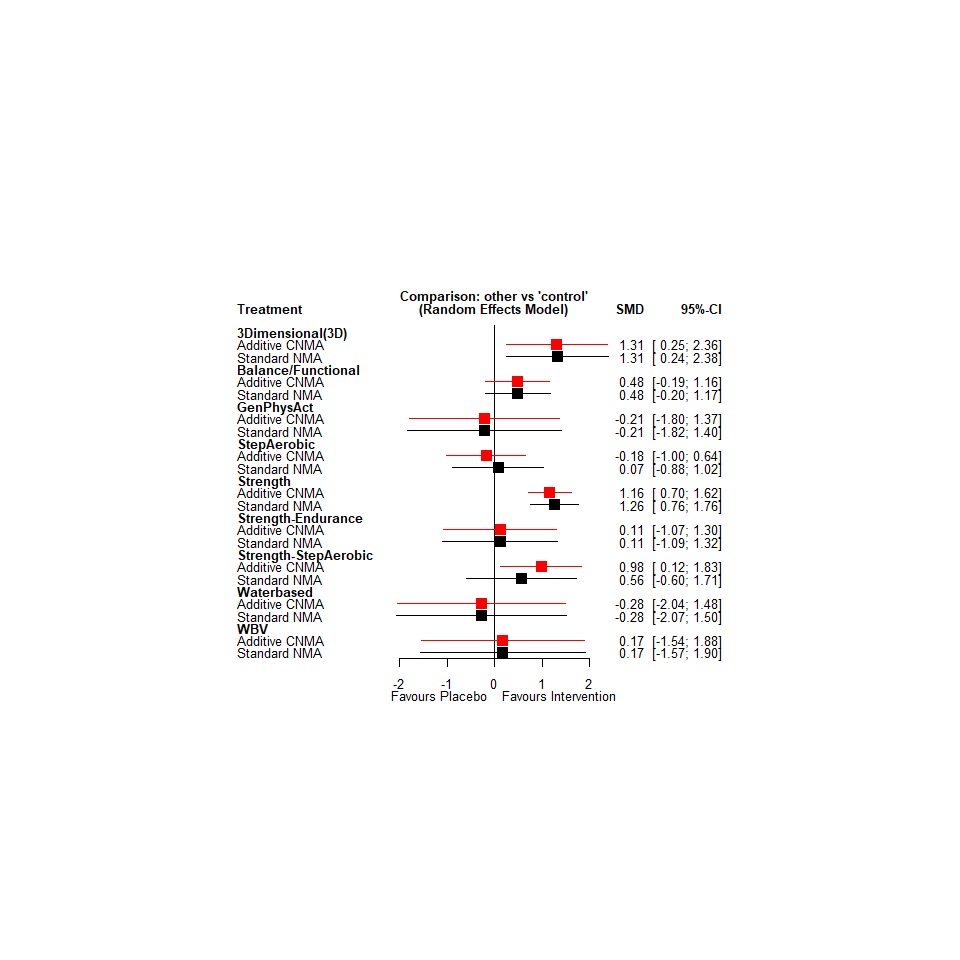


Supplementary Figure S2 Forest plot: Effects of physical activity interventions on overall-balance. GenPhysAct: General Physical Activity. WBV: Whole Body Vibration.

#

# Subdomains of lower limb muscle strength

## Maximum Strength


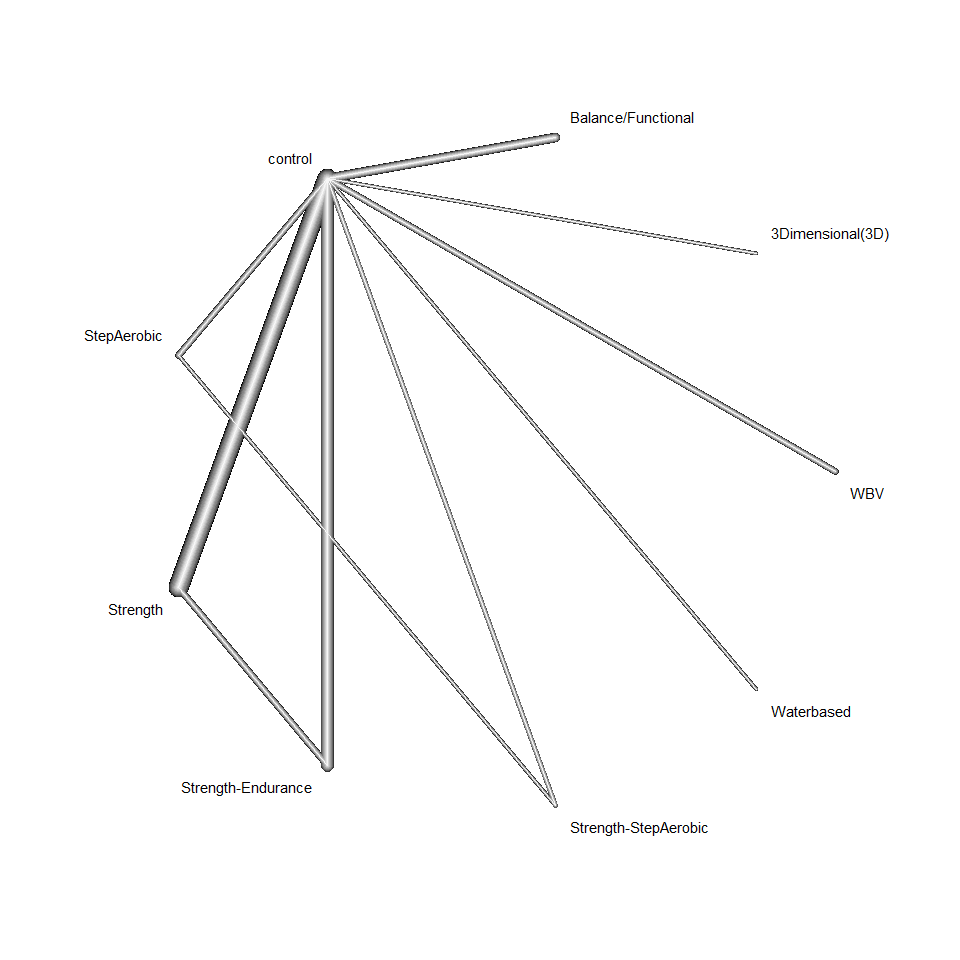


Supplementary Figure S3 Network of studies measuring maximum strength

Number of studies: k = 46
Number of pairwise comparisons: m = 54
Number of treatments: n = 9
Number of active components: c = 7
Number of designs: d = 10

Results for combinations (additive model, random effects model):
 SMD 95%-CI z p-value
Step aerobic 0.4659 [-0.2506; 1.1824] 1.27 0.2025
control 0.0000 [ 0.0000; 0.0000] -- --
Balance/Functional 0.5029 [-0.1081; 1.1140] 1.61 0.1067
3-Dimensional 0.7907 [-0.7107; 2.2920] 1.03 0.3020
Strength 1.2277 [ 0.9205; 1.5348] 7.83 < 0.0001
Strength-Aerobic 1.6936 [ 0.9610; 2.4262] 4.53 < 0.0001
Strength-Endurance 1.0536 [ 0.6135; 1.4937] 4.69 < 0.0001
WBV 0.2223 [-0.6715; 1.1161] 0.49 0.6259
Waterbased 0.7936 [-0.7821; 2.3694] 0.99 0.3236

Quantifying heterogeneity / inconsistency:
tau^2 = 0.4584; tau = 0.6770; I^2 = 80% [73.8%; 84.8%]

Heterogeneity statistics:
 Q df p-value
Additive model 215.53 43 0
Standard model 201.22 42 < 0.0001
Difference 14.31 1 0.0002


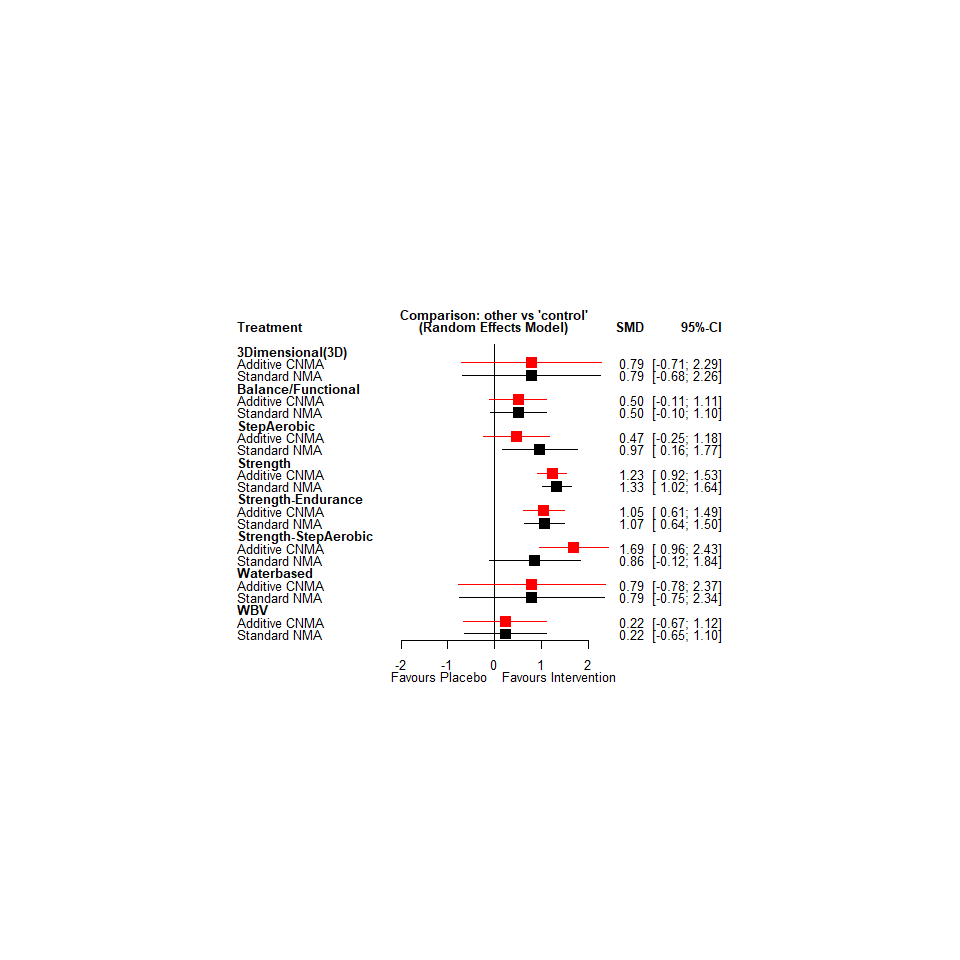


Supplementary Figure S4 Forest plot: Effects of physical activity interventions on maximum strength. WBV: Whole Body Vibration.

## Muscle Power


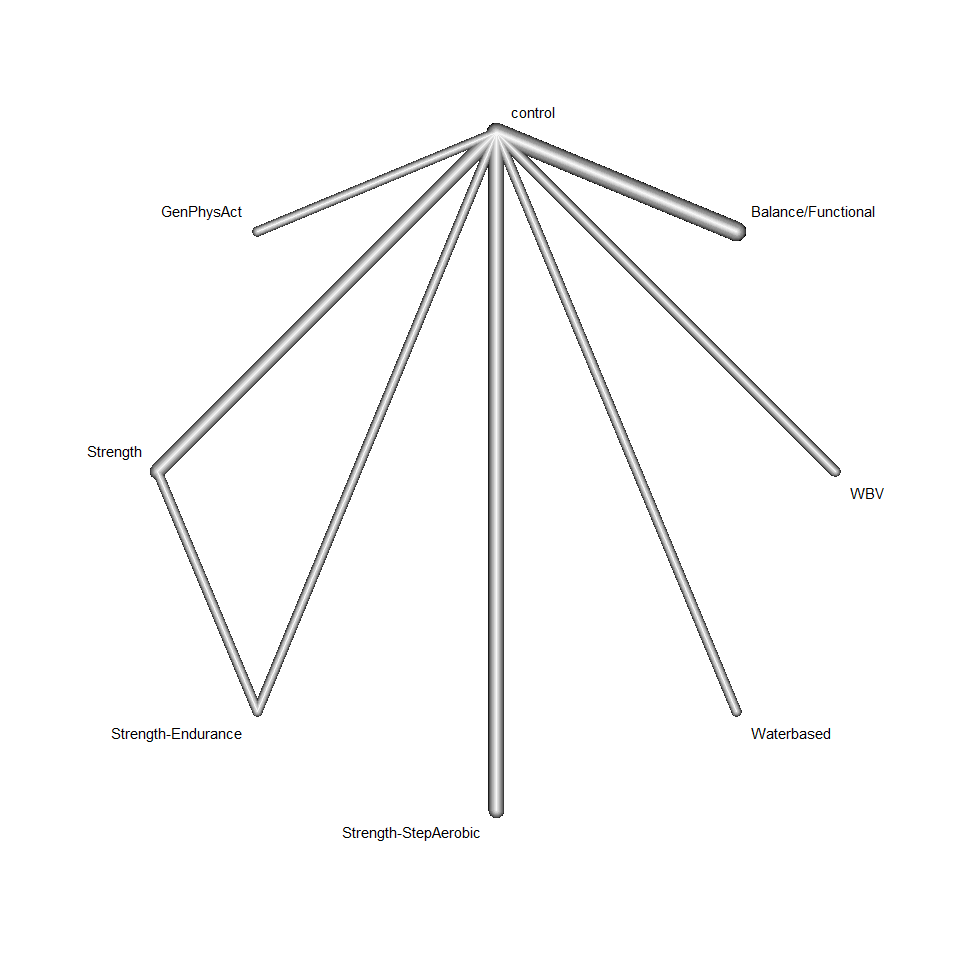


Supplementary Figure S5 Network of studies measuring muscle power. GenPhysAct: General Physical Activity. WBV: Whole Body Vibration.

Number of studies: k = 10
Number of pairwise comparisons: m = 12
Number of treatments: n = 8
Number of active components: c = 7
Number of designs: d = 7

Results for combinations (additive model, random effects model):
 SMD 95%-CI z p-value
control 0.0000 [ 0.0000; 0.0000] -- --
Balance/Functional 0.4616 [0.1187; 0.8046] 2.64 0.0083
GenPhyAct -0.0395 [-0.3333; 0.2543] -0.26 0.7920
Strength 0.3852 [-0.1434; 0.9137] 1.43 0.1532
Strength-Aerobic 0.3471 [-0.1268; 0.8210] 1.44 0.1511
Strength-Endurance 0.4622 [-0.1143; 1.0388] 1.57 0.1161
WBV 0.7755 [-0.1424; 1.6934] 1.66 0.0978
Waterbased 0.2324 [-0.5901; 1.0549] 0.55 0.5797

Quantifying heterogeneity / inconsistency:
tau^2 = 0; tau = 0; I^2 = 0% [0.0%; 79.2%]

Heterogeneity statistics:
 Q df p-value
Additive model 2.36 4 0.6707
Standard model 2.36 4 0.6707
Difference 0 0 - -


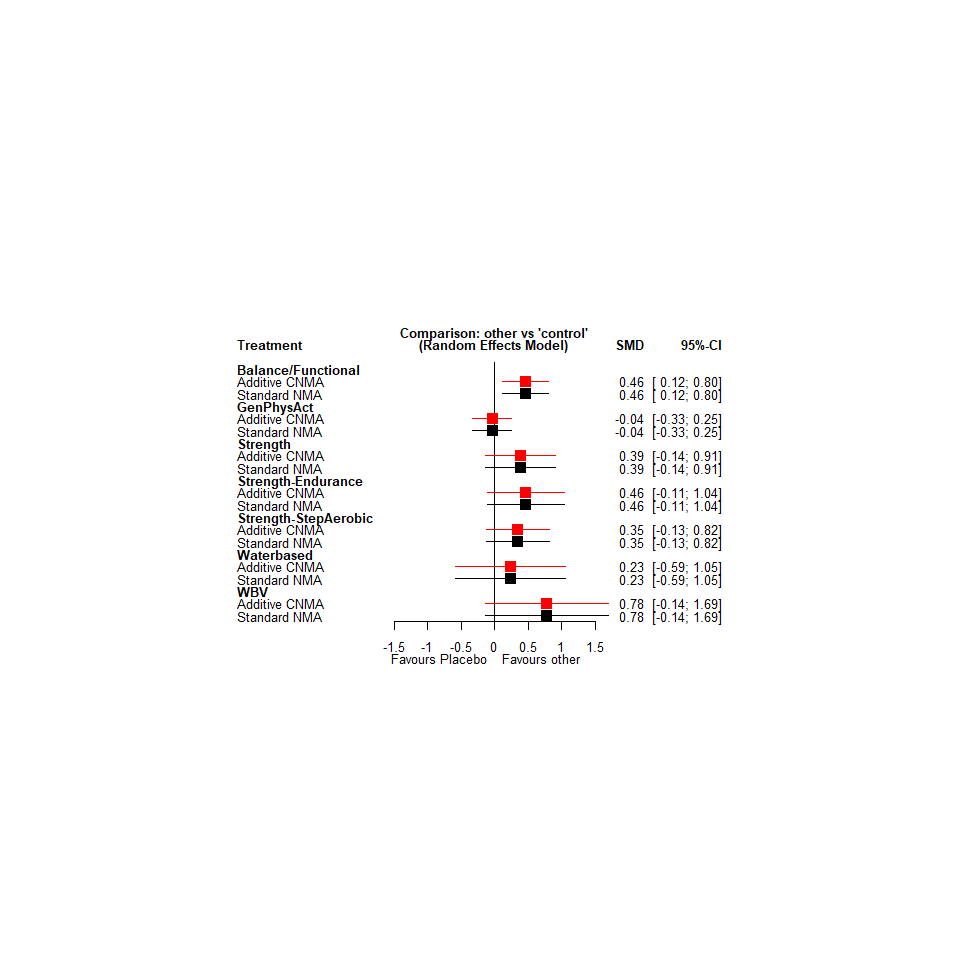


Supplementary Figure S6 Forest plot: Effects of physical activity interventions on muscle power. GenPhysAct: General Physical Activity. WBV: Whole Body Vibration.

## Strength Endurance


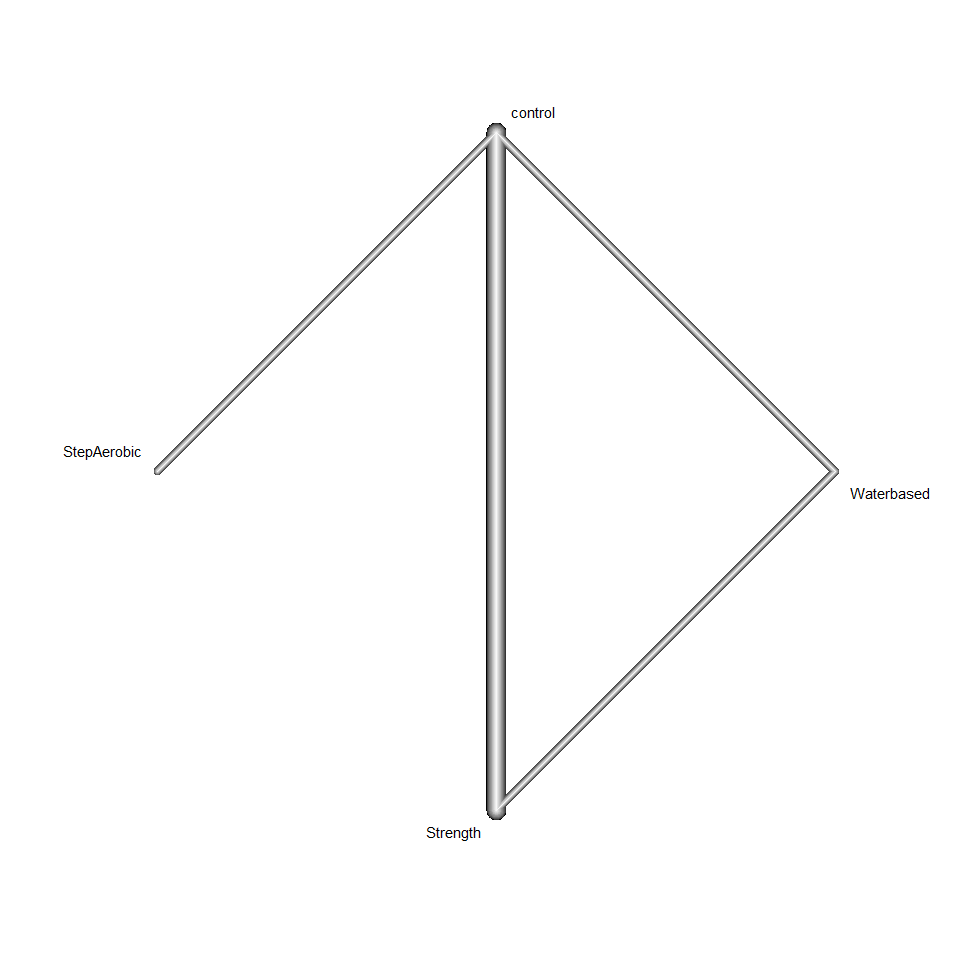


Supplementary Figure S7 Network of studies measuring strength-endurance

Results (random effects model):

Number of studies: k = 4
Number of pairwise comparisons: m = 6
Number of treatments: n = 3
Number of designs: d = 2

Random effects model

Treatment estimate (sm = 'SMD', comparison: other treatments vs 'control'):
 SMD 95%-CI z p-value
control 0.000 -.- -.- -.- . . .
Strength 1.1147 [0.5571; 1.6723] 3.92 <0.0001
Waterbased 1.7593 [0.6264; 2.8922] 3.04 0.0023
Step aerobic 1.3919 [0.2572; 2.5266] 2.40 0.0162

Quantifying heterogeneity / inconsistency:
tau^2 = 0.2251; tau = 0.4745; I^2 = 58.8% [0.0%; 84.6%]

Tests of heterogeneity (within designs) and inconsistency (between designs):
 Q d.f. p-value
Total 9.70 4 0.0458
Within designs 8.98 2 0.0295
Between designs 0.73 1 0.3939


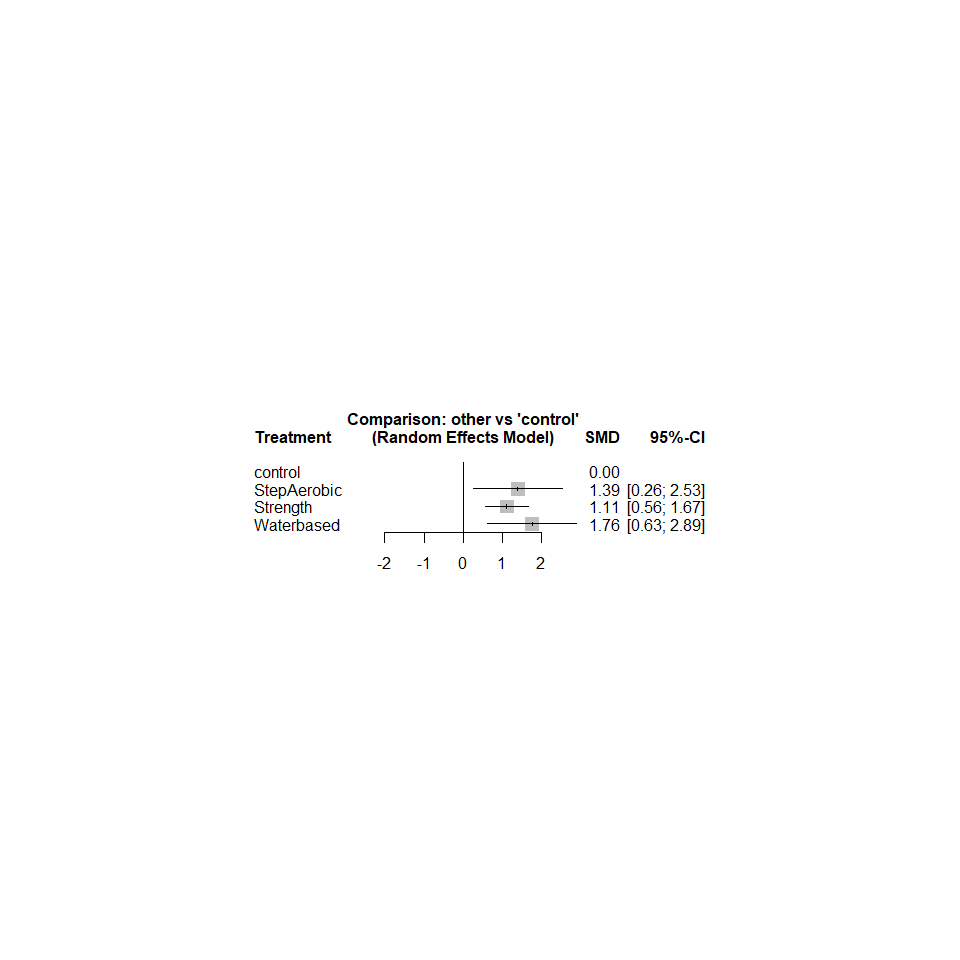


Supplementary Figure S8 Forest plot: Effects of physical activity interventions on strength-endurance

# Subdomains of postural balance

## Static balance


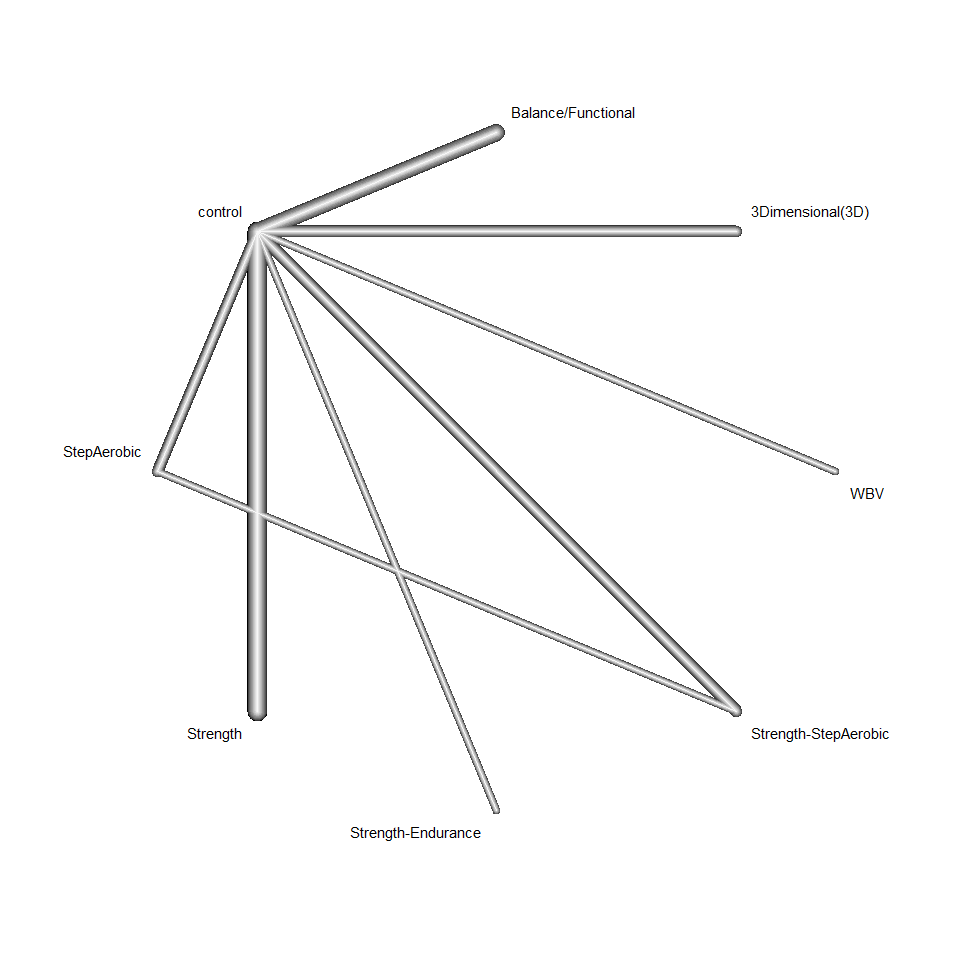


Supplementary Figure S9 Network of studies measuring static balance. WBV: Whole Body Vibration.

Number of studies: k = 16
Number of pairwise comparisons: m = 18
Number of treatments: n = 8
Number of active components: c = 6
Number of designs: d = 8

Results for combinations (additive model, random effects model):
 SMD 95%-CI z p-value
3Dimensional (3D) 0.2035 [-0.4370; 0.8441] 0.62 0.5335
Step aerobic -0.1345 [-0.6964; 0.4274] -0.47 0.6390
control 0.0000 [ 0.0000; 0.0000] -- --
Balance/Functional 0.2304 [-0.2283; 0.6890] 0.98 0.3249
Strength 0.4117 [ 0.0490; 0.7745] 2.22 0.0261
Strength-Aerobic 0.2773 [-0.2920; 0.8465] 0.95 0.3398
Strength-Endurance 0.0583 [-1.1020; 1.2185] 0.10 0.9216
WBV 0.1694 [-0.8698; 1.2086] 0.32 0.7494

Quantifying heterogeneity / inconsistency:
tau^2 = 0.1503; tau = 0.3877; I^2 = 69.4% [44.5%; 83.1%]

Heterogeneity statistics:
 Q df p-value
Additive model 35.97 11 0.0002
Standard model 35.19 10 0.0001
Difference 0.79 1 0.3755


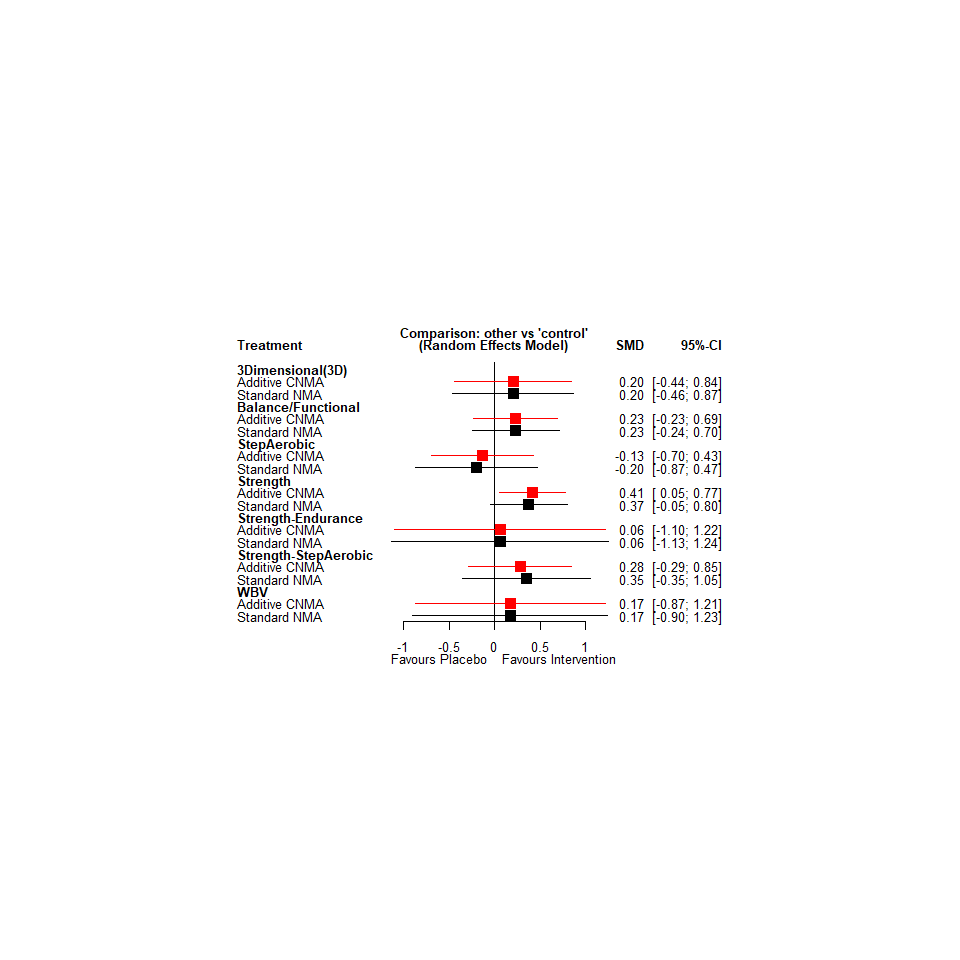


Supplementary Figure S10 Forest plot: Effects of physical activity interventions on static balance. WBV: Whole Body Vibration.

## Dynamic balance


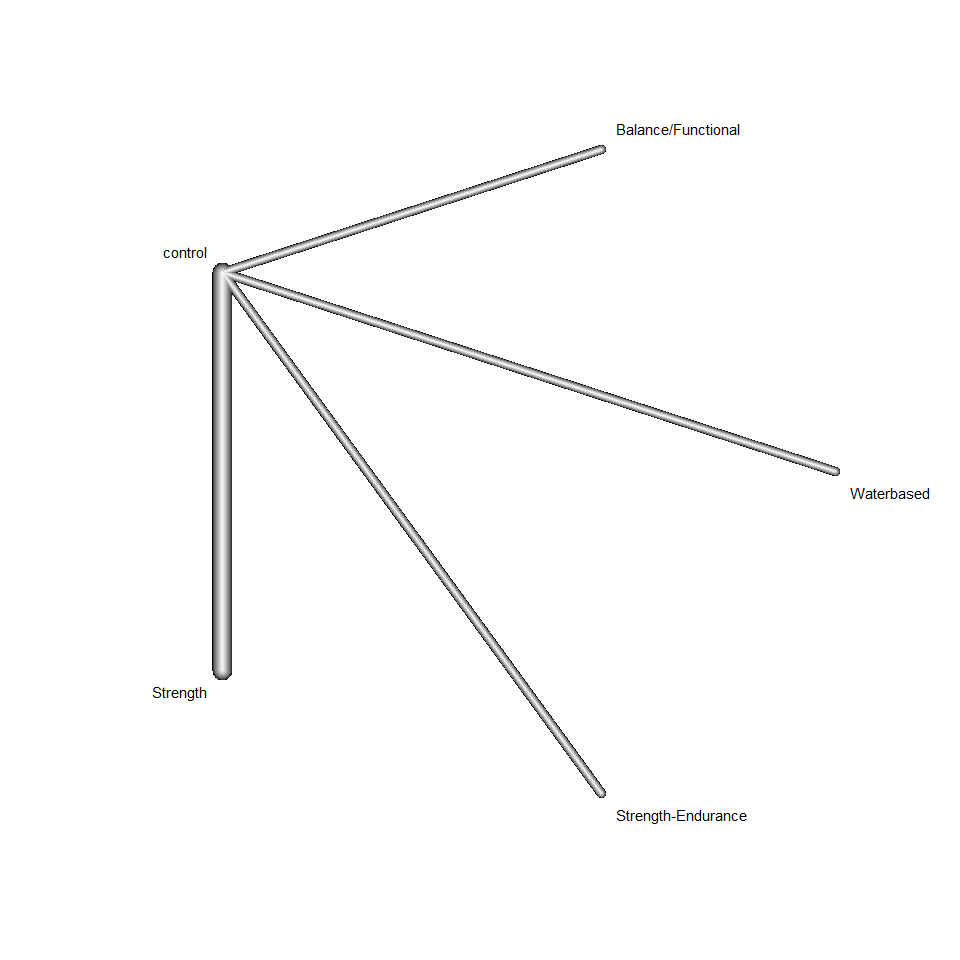


Supplementary Figure S11 Network of studies measuring dynamic balance

Number of studies: k = 5
Number of pairwise comparisons: m = 5
Number of treatments: n = 5
Number of active components: c = 4
Number of designs: d = 4

Results for combinations (additive model, random effects model):
 SMD 95%-CI z p-value
control 0.0000 [ 0.0000; 0.0000] -- --
Balance/Functional 0.2950 [-2.1768; 2.7668] 0.23 0.8150
Strength 2.3112 [ 1.0528; 3.5695] 3.60 0.0003
Strength-Endurance 0.1542 [-2.2355; 2.5438] 0.13 0.8994
Waterbased -0.2823 [-2.7924; 2.2277] -0.22 0.8255

Quantifying heterogeneity / inconsistency:
tau^2 = 1.4635; tau = 1.2097; I^2 = 89.5% [76.0%; 95.4%]

Heterogeneity statistics:
 Q df p-value
Additive model 28.65 3 < 0.0001
Standard model 28.65 3 < 0.0001
Difference 0.00 0 --


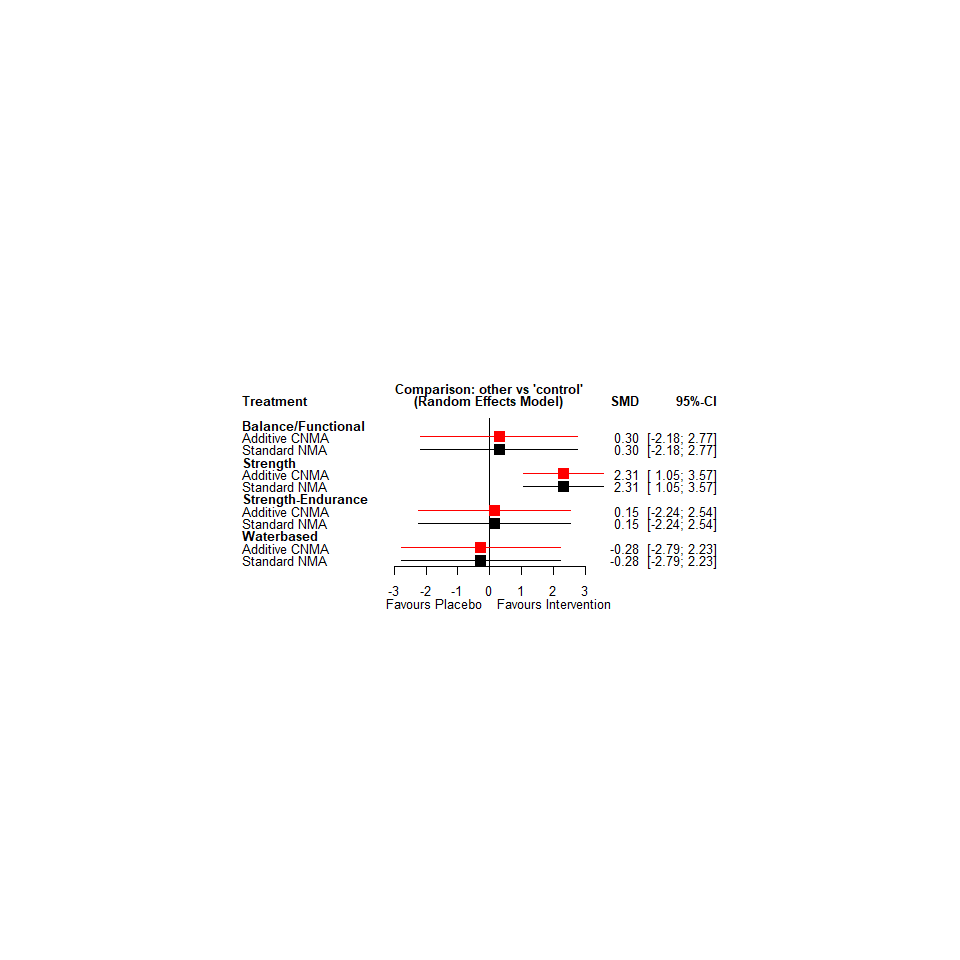


Supplementary Figure S12 Forest plot: Effects of physical activity interventions on dynamic balance

## Proactive balance


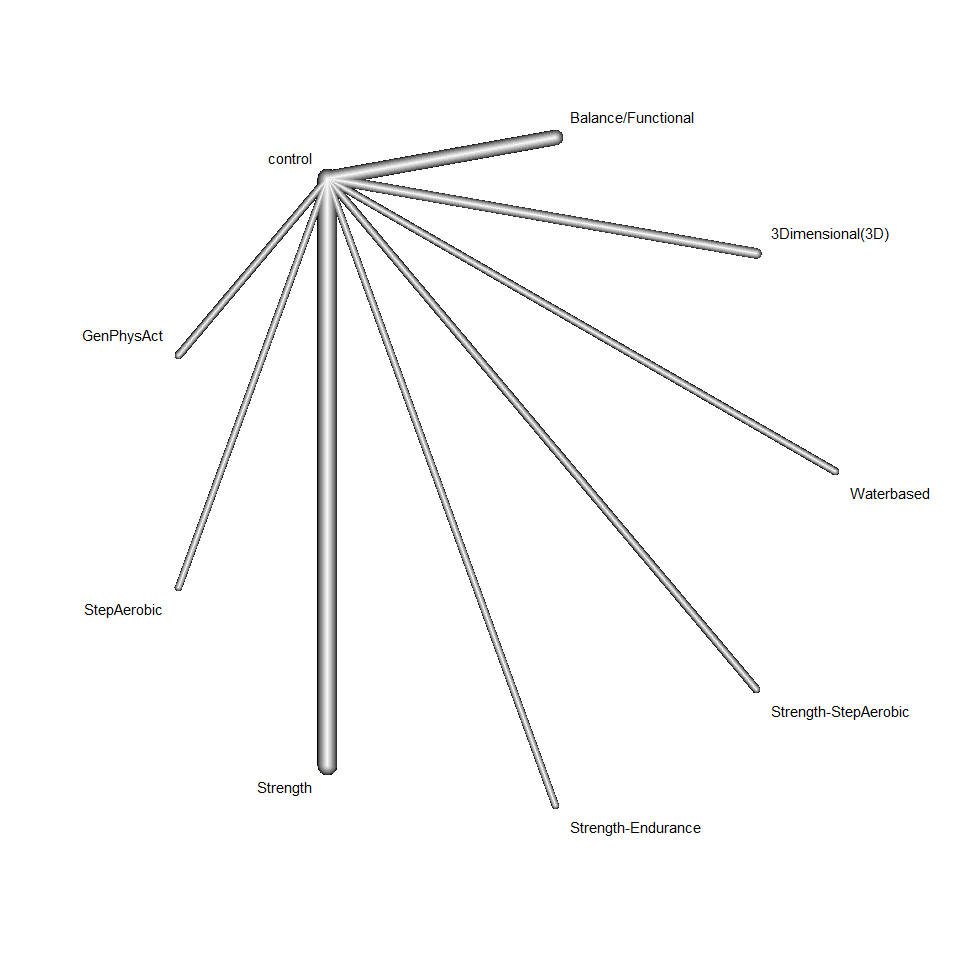


Supplementary Figure S13 Network of studies measuring proactive Balance. GenPhysAct: General Physical Activity.

Number of studies: k = 17
Number of pairwise comparisons: m = 17
Number of treatments: n = 9
Number of active components: c = 6
Number of designs: d = 8

Results for combinations (additive model, random effects model):
 SMD 95%-CI z p-value
3Dimensional (3D) 1.9772 [ 0.9696, 2.9848] 3.85 0.0001
Step aerobic 0.2057 [-0.6380; 1.0495] 0.48 0.6327
control 0.0000 [ 0.0000; 0.0000] -- --
Balance/Functional 0.5569 [-0.0016; 1.1155] 1.95 0.0507
GenPhyAct -0.2129 [-1.2312; 0.8054] -0.41 0.6819
Strength 1.5298 [ 1.0668; 1.9927] 6.48 < 0.0001
Strength-Aerobic 1.7355 [ 0.8897; 2.5814] 4.02 < 0.0001
Strength-Endurance 0.1276 [-0.8915; 1.1468] 0.25 0.8061
Waterbased 0.7572 [-0.5343; 2.0486] 1.15 0.2505


Quantifying heterogeneity / inconsistency:
tau^2 = 0.2473; tau = 0.4973; I^2 = 71.1% [46.7%; 84.4%]

Heterogeneity statistics:
 Q df p-value
Additive model 36.66 10 0.0001
Standard model 31.18 9 0.0003
Difference 3.47 1 0.0623


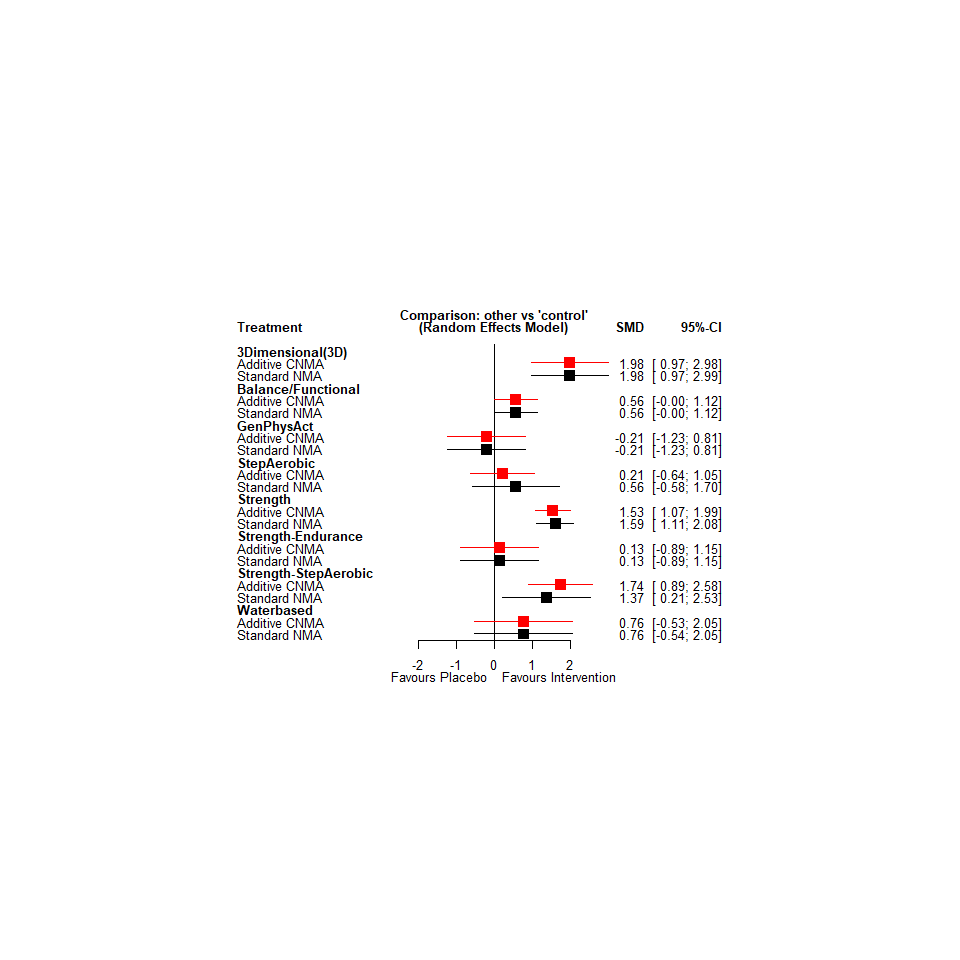


Supplementary Figure S14 Forest plot: Effects of physical activity interventions on proactive balance. GenPhysAct: General Physical Activity.

## Reactive Balance

No NMA for reactive balance conducted, since there is only one study containing that.

##

# Subgroup and sensitivity analyses

## Subgroup analyses

### Subgroup analyses for overall-strength

#### Lower-aged subgroup


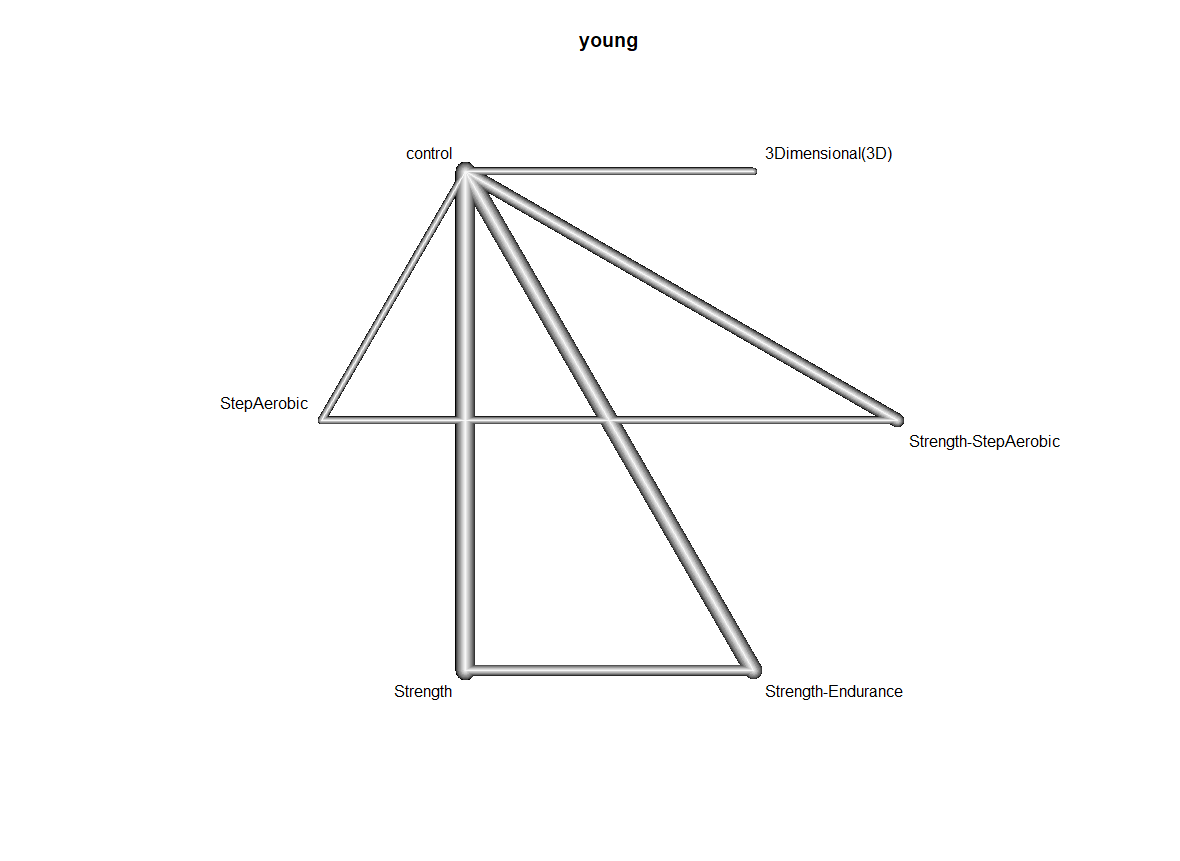


Supplementary Figure S15 Network of studies measuring overall-strength in lower-aged subgroup

Number of studies: k = 13
Number of pairwise comparisons: m = 19
Number of treatments: n = 6
Number of active components: c = 4
Number of designs: d = 6

Results for combinations (additive model, random effects model):
 SMD 95%-CI z p-value
Step aerobic -0.4989 [-1.1626; 0.1647] -1.47 0.1406
control 0.0000 [ 0.0000; 0.0000] -- --
3-Dimensional 0.7907 [-0.2940; 1.8753] 1.43 0.1531
Strength 1.0351 [ 0.6401; 1.4301] 5.14 < 0.0001
Strength-Aerobic 0.5362 [-0.0604; 1.1327] 1.76 0.0782
Strength-Endurance 1.0760 [ 0.5620; 1.5900] 4.10 < 0.0001

Quantifying heterogeneity / inconsistency:
tau^2 = 0.1779; tau = 0.4217; I^2 = 57.1% [20.4%; 76.9%]

Heterogeneity statistics:
 Q df p-value
Additive model 27.98 12 0.0056
Standard model 16.94 11 0.1097
Difference 11.04 1 0.0009

#### Higher-aged subgroup


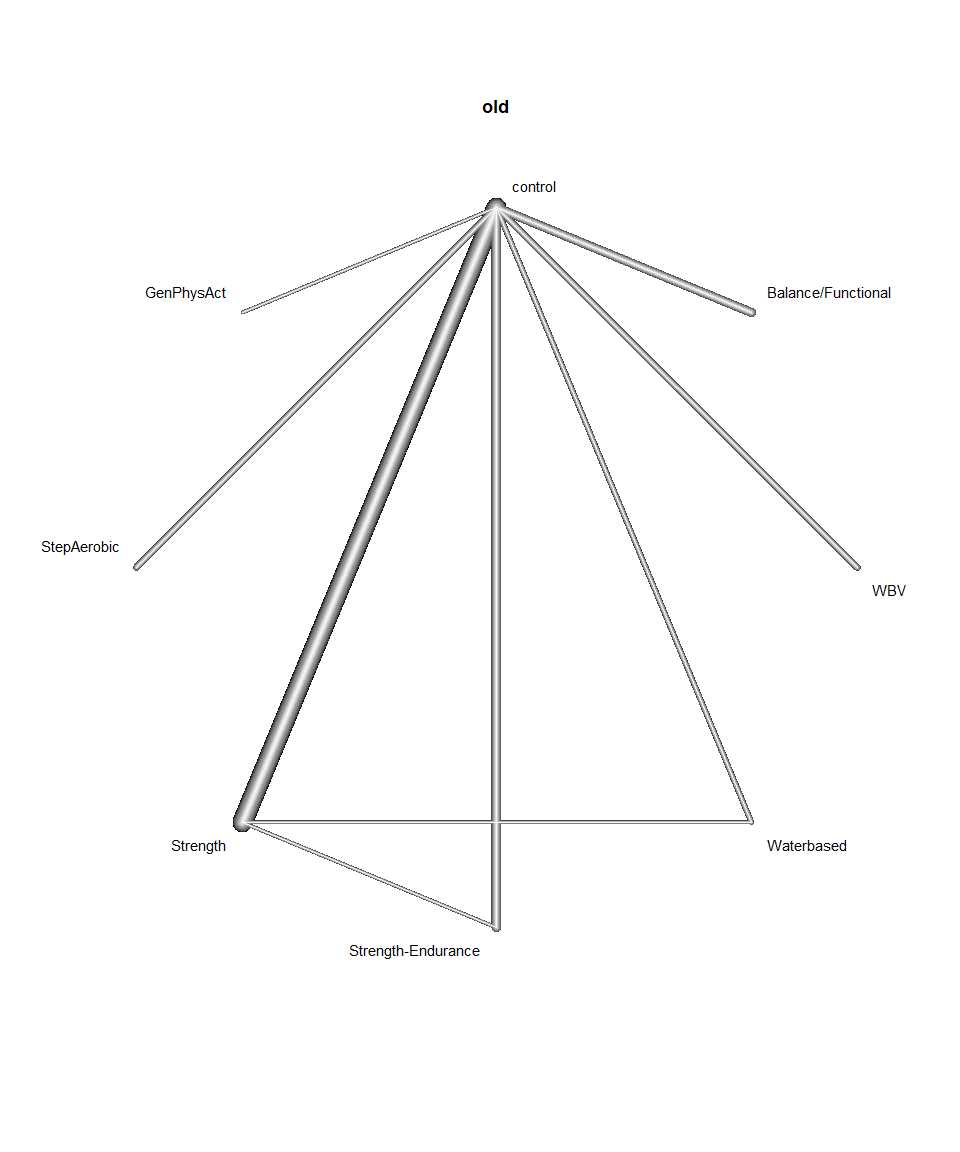


Supplementary Figure S16 Network of studies measuring overall-strength in higher-aged subgroup. GenPhysAct: General Physical Activity. WBV: Whole Body Vibration.

Number of studies: k = 39
Number of pairwise comparisons: m = 43
Number of treatments: n = 8
Number of active components: c = 7
Number of designs: d = 9

Results for combinations (additive model, random effects model):
 SMD 95%-CI z p-value
Step aerobic 1.3338 [ 0.4740; 2.1935] 3.04 0.0024
control 0.0000 [ 0.0000; 0.0000] -- --
Balance/Functional 0.5740 [-0.0447; 1.1926] 1.82 0.0690
GenPhyAct -0.0395 [-1.4181; 1.3390] -0.06 0.9552
Strength 1.173 [ 0.7695; 1.4650] 6.30 < 0.0001
Strength-Endurance 0.8046 [ 0.2193; 1.3900] 2.69 0.0071
WBV 0.4675 [-0.4398; 1.3748] 1.01 0.3125
Waterbased 1.1125 [ 0.0715; 2.1535] 2.09 0.0362

Quantifying heterogeneity / inconsistency:
tau^2 = 0.4722; tau = 0.6872; I^2 = 80.6% [73.6%; 85.7%]

Heterogeneity statistics:
 Q df p-value
Additive model 175.03 34 0
Standard model 175.03 34 < 0.0001
Difference 0.00 0 --

#### Male populations


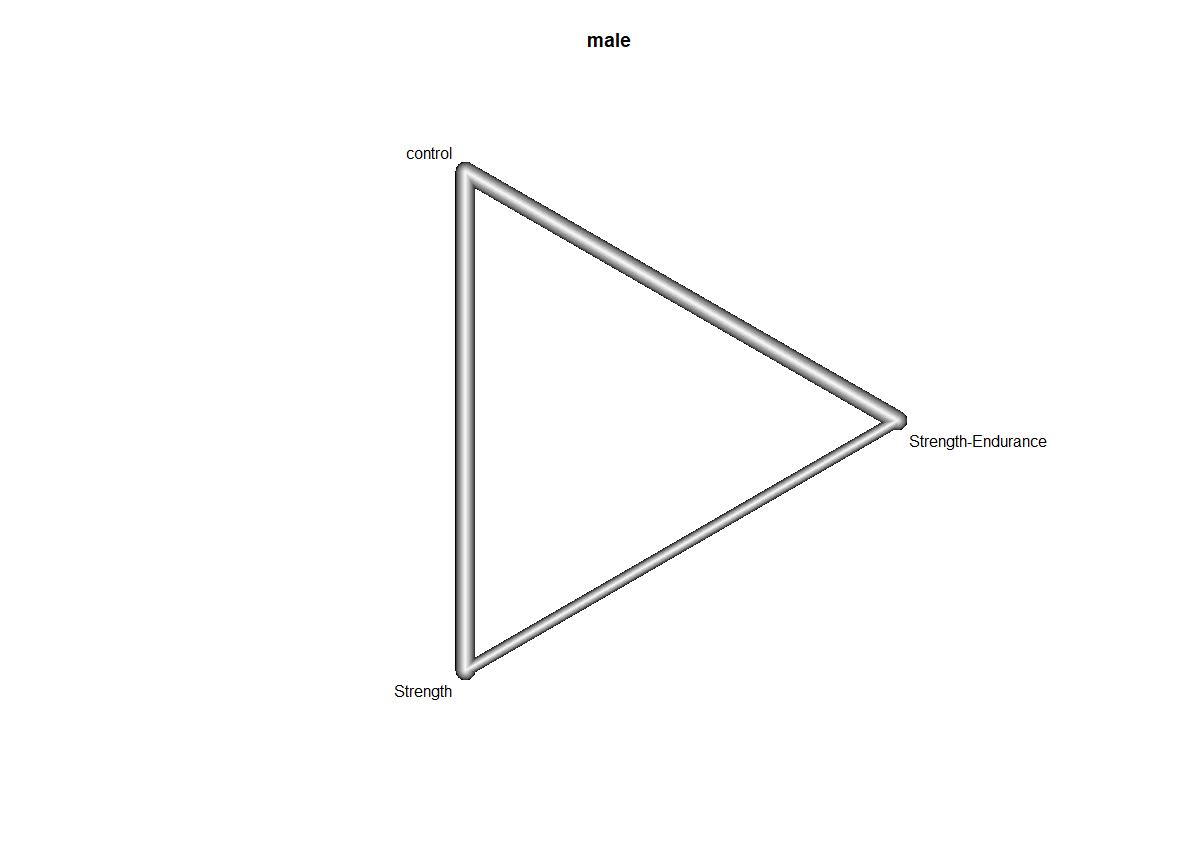


Supplementary Figure S17 Network of studies measuring overall-strength in male populations

Number of studies: k = 11
Number of pairwise comparisons: m = 17
Number of treatments: n = 3
Number of active components: c = 2
Number of designs: d = 3

Results for combinations (additive model, random effects model):
 SMD 95%-CI z p-value
control 0.0000 [0.0000; 0.0000] -- --
Strength 1.3395 [0.8879; 1.7912] 5.81 < 0.0001
Strength-Endurance 1.0060 [0.5464; 1.4656] 4.29 < 0.0001

Quantifying heterogeneity / inconsistency:
tau^2 = 0.2472; tau = 0.4972; I^2 = 63.7% [34.2%; 80.0 %]

Heterogeneity statistics:

Q df p-value
Additive model 33.09 12 0.0009
Standard model 33.09 12 0.0009
Difference 0.00 0 --

#### Female populations


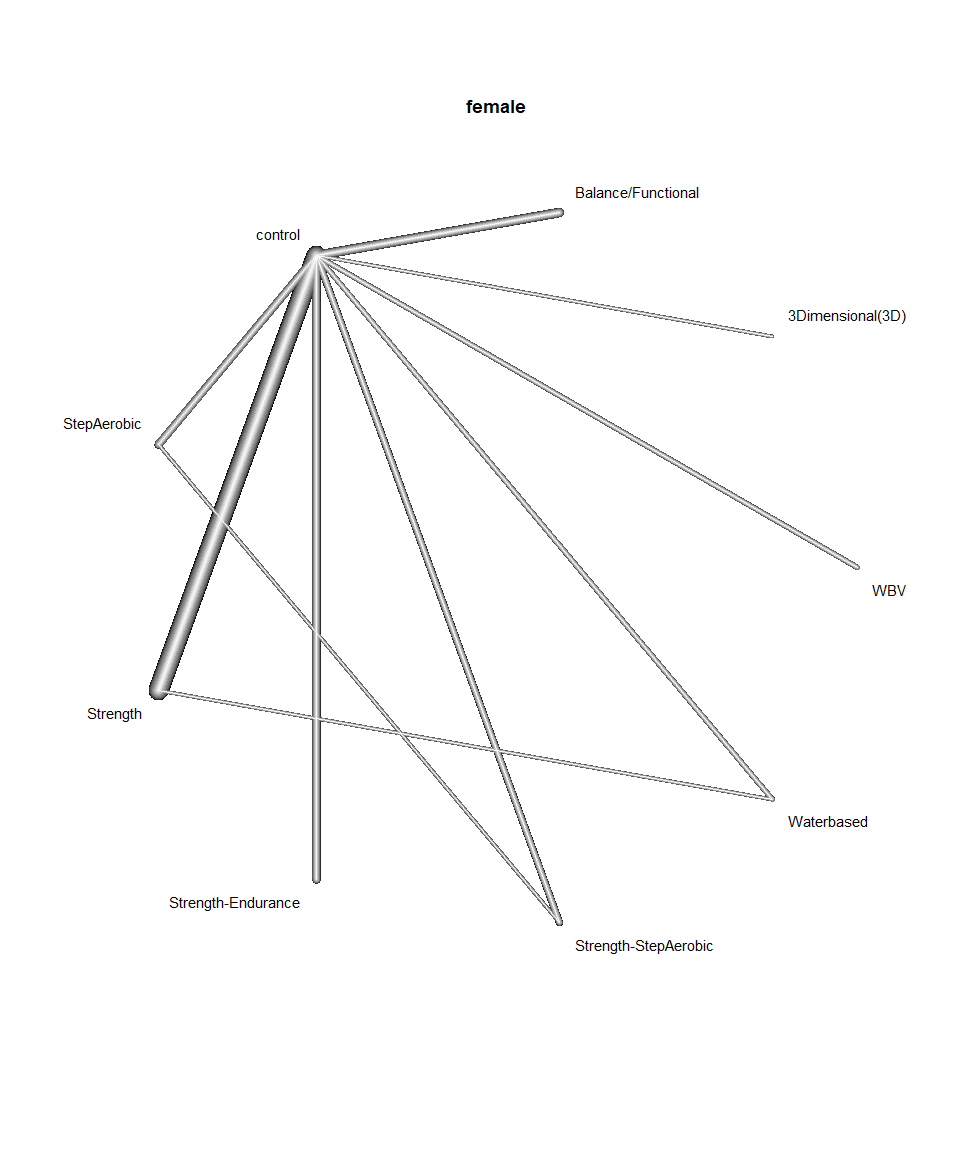


Supplementary Figure S18 Network of studies measuring overall-strength in female populations

Number of studies: k = 37
Number of pairwise comparisons: m = 41
Number of treatments: n = 9
Number of active components: c = 7
Number of designs: d = 10

Results for combinations (additive model, random effects model):
 SMD 95%-CI z p-value
Step aerobic 0.4305 [-0.2283; 1.0892] 1.28 0.2003
control 0.0000 [ 0.0000; 0.0000] -- --
Balance/Functional 0.6160 [-0.1106; 1.3427] 1.66 0.0966
3Dimensional (3D) 0.7907 [-0.8370; 2.4183] 0.95 0.3410
Strength 0.9316 [ 0.5626; 1.3006] 4.95 < 0.0001
Strength-Aerobic 1.3621 [ 0.6846; 2.0396] 3.94 < 0.0001
Strength-Endurance 0.9162 [ 0.1240; 1.7084] 2.27 0.0234
WBV 0.3302 [-0.8376; 1.4981] 0.55 0.5794
Waterbased 1.0512 [-0.0590; 2.1613] 1.86 0.0635

Quantifying heterogeneity / inconsistency:
tau^2 = 0.5613; tau = 0.7492; I^2 = 83.5% [77.8%; 87.8%]

Heterogeneity statistics:
 Q df p-value
Additive model 194.25 32 0
Standard model 162.05 31 < 0.0001
Difference 32.19 1 < 0.0001

#### Higher-aged women


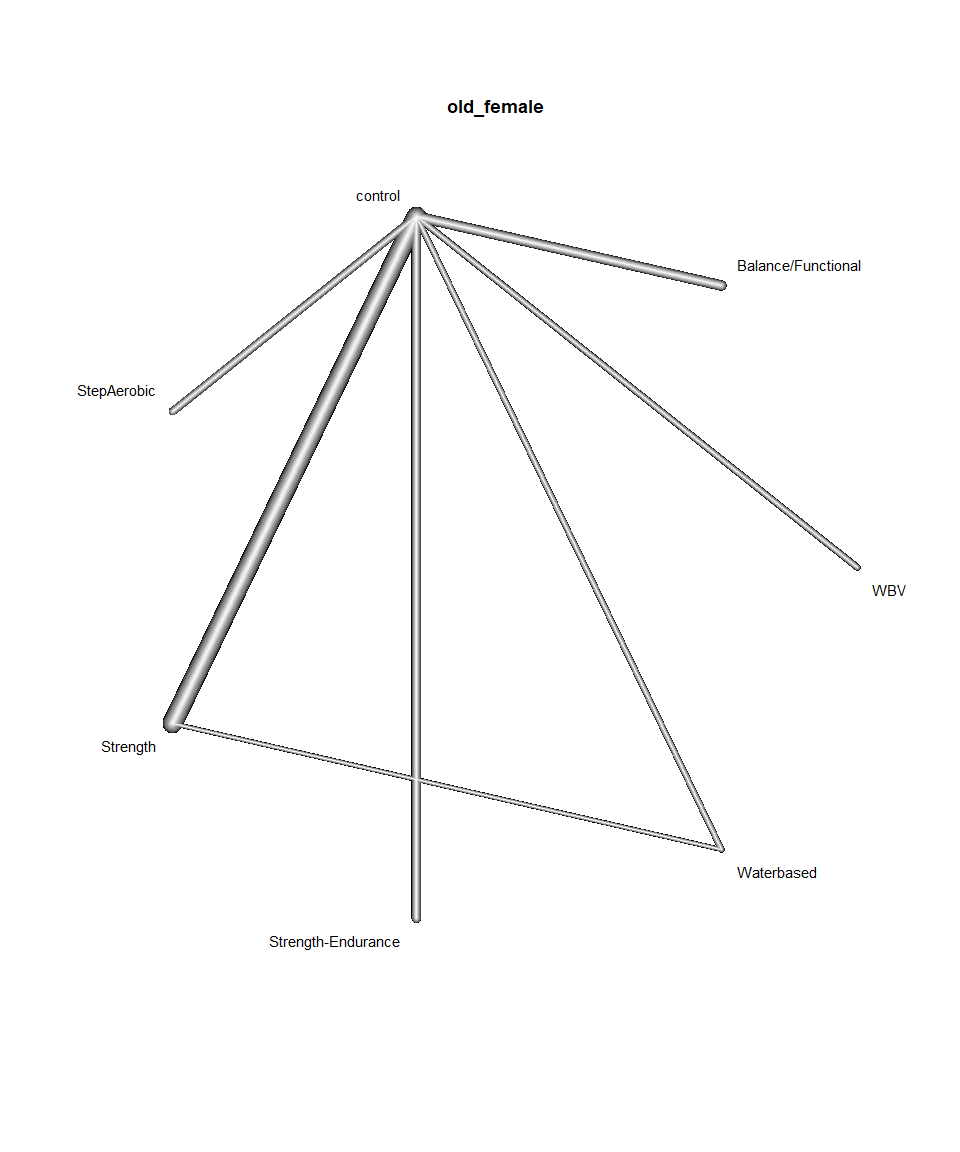


Supplementary Figure S19 Network of studies measuring overall-strength in the higher-aged subgroup of women. WBV: Whole Body Vibration.

Number of studies: k = 31
Number of pairwise comparisons: m = 33
Number of treatments: n = 7
Number of active components: c = 6
Number of designs: d = 7

Results for combinations (additive model, random effects model):
 SMD 95%-CI z p-value
Step aerobic 1.3429 [ 0.4185; 2.2673] 2.85 0.0044
control 0.0000 [ 0.0000; 0.0000] -- --
Balance/Functional 0.6160 [-0.1110; 1.3430] 1.66 0.0968
Strength 1.1367 [ 0.7171; 1.5564] 5.31 < 0.0001
Strength-Endurance 0.9163 [ 0.1236; 1.7089] 2.27 0.0235
WBV 0.3302 [-0.8382; 1.4986] 0.55 0.5796
Waterbased 1.1202 [ 0.0075; 2.2328] 1.97 0.0485

Quantifying heterogeneity / inconsistency:
tau^2 = 0.5619; tau = 0.7496; I^2 = 83.1% [76.4%; 87.9%]

Heterogeneity statistics:
 Q df p-value
Additive model 154.02 26 0
Standard model 154.02 26 < 0.0001
Difference 0.00 0 --

#### Inactive populations


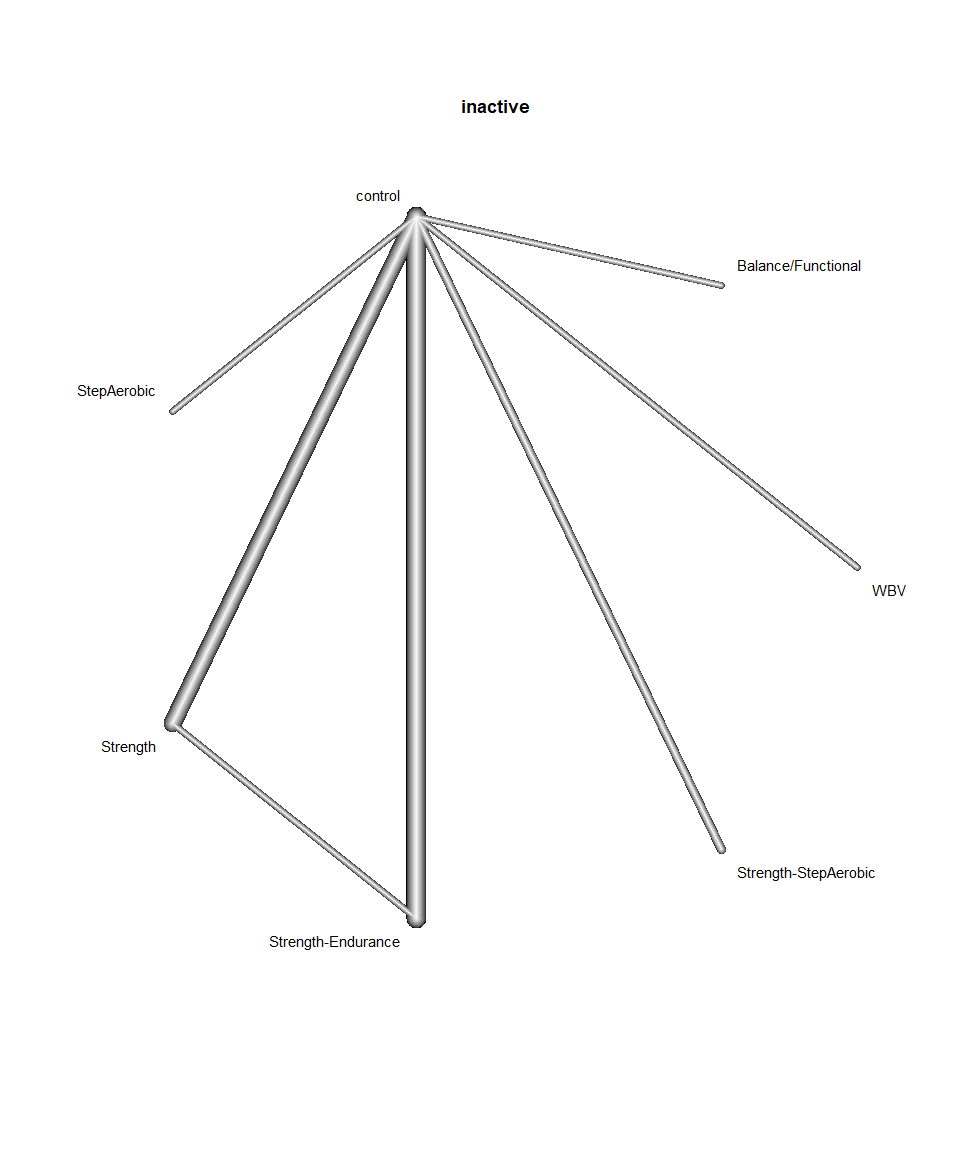


Supplementary Figure S20 Network of studies measuring overall-strength in inactive populations. WBV: Whole Body Vibration.

Number of studies: k = 17
Number of pairwise comparisons: m = 19
Number of treatments: n = 7
Number of active components: c = 5
Number of designs: d = 7

Results for combinations (additive model, random effects model):
 SMD 95%-CI z p-value
Step aerobic -0.2978 [-1.1783; 0.5826] -0.66 0.5073
control 0.0000 [ 0.0000; 0.0000] -- --
Balance/Functional 0.8255 [-0.5635; 2.2144] 1.16 0.2441
Strength 0.9670 [ 0.3920; 1.5420] 3.30 0.0010
Strength-Aerobic 0.6691 [-0.1599; 1.4981] 1.58 0.1136
Strength-Endurance 1.1265 [ 0.5823; 1.6708] 4.06 < 0.0001
WBV 0.5845 [-0.8871; 2.0561] 0.78 0.4363

Quantifying heterogeneity / inconsistency:
tau^2 = 0.3955; tau = 0.6289; I^2 = 72.3% [52.6%; 83.8%]

Heterogeneity statistics:
 Q df p-value
Additive model 46.94 13 < 0.0001
Standard model 42.55 12 < 0.0001
Difference 4.38 1 0.0363

#### Inactive higher-aged women


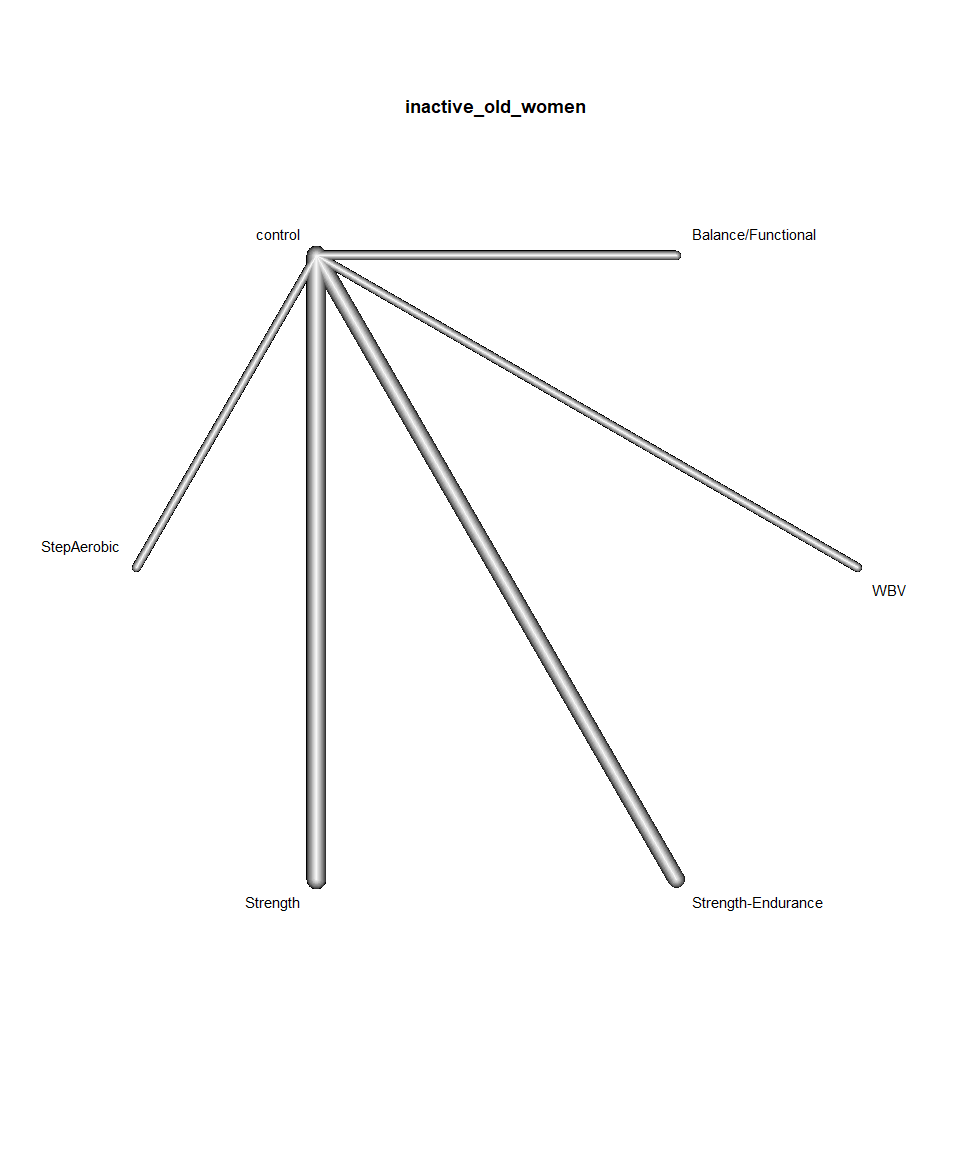


Supplementary Figure S21 Network of studies measuring overall-strength in inactive women in the higher-aged subgroup. WBV: Whole Body Vibration.

Number of studies: k = 10
Number of pairwise comparisons: m = 10
Number of treatments: n = 6
Number of active components: c = 5
Number of designs: d = 5

Results for combinations (additive model, random effects model):
 SMD 95%-CI z p-value
Aerobic 0.1247 [-1.4368; 1.6863] 0.16 0.8756
control 0.0000 [ 0.0000; 0.0000] -- --
Balance/Functional 0.8255 [-0.7920; 2.4429] 1.00 0.3172
Strength 0.6522 [-0.2172; 1.5216] 1.47 0.1415
Strength-Endurance 1.0603 [ 0.1182; 2.0024] 2.21 0.0274
WBV 0.5845 [-1.1044; 2.2734] 0.68 0.4976

Quantifying heterogeneity / inconsistency:
tau^2 = 0.5743; tau = 0.7578; I^2 = 79.1% [54.2%; 90.4%]

Heterogeneity statistics:
 Q df p-value
Additive model 23.90 5 0.0002
Standard model 23.90 5 0.0002
Difference 0.00 0 --

### Subgroup analyses for postural balance

#### Lower-aged subgroup


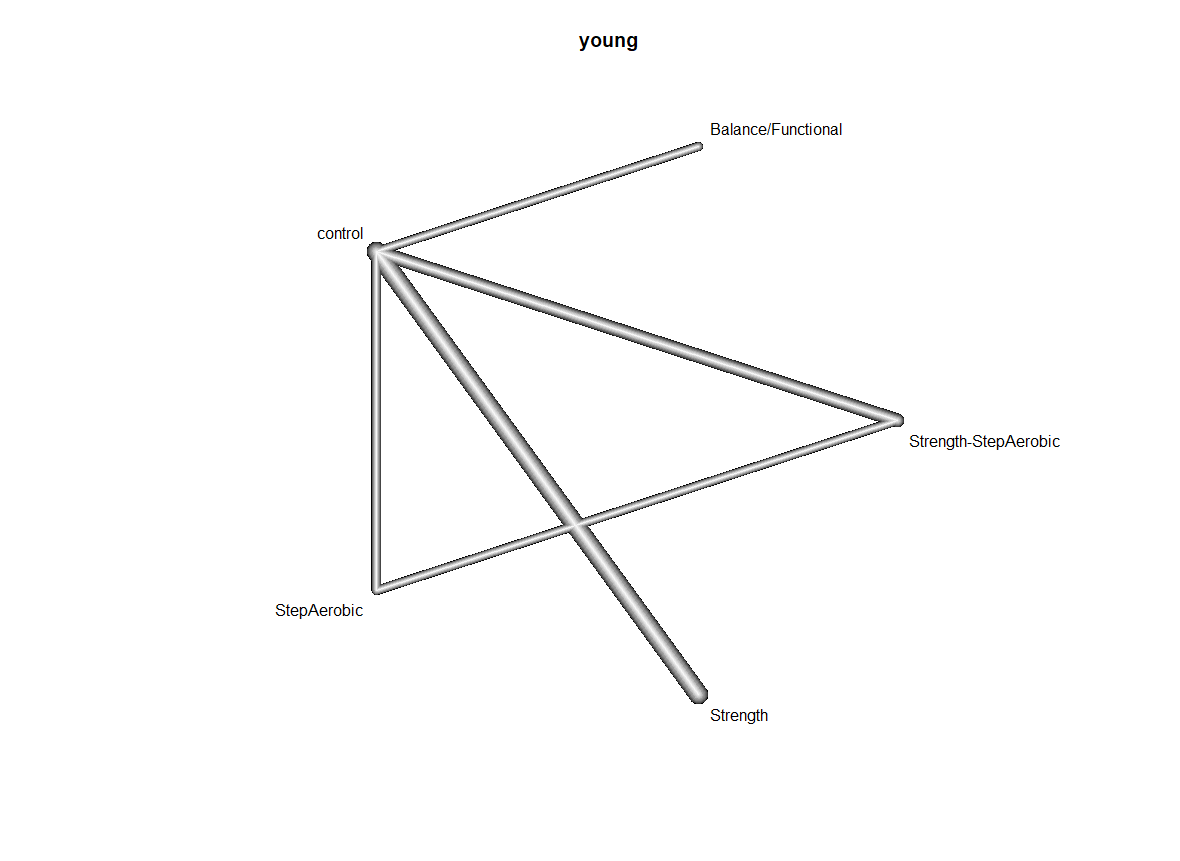


Supplementary Figure S22 of studies measuring overall-balance in the lower-aged subgroup

Number of studies: k = 7
Number of pairwise comparisons: m = 9
Number of treatments: n = 4
Number of active components: c = 3
Number of designs: d = 4

Results for combinations (additive model, random effects model):
 SMD 95%-CI z p-value
Balance/Functional 0.3242 [-0.9672; 1.6156] 0.49 0.6227
Step Aerobic 0.2502 [-0.7553; 1.2557] 0.49 0.6258
control 0.0000 [ 0.0000; 0.0000] -- --
Strength 0.4556 [-0.1360; 1.0472] 1.51 0.1312
Strength-Aerobic 0.7058 [-0.2277; 1.6392] 1.48 0.1384


Quantifying heterogeneity / inconsistency:
tau^2 = 0.3999; tau = 0.6324; I^2 = 86.2% [72.0%; 93.2%]

Heterogeneity statistics:
 Q df p-value
Additive model 36.15 5 < 0.0001
Standard model 36.04 4 < 0.0001
Difference 0.11 1 0.7356

#### Higher-aged subgroup


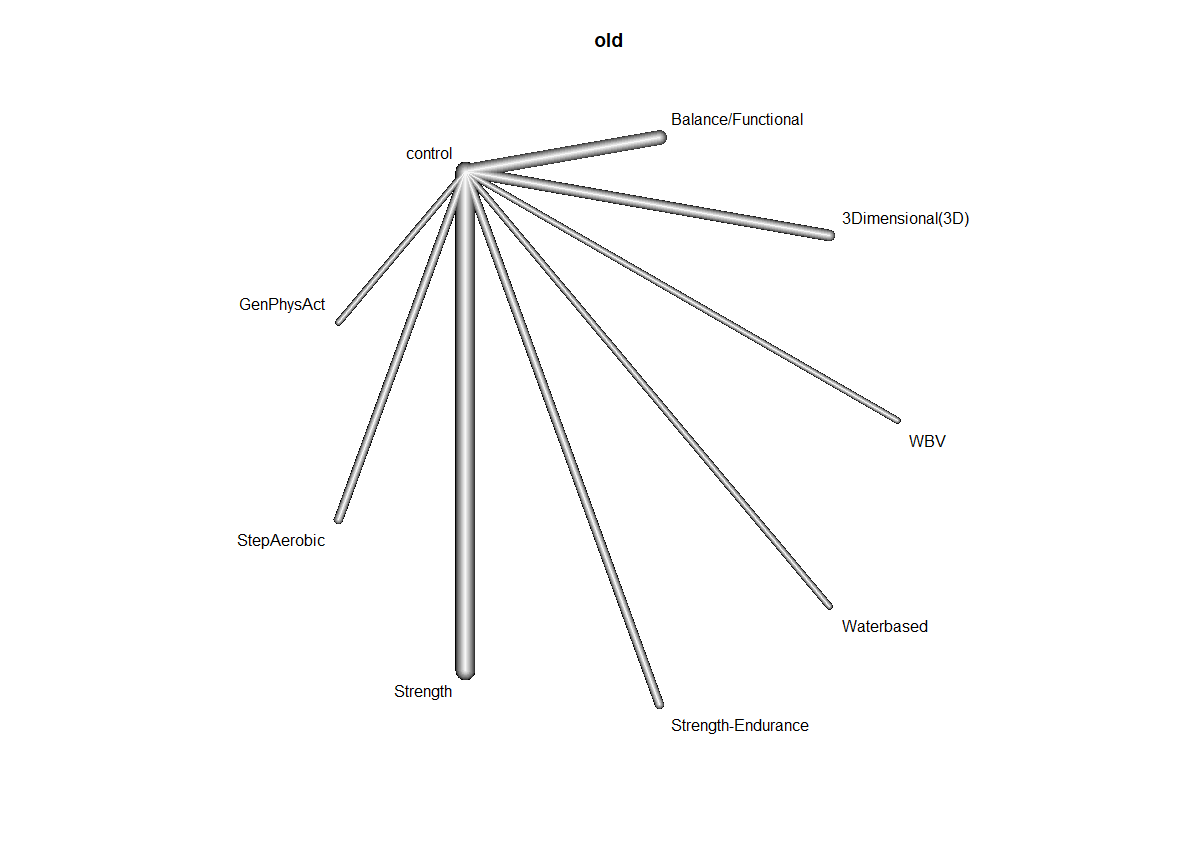


Supplementary Figure S23 Network of studies measuring overall-balance in the higher-aged subgroup. GenPhysAct: General Physical Activity. WBV: Whole Body Vibration.

Number of studies: k = 23
Number of pairwise comparisons: m = 23
Number of treatments: n = 9
Number of active components: c = 8
Number of designs: d = 8

Results for combinations (additive model, random effects model):
 SMD 95%-CI z p-value
Step aerobic -0.1032 [-1.2496; 1.0433] -0.18 0.8600
control 0.0000 [ 0.0000; 0.0000] -- --
Balance/Functional 0.5161 [-0.2092; 1.2414] 1.39 0.1631
GenPhyAct -0.2129 [-1.7563; 1.3305] -0.27 0.7869
Strength 1.6854 [ 1.0839; 2.2869] 5.49 < 0.0001
Strength-Endurance 0.1122 [-1.0457; 1.2701] 0.19 0.8493
WBV 0.1694 [-1.5033; 1.8420] 0.20 0.8427
Waterbased -0.2823 [-2.0068; 1.4422] -0.326 0.7483

Quantifying heterogeneity / inconsistency:
tau^2 = 0.5975; tau = 0.7730; I^2 = 84.6% [76.4%; 90.0%]

Heterogeneity statistics:
 Q df p-value
Additive model 97.37 15 < 0.0001
Standard model 97.37 15 < 0.0001
Difference 0.00 0 --

#### Male populations

Number of studies: k = 1

No sub analysis for male populations was conducted, since there is only one study fulfilling the criteria.

#### Female populations


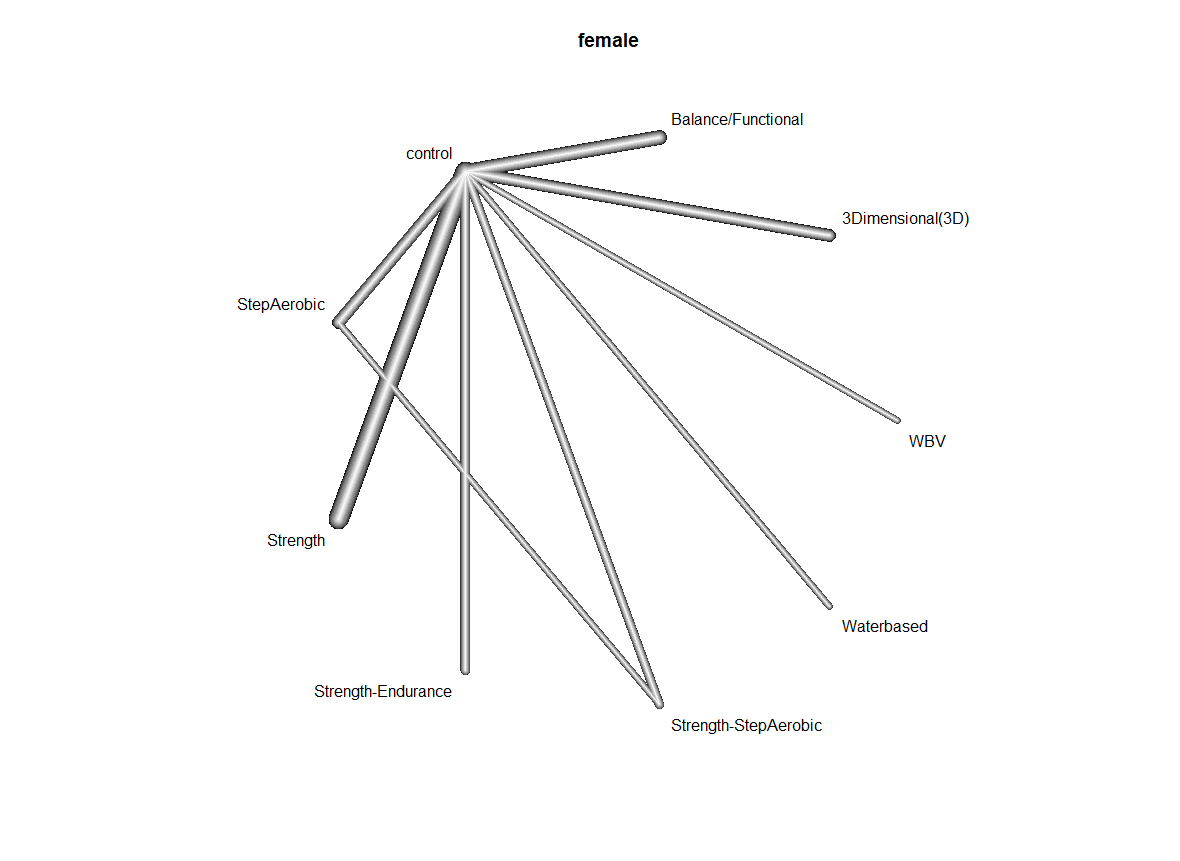


Supplementary Figure S24 Network of studies measuring overall-balance in female populations. WBV: Whole Body Vibration.

Number of studies: k = 22
Number of pairwise comparisons: m = 24
Number of treatments: n = 9
Number of active components: c = 7
Number of designs: d = 9

Results for combinations (additive model, random effects model):
 SMD 95%-CI z p-value
Step aerobic -0.0870 [-0.8216; 0.6477] -0.23 0.8165
control 0.0000 [ 0.0000; 0.0000] -- --
Balance/Functional 0.5329 [-0.1853; 1.2511] 1.45 0.1459
Strength 0.9114 [ 0.4124; 1.4104] 3.58 0.0003
Strength-Aerobic 0.8244 [ 0.0471; 1.6018] 2.08 0.0376
Strength-Endurance 0.1135 [-0.9321; 1.1591] 0.21 0.8315
WBV 0.1694 [-1.3512; 1.6899] 0.22 0.8272
Waterbased -0.2823 [-1.8597; 1.2951] -0.35 0.7257

Quantifying heterogeneity / inconsistency:
 tau^2 = 0.4711; tau = 0.6864; I^2 = 85.6% [78.4%; 90.4%]

Heterogeneity statistics:
 Q df p-value
Additive model 111.46 16 < 0.0001
standard model 110.32 15 < 0.0001
Difference 0.62 1 0.2851

Higher-aged women


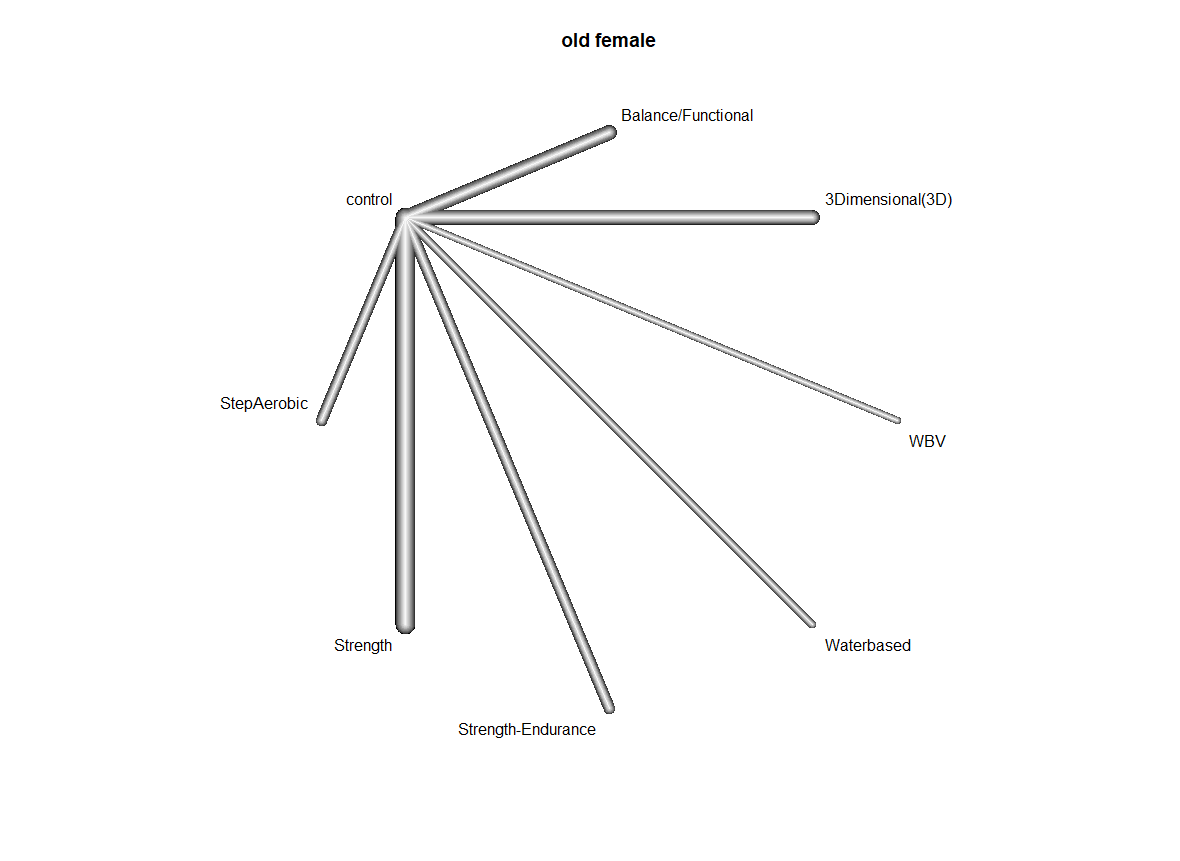


Supplementary Figure S25 Network of studies measuring overall-balance in the higher-aged subgroup of women. WBV: Whole Body Vibration.

Number of studies: k = 17
Number of pairwise comparisons: m = 17
Number of treatments: n = 8
Number of active components: c = 7
Number of designs: d = 7

Results for combinations (additive model, random effects model):
 SMD 95%-CI z p-value
Step aerobic -0.1038 [-1.1669; 0.9593] -0.19 0.8483
control 0.0000 [ 0.0000; 0.0000] -- --
Balance/Functional 0.6093 [-0.2518; 1.4704] 1.39 0.1655
Strength 1.1894 [ 0.5051; 1.8737] 3.41 0.0007
Strength-Endurance 0.1132 [-0.9607; 1.1870] 0.21 0.8364
WBV 0.1694 [-1.3892; 1.7280] 0.21 0.8313
Waterbased -0.2823 [-1.8965; 1.3318] -0.34 0.7371

Quantifying heterogeneity / inconsistency:
tau^2 = 0.5016; tau = 0.7082; I^2 = 83.7% [72.3%; 90.4%]

Heterogeneity statistics:
 Q df p-value
Additive model 61.33 10 < 0.0001
Standard model 61.33 10 < 0.0001
Difference 0.00 0

#### Inactive populations


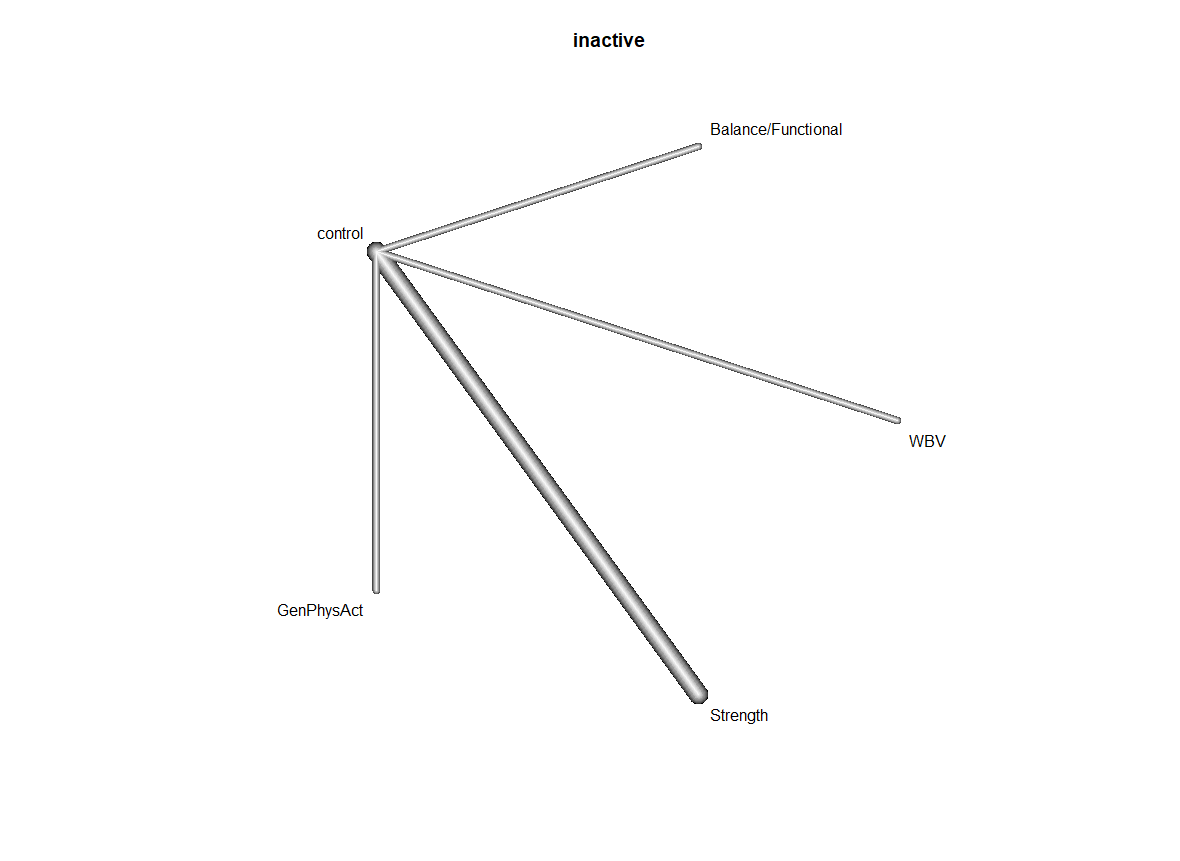


Supplementary Figure S26 Network of studies measuring overall-balance in inactive populations. WBV: Whole Body Vibration.

Number of studies: k = 19
Number of pairwise comparisons: m = 19
Number of treatments: n = 6
Number of active components: c = 5
Number of designs: d = 5

Results for combinations (additive model, random effects model):
 SMD 95%-CI z p-value
control 0.0000 [0.0000; 0.0000] -- --
Balance/Functional 0.4750 [-0.3549; 1.3049] 1.12 0.2619
Strength 1.3269 [0.6935; 1.9604] 4.11 < 0.0001
Step aerobic -1.1022 [-1.4215; 1.2170] -0.15 0.8793
Strength-endurance 0.1108 [-1.2203; 1.4419] 0.16 0.8704

Quantifying heterogeneity / inconsistency:
tau^2 = 0.8193; tau = 0.9052; I^2 = 91.1% [87.0%; 93.9%]

Heterogeneity statistics:
 Q df p-value
Additive model 157.19 14 --
Standard model 157.19 14 < 0.0001
Difference 0.00 0 --

####

#### Inactive higher-aged women


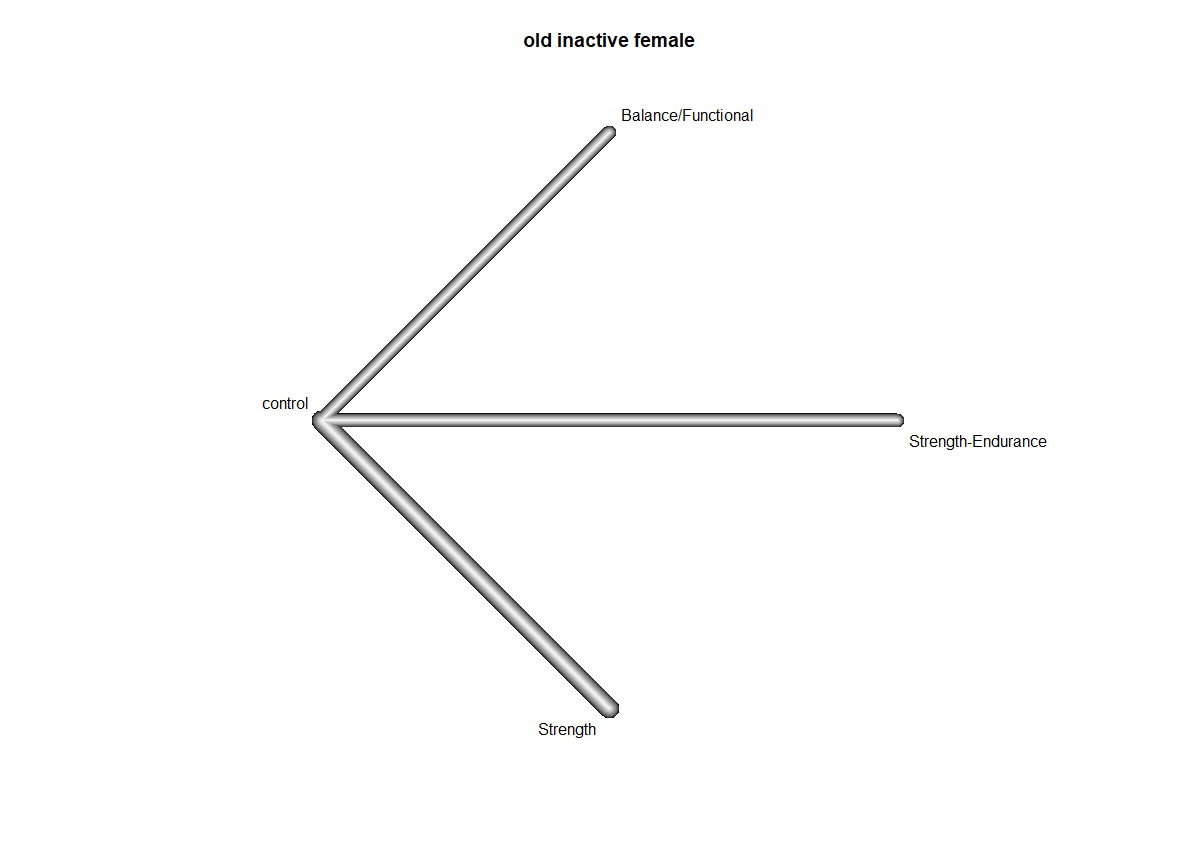


Supplementary Figure S27 Network of studies measuring overall-balance in inactive higher-aged women

Number of studies: k = 2
Number of pairwise comparisons: m = 2
Number of treatments: n = 3
Number of active components: c = 2
Number of designs: d = 2

Results for combinations (additive model, random effects model):
 SMD 95%-CI z p-value
control 0.0000 [0.0000; 0.0000] -- --
Balance/Functional 1.2723 [0.6566; 1.8879] 4.05 < 0.0001
Strength 0.8089 [0.1532; 1.4645] 2.42 0.0156

Quantifying heterogeneity / inconsistency:
tau^2 = NA; tau = NA

Heterogeneity statistics:
 Q df p-value
Additive model 0.00 0 --
Standard model 0.00 0 --
Difference 0.00 0 --

# Sensitivity analyses

## PEDRO Score of 6 or higher

### Overall-strength

Number of studies: k = 14
Number of pairwise comparisons: m = 16
Number of treatments: n = 5
Number of active components: c = 4
Number of designs: d = 5

Results for combinations (additive model, random effects model):
 SMD 95%-CI z p-value
control 0.0000 [ 0.0000; 0.0000] -- --
Balance/Functional 0.5652 [-0.2779; 1.4084] 1.31 0.1889
Strength 0.9966 [ 0.4479; 1.5454] 3.56 0.0004
Strength-Endurance 0.5371 [-0.1300; 1.2042] 1.58 0.1145
WBV 0.5845 [-0.9373; 2.1063] 0.75 0.4516


Quantifying heterogeneity / inconsistency:
tau^2 = 0.4345; tau = 0.6592; I^2 = 84.5% [74.4%; 90.6%]

Heterogeneity statistics:
 Q df p-value
Additive model 70.85 11 < 0.0001
Standard model 70.85 11 < 0.0001
Difference 0.00 0 --


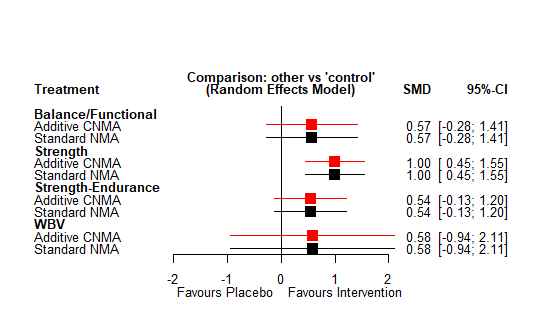


Supplementary Figure 28 Forest plot: Effects of physical activity interventions on overall-strength in high-quality studies. WBV: Whole Body Vibration.

### Overall-balance

Number of studies: k = 7
Number of pairwise comparisons: m = 7
Number of treatments: n = 4
Number of active components: c = 3
Number of designs: d = 3

Results for combinations (additive model, random effects model):
 SMD 95%-CI z p-value
control 0.0000 [ 0.0000; 0.0000] -- --
Balance/Functional 0.4849 [ 0.1334; 0.8363] 2.70 0.0068
Strength 0.4783 [-0.3994; 1.3560] 1.07 0.2855
Strength-Endurance 0.1542 [-0.5183; 0.8266] 0.45 0.6531

Quantifying heterogeneity / inconsistency:
tau^2 = 0.0947; tau = 0.3077; I^2 = 60% [0 %; 85.1%]

Heterogeneity statistics:
 Q df p-value
Additive model 10.01 4 0.0402
Standard model 10.01 4 0.0402
Difference 0.00 0 --


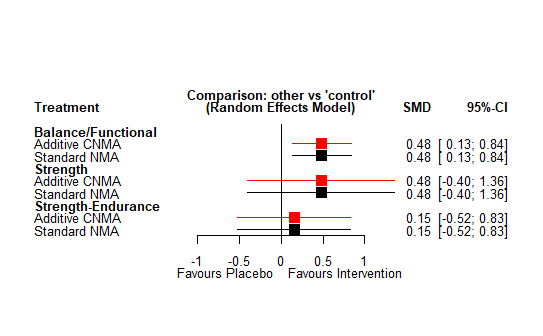


Supplementary Figure S29 Forest plot: Effects of physical activity interventions on overall-balance in high quality studies

### Subdomains of muscle strength

#### Maximum strength

Number of studies: k = 14
Number of pairwise comparisons: m = 16
Number of treatments: n = 5
Number of active components: c = 4
Number of designs: d = 5

Results for combinations (additive model, random effects model):
 SMD 95%-CI z p-value
control 0.0000 [ 0.0000; 0.0000] -- --
Balance/Functional 0.4434 [-0.5042; 1.3911] 0.92 0.3591
Strength 1.1584 [ 0.5407; 1.7760] 3.68 0.0002
Strength-Endurance 0.7901 [ 0.0287; 1.5516] 2.03 0.0420
WBV 0.5845 [-1.1096; 2.2786] 0.68 0.4989

Quantifying heterogeneity / inconsistency:
tau^2 = 0.5788; tau = 0.7608; I^2 = 87.6% [80.2%; 92.2%]

Heterogeneity statistics:
 Q df p-value
Additive model 88.76 11 < 0.0001
Standard model 88.76 11 < 0.0001
Difference 0.00 0 --

####


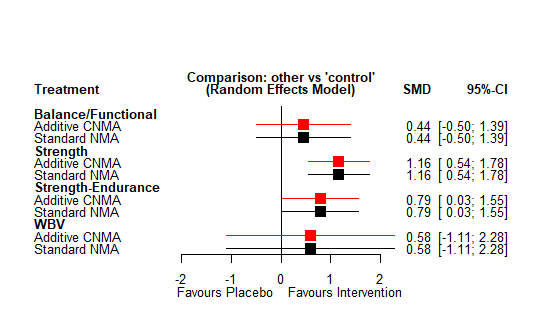


Supplementary Figure S30 Forest plot: Effects of physical activity interventions on maximum strength in high-quality studies. WBV: Whole Body Vibration.

#### Muscle Power

Number of studies: k = 2
Number of pairwise comparisons: m = 4
Number of treatments: n = 4
Number of active components: c = 3
Number of designs: d = 2

Results for combinations (additive model, random effects model):
 SMD 95%-CI z p-value
control 0.0000 [ 0.0000; 0.0000] -- --
Balance/Functional 0.4725 [ 0.0161; 0.9288] 2.03 0.0424
Strength 0.5895 [-0.0521; 1.2311] 1.80 0.0717
Strength-Endurance 0.5898 [-0.0299; 1.2094] 1.87 0.0621


Quantifying heterogeneity / inconsistency:
tau^2 = NA; tau = NA

Heterogeneity statistics:
 Q df p-value
Additive model 0.00 0 --
Standard model 0.00 0 --
Difference 0.00 0 --

####


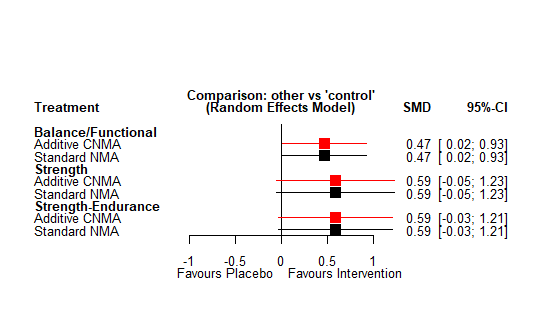


Supplementary Figure S31 Forest plot: Effects of physical activity interventions on muscle power in high quality studies

#### Strength-endurance

No PEDRO-sub analysis for strength-endurance was conducted, since there is no study containing that.

### Subdomains of postural balance

#### Static balance

Number of studies: k = 4
Number of pairwise comparisons: m = 4
Number of treatments: n = 3
Number of active components: c = 2
Number of designs: d = 2

Results for combinations (additive model, random effects model):
 SMD 95%-CI z p-value
control 0.0000 [ 0.0000; 0.0000] -- --
Balance/Functional 0.2039 [-0.0478; 0.4556] 1.59 0.1123
Strength 0.4783 [-0.1595; 1.1160] 1.47 0.1416

Quantifying heterogeneity / inconsistency:
tau^2 = 0; tau = 0; I^2 = 0%

Heterogeneity statistics:
 Q df p-value
Additive model 1.22 2 0.5426
Standard model 1.22 2 0.5426
Difference 0.00 0 --


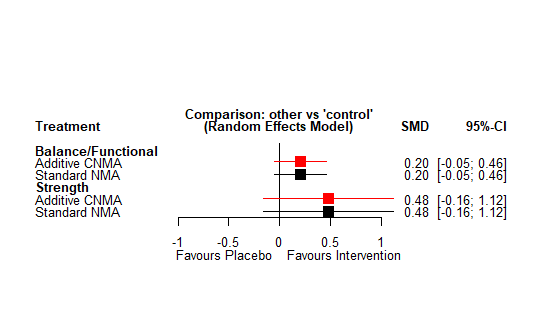


Supplementary Figure S32 Forest plot: Effects of physical activity interventions on static balance in high-quality studies

#### Dynamic balance

Number of studies: k = 1

No PEDRO-sub analysis for strength-endurance was conducted, since only one study fulfilled the criteria.

#### Proactive balance

Number of studies: k = 5
Number of pairwise comparisons: m = 5
Number of treatments: n = 3
Number of active components: c = 3
Number of designs: d = 2

Results for combinations (additive model, random effects model):
 SMD 95%-CI z p-value
control 0.0000 [ 0.0000; 0.0000] -- --
Balance/Functional 0.5498 [ 0.0678; 1.0318] 2.24 0.0254
Strength-Endurance 0.1276 [-0.7217; 0.9769] 0.29 0.7684

Quantifying heterogeneity / inconsistency:
tau^2 = 0.1647; tau = 0.4059; I^2 = 68.5% [8.6%; 89.1%]

Heterogeneity statistics:
 Q df p-value
Additive model 9.51 3 0.0232
Standard model 9.51 3 0.0232
Difference 0.00 0 --


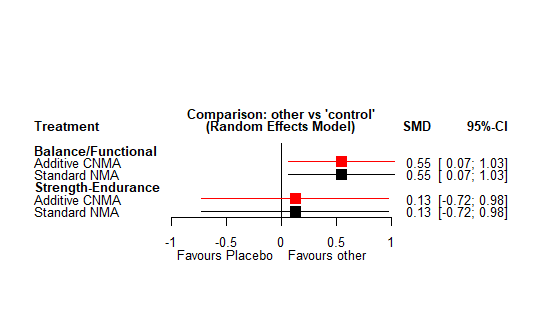


Supplementary Figure S33 Forest plot: Effects of physical activity interventions on proactive balance in high-quality studies

**Supplement 8: Physiotherapy Evidence Database (PEDro) scores of the included studies**

| *Study* | Eligibility criteria | Randomized assignation | Concealed allocation | Group homogeneity | Blinded subjects | Blinded coaches | Blinded assessors | Dropout <15% | Intention-to-treat | Group comparisons | Point and variability measures | Total PEDro score |
| --- | --- | --- | --- | --- | --- | --- | --- | --- | --- | --- | --- | --- |
| **Adams et al. (2001)** | + | + | - | + | - | - | - | + | - | + | + | 5 |
| **Almarzouki et al. (2020)** | + | + | + | + | - | - | - | + | + | + | + | 7 |
| **Anek & Bunyaratavej (2015)** | + | + | - | + | - | - | - | + | - | + | + | 5 |
| **Anek et al. (2015)** | + | + | - | + | - | - | - | - | - | + | + | 4 |
| **Araújo et al. (2015)** | + | + | - | + | - | - | - | - | - | + | - | 3 |
| **Asikainen et al. (2006)** | + | + | - | + | - | - | - | + | + | + | + | 6 |
| **Bemben et al. (2000)** | + | + | - | + |  |  |  | - | - | + | + | 4 |
| **Bolton et al. (2012)** | + | + | + | + | - | - | + | + | - | + | + | 7 |
| **Bonfante et al. (2017)** | - | + | - | + | - | - | - | - | - | + | + | 4 |
| **Colado & Triplett (2008)** | - | + | - | + | - | - | - | - | - | + | + | 4 |
| **Colado et al. (2009)** | + | + | - | + | - | - | - | - | - | + | + | 4 |
| **Conceição et al. (2013)** | + | + | - | + | - | - | - | - | - | + | + | 4 |
| **Correa et al. (2015)** | + | + | - | + | - | - | - | + | - | + | + | 5 |
| **Cotofana et al. (2010)** | - | + | - | + | - | - | + | + | - | + | + | 6 |
| **de Jong et al. (2006)** | + | + | - | + | - | - | - | - | + | + | + | 5 |
| **Deibert et al. (2011)** | + | + | - | + | - | - | - | + | + | + | + | 6 |
| **Elliot et al. (2002)** | - | + | - | + | - | - | - | - | - | + | + | 4 |
| **Figueroa et al. (2011)** | + | + | - | + | - | - | - | + | - | + | + | 5 |
| **Donges et al. (2013)** | - | + | - | + | - | - | - | + | - | + | + | 5 |
| **Duft et al. (2017)** | - | + | - | + | - | - | - | - | - | + | + | 4 |
| **Fu et al.**  **(2009)** | + | + | + | + | - | - | + | + | + | + | + | 8 |
| **Figueroa et al. (2014)** | + | + | - | + | - | - | + | + | - | + | + | 6 |
| **Flandez et al. (2016)** | + | + | - | + | - | - | - | + | - | + | + | 5 |
| **Gillett et al. (1995)** | + | + | - | - | - | - | - | + | - | + | + | 4 |
| **Granacher et al. (2011)** | + | + | - | + | - | - | - | + | - | + | + | 5 |
| **Holviala et al. (2014)** | + | + | - | + | - | - | - | + | - | + | + | 5 |
| **Janzen et al. (2006)** | - | + | - | + | - | - | + | + | - | + | + | 6 |
| **Jorgensen et al. (2011)** | + | + | - | + | - | - | - | - | + | + | + | 5 |
| **Kak et al. (2013)** | - | + | - | + | - | - | - | - | - | + | + | 4 |
| **Karatrantou et al. (2016)** | - | + | - | + | - | - | - | - | - | + | + | 4 |
| **Kim et al. (2012)** | + | + | - | + | - | - | - | - | - | + | + | 4 |
| **Klentrou et al. (2007)** | - | + | - | + | - | - | + | + | - | + | + | 6 |
| **Kloubec et al. (2010)** | - | + | - | + | - | - | - | + | - | + | + | 5 |
| **Kostić et al. (2015)** | + | + | - | + | - | - | - | + | - | + | + | 5 |
| **Libardi et al. (2012a)** | + | + | - | + | - | - | - | + | - | + | + | 5 |
| **Karavirta et al. (2011)** | - | + | - | + | - | - | - | + | + | + | + | 6 |
| **Marcus et al. (2009)** | - | + | - | + | - | - | - | + | - | + | + | 5 |
| **Marston et al. (2019)** | - | + | - | + | - | - | + | + | - | + | + | 6 |
| **Nelson et al. (1994)** | + | + | - | - | - | - | - | + | + | + | + | 5 |
| **Neves et al. (2017)** | + | + | + | + | - | - | + | - | - | + | + | 6 |
| **Nunes et al. (2017)** | + | + | - | + | - | - | - | + | - | + | + | 5 |
| **Paoli et al.(2010)** | + | + | - | - | - | - | - | + | + | + | + | 5 |
| **Reis et al. (2012)** | + | + | - | + | - | - | + | + | - | + | + | 6 |
| **Shaw et al. (2016)** | + | + | + | - | - | - | - | + | - | + | + | 5 |
| **Shirazi et al. (2006)** | + | + | - | + | - | - | - | + | - | + | + | 5 |
| **Perchthaler et al. (2015)** | - | + | - | - | - | - | - | - | - | + | + | 3 |
| **Singh et al. (2009)** | + | + | - | + | - | - | + | + | - | + | + | 6 |
| **Sipilä et al. (2001)** | + | + | + | + | - | - | - | - | - | + | + | 5 |
| **Smith et al. (2017)** | + | + | - | + | - | - | - | - | - | + | + | 4 |
| **Tsourlou et al. (2003)** | + | + | - | + | - | - | - | + | - | + | + | 5 |
| **Wong et al. (2018)** | + | + | - | + | - | - | - | + | - | + | + | 5 |
| **Wittke et al. (2017)** | - | + | + | + | - | - | + | + | + | + | + | 8 |
| **Zhao et al. (2007)** | + | + | - | + | - | - | - | - | - | + | + | 4 |
| **Taaffe et al. (2005)** | + | + | - | + | - | - | - | - | - | + | + | 4 |
| **Park et al. (2015)** | - | + | - | + | - | - | - | - | - | + | + | 4 |
| **Liphardt et al. (2015)** | - | + | - | + | - | - | - | - | - | + | + | 4 |
| **Brunelli et al. (2015)** | + | + | - | + | - | - | - | - | - | + | + | 4 |
| **Sallinen et al. (2007)** | - | + | - | + | - | - | - | + | - | + | + | 5 |
| **Uusi-Rasi et al. (2003)** | + | + | - | + | - | - | + | + | + | + | + | 7 |
| **Libardi et al. (2012b)** | - | + | - | + | - | - | - | - | - | + | + | 4 |
| **Chilibeck et al. (2013)** | + | + | + | + | - | - | + | + | + | + | + | 8 |
| **Wen et al. (2016)** | + | + | - | + | - | - | - | + | - | + | + | 5 |
| **Palumbo et al. (2012)** | + | + | - | - | - | - | - | - | - | + | + | 3 |
| **Batrakoulis et al. (2022)** | - | + | - | + | - | - | - | - | + | + | + | 5 |
| **Chasland et al. (2021)** | + | + | + | + | - | - | + | + | + | + | + | 8 |
| **Moreno-Muñoz et al. (2021)** | + | + | + | + | - | - | + | + | - | + | + | 7 |
| *TOTAL +* | 45 | 66 | 10 | 60 | 0 | 0 | 15 | 40 | 14 | 66 | 65 |  |
| *PERCENT +* | 68,18 % | 100,00 % | 15,15 % | 90,91 % | 0,00 % | 0,00 % | 22,73 % | 60,61 % | 21,21 % | 100,00 % | 98,48 % |  |

Eligibility criteria item does not contribute to total PEDro score. + indicates a ‘yes’; - indicates a ‘no’

**Supplement 9: Funnel plots**


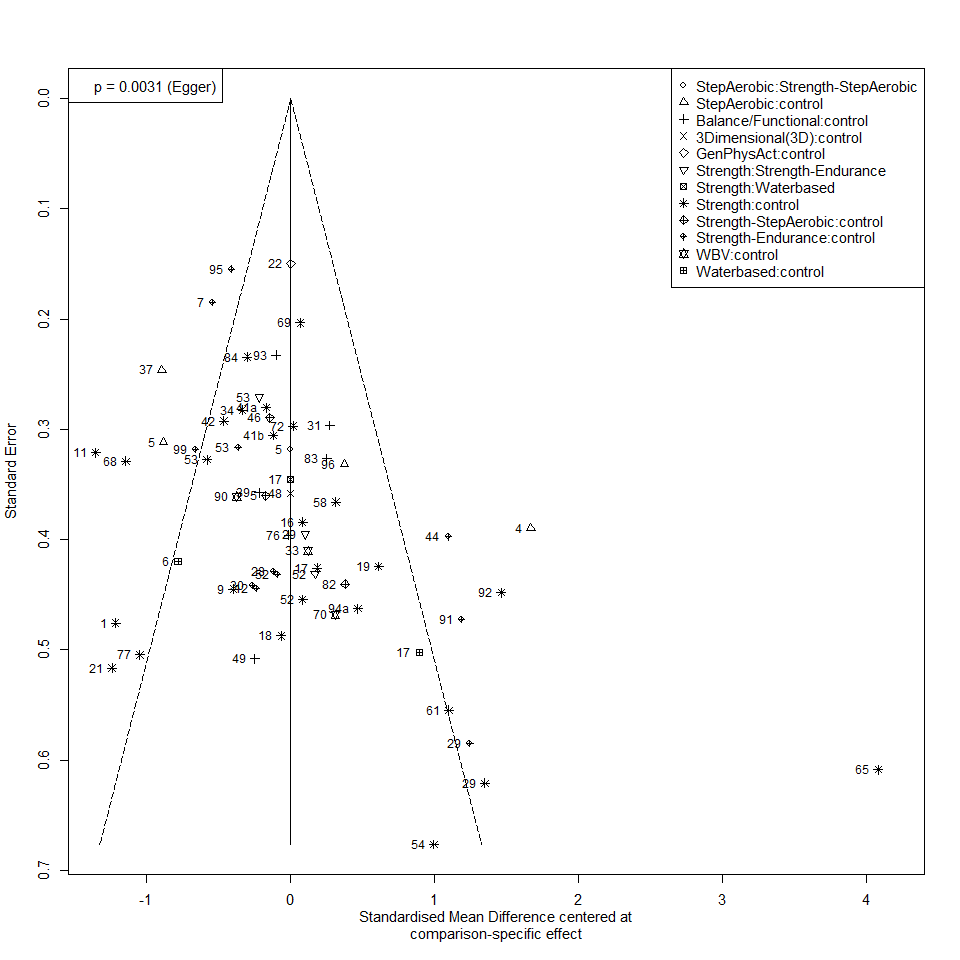


Figure F34 Funnel plot: Overall-strength


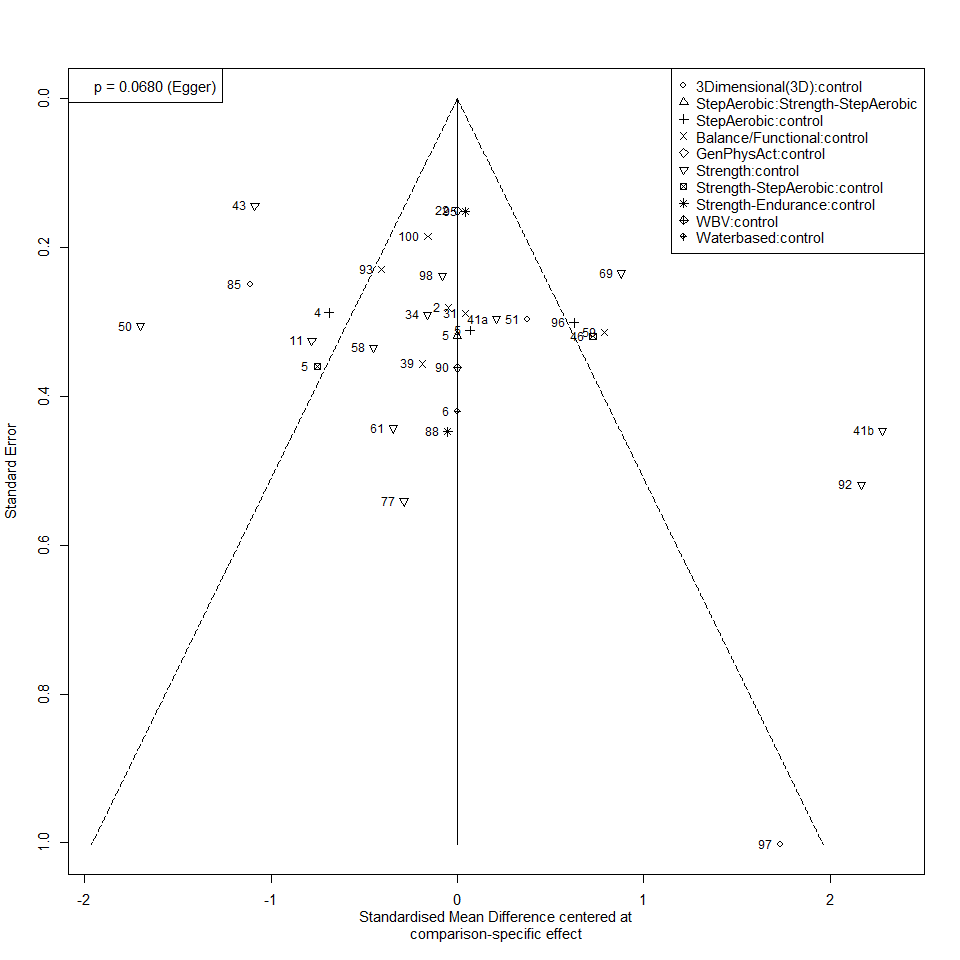


Figure F35 Funnel plot: Overall-balance

**References**

1. Orsatti FL, Maestá N, Oliveira EP de, Nahas Neto J, Burini RC, Nunes PRP, et al. Adding Soy Protein to Milk Enhances the Effect of Resistance Training on Muscle Strength in Postmenopausal Women. Journal of Dietary Supplements. 2018;15:140–52. doi:10.1080/19390211.2017.1330794.

2. Chilibeck PD, Kaviani M, Candow DG, Zello GA. Effect of creatine supplementation during resistance training on lean tissue mass and muscular strength in older adults: a meta-analysis. Open Access J Sports Med. 2017;8:213–26. doi:10.2147/OAJSM.S123529.

3. Lamb SE, Becker C, Gillespie LD, Smith JL, Finnegan S, Potter R, Pfeiffer K. Reporting of complex interventions in clinical trials: development of a taxonomy to classify and describe fall-prevention interventions. Trials. 2011;12:125. doi:10.1186/1745-6215-12-125.

4. Sherrington C, Fairhall N, Wallbank G, Tiedemann A, Michaleff ZA, Howard K, et al. Exercise for preventing falls in older people living in the community: an abridged Cochrane systematic review. Br J Sports Med. 2020;54:885–91. doi:10.1136/bjsports-2019-101512.

5. Wilson JM, Marin PJ, Rhea MR, Wilson SMC, Loenneke JP, Anderson JC. Concurrent training: a meta-analysis examining interference of aerobic and resistance exercises. Journal of Strength and Conditioning Research. 2012;26:2293–307. doi:10.1519/JSC.0b013e31823a3e2d.

6. Caetano MJD, Lord SR, Brodie MA, Schoene D, Pelicioni PHS, Sturnieks DL, Menant JC. Executive functioning, concern about falling and quadriceps strength mediate the relationship between impaired gait adaptability and fall risk in older people. Gait Posture. 2018;59:188–92. doi:10.1016/j.gaitpost.2017.10.017.

7. Ahmadiahangar A, Javadian Y, Babaei M, Heidari B, Hosseini S, Aminzadeh M. The role of quadriceps muscle strength in the development of falls in the elderly people, a cross-sectional study. Chiropr Man Therap. 2018;26:31. doi:10.1186/s12998-018-0195-x.

8. Schorderet C, Hilfiker R, Allet L. The role of the dominant leg while assessing balance performance. A systematic review and meta-analysis. Gait Posture. 2021;84:66–78. doi:10.1016/j.gaitpost.2020.11.008.

9. Choy NL, Brauer S, Nitz J. Changes in postural stability in women aged 20 to 80 years. The Journals of Gerontology Series A: Biological Sciences and Medical Sciences. 2003;58:525–30. doi:10.1093/gerona/58.6.m525.

10. Brauer SG, Burns YR, Galley P. A prospective study of laboratory and clinical measures of postural stability to predict community-dwelling fallers. The Journals of Gerontology Series A: Biological Sciences and Medical Sciences. 2000;55:M469-76. doi:10.1093/gerona/55.8.m469.

11. Maki BE, Holliday PJ, Topper AK. A prospective study of postural balance and risk of falling in an ambulatory and independent elderly population. J Gerontol. 1994;49:M72-84. doi:10.1093/geronj/49.2.m72.

12. Hilliard MJ, Martinez KM, Janssen I, Edwards B, Mille M-L, Zhang Y, Rogers MW. Lateral balance factors predict future falls in community-living older adults. Arch Phys Med Rehabil. 2008;89:1708–13. doi:10.1016/j.apmr.2008.01.023.

13. Bohannon RW. Comfortable and maximum walking speed of adults aged 20-79 years: reference values and determinants. Age Ageing. 1997;26:15–9. doi:10.1093/ageing/26.1.15.

14. Adams KJ, Swank AM, Berning JM, Sevene-Adams PG, Barnard KL, Shimp-Bowerman J. Progressive strength training in sedentary, older African American women. Medicine & Science in Sports & Exercise. 2001;33:1567–76. doi:10.1097/00005768-200109000-00021.

15. Almarzouki R, Bains G, Lohman E, Bradley B, Nelson T, Alqabbani S, et al. Improved balance in middle-aged adults after 8 weeks of a modified version of Otago Exercise Program: A randomized controlled trial. PLoS ONE. 2020;15:e0235734. doi:10.1371/journal.pone.0235734.

16. Anek A, Bunyaratavej N. Effects of Circuit Aerobic Step Exercise Program on Musculoskeletal for Prevention of Falling and Enhancement of Postural Balance in Postmenopausal Women. J Med Assoc Thai. 2015;98 Suppl 8:S88-94.

17. Anek A, Kanungsukasem V, Bunyaratavej N. Effects of Aerobic Step Combined with Resistance Training on Biochemical Bone Markers, Health-Related Physical Fitness and Balance in Working Women. J Med Assoc Thai. 2015;98 Suppl 8:S42-51.

18. Araújo JP, Neto GR, Loenneke JP, Bemben MG, Laurentino GC, Batista G, et al. The effects of water-based exercise in combination with blood flow restriction on strength and functional capacity in post-menopausal women. Age (Dordr). 2015;37:110. doi:10.1007/s11357-015-9851-4.

19. Asikainen T-M, Suni JH, Pasanen ME, Oja P, Rinne MB, Miilunpalo SI, et al. Effect of Brisk Walking in 1 or 2 Daily Bouts and Moderate Resistance Training on Lower-Extremity Muscle Strength, Balance, and Walking Performance in Women Who Recently Went Through Menopause: A Randomized, Controlled Trial. Phys Ther. 2006;86:912–23. doi:10.1093/ptj/86.7.912.

20. Batrakoulis A, Jamurtas AZ, Tsimeas P, Poulios A, Perivoliotis K, Syrou N, et al. Hybrid-type, multicomponent interval training upregulates musculoskeletal fitness of adults with overweight and obesity in a volume-dependent manner: A 1-year dose-response randomised controlled trial. Eur J Sport Sci. 2022:1–12. doi:10.1080/17461391.2021.2025434.

21. Bemben DA, Fetters NL, Bemben MG, Nabavi N, Koh ET. Musculoskeletal responses to high- and low-intensity resistance training in early postmenopausal women. Medicine & Science in Sports & Exercise. 2000;32:1949–57. doi:10.1097/00005768-200011000-00020.

22. Bolton KL, Egerton T, Wark J, Wee E, Matthews B, Kelly A, et al. Effects of exercise on bone density and falls risk factors in post-menopausal women with osteopenia: a randomised controlled trial. J Sci Med Sport. 2012;15:102–9. doi:10.1016/j.jsams.2011.08.007.

23. Bonfante ILP, Chacon-Mikahil MPT, Brunelli DT, Gáspari AF, Duft RG, Lopes WA, et al. Combined training, FNDC5/irisin levels and metabolic markers in obese men: A randomised controlled trial. Eur J Sport Sci. 2017;17:629–37. doi:10.1080/17461391.2017.1296025.

24. Brunelli D, Chacon-Mikahil M, Gáspari A, Lopes W, Bonganha V, Bonfante I, et al. Combined Training Reduces Subclinical Inflammation in Obese Middle-Age Men. Med Sci Sports Exerc. 2015;47:2207–15. doi:10.1249/MSS.0000000000000658.

25. Chasland LC, Yeap BB, Maiorana AJ, Chan YX, Maslen BA, Cooke BR, et al. Testosterone and exercise: effects on fitness, body composition, and strength in middle-to-older aged men with low-normal serum testosterone levels. Am J Physiol Heart Circ Physiol. 2021;320:H1985-H1998. doi:10.1152/ajpheart.00010.2021.

26. Chilibeck PD, Vatanparast H, Pierson R, Case A, Olatunbosun O, Whiting SJ, et al. Effect of exercise training combined with isoflavone supplementation on bone and lipids in postmenopausal women: a randomized clinical trial. J Bone Miner Res. 2013;28:780–93. doi:10.1002/jbmr.1815.

27. Colado JC, Triplett NT. Effects of a short-term resistance program using elastic bands versus weight machines for sedentary middle-aged women. Journal of Strength and Conditioning Research. 2008;22:1441–8. doi:10.1519/JSC.0b013e31817ae67a.

28. Colado JC, Triplett NT, Tella V, Saucedo P, Abellán J. Effects of aquatic resistance training on health and fitness in postmenopausal women. Eur J Appl Physiol. 2009;106:113–22. doi:10.1007/s00421-009-0996-7.

29. Conceição MS, Bonganha V, Vechin FC, Berton RPdB, Lixandrão ME, Nogueira FRD, et al. Sixteen weeks of resistance training can decrease the risk of metabolic syndrome in healthy postmenopausal women. Clin Interv Aging. 2013;8:1221–8. doi:10.2147/CIA.S44245.

30. Correa CS, Teixeira BC, Cobos RCR, Macedo RCO, Kruger RL, Carteri RBK, et al. High-volume resistance training reduces postprandial lipaemia in postmenopausal women. J Sports Sci. 2015;33:1890–901. doi:10.1080/02640414.2015.1017732.

31. Cotofana S, Ring-Dimitriou S, Hudelmaier M, Himmer M, Wirth W, Sänger AM, Eckstein F. Effects of exercise intervention on knee morphology in middle-aged women: a longitudinal analysis using magnetic resonance imaging. Cells Tissues Organs. 2010;192:64–72. doi:10.1159/000289816.

32. Deibert P, Solleder F, König D, Vitolins MZ, Dickhuth H-H, Gollhofer A, Berg A. Soy protein based supplementation supports metabolic effects of resistance training in previously untrained middle aged males. Aging Male. 2011;14:273–9. doi:10.3109/13685538.2011.565091.

33. Jong J de, Lemmink KAPM, Stevens M, Greef MHG de, Rispens P, King AC, Mulder T. Six-month effects of the Groningen active living model (GALM) on physical activity, health and fitness outcomes in sedentary and underactive older adults aged 55-65. Patient Educ Couns. 2006;62:132–41. doi:10.1016/j.pec.2005.06.017.

34. Donges CE, Duffield R, Guelfi KJ, Smith GC, Adams DR, Edge JA. Comparative effects of single-mode vs. duration-matched concurrent exercise training on body composition, low-grade inflammation, and glucose regulation in sedentary, overweight, middle-aged men. Appl Physiol Nutr Metab. 2013;38:779–88. doi:10.1139/apnm-2012-0443.

35. Duft RG, Castro A, Bonfante ILP, Brunelli DT, Chacon-Mikahil MPT, Cavaglieri CR. Metabolomics Approach in the Investigation of Metabolic Changes in Obese Men after 24 Weeks of Combined Training. J Proteome Res. 2017;16:2151–9. doi:10.1021/acs.jproteome.6b00967.

36. Elliott KJ, Sale C, Cable NT. Effects of resistance training and detraining on muscle strength and blood lipid profiles in postmenopausal women. Br J Sports Med. 2002;36:340–4. doi:10.1136/bjsm.36.5.340.

37. Figueroa A, Park SY, Seo DY, Sanchez-Gonzalez MA, Baek YH. Combined resistance and endurance exercise training improves arterial stiffness, blood pressure, and muscle strength in postmenopausal women. Menopause. 2011;18:980–4. doi:10.1097/gme.0b013e3182135442.

38. Figueroa A, Kalfon R, Madzima TA, Wong A. Whole-body vibration exercise training reduces arterial stiffness in postmenopausal women with prehypertension and hypertension. Menopause. 2014;21:131–6. doi:10.1097/GME.0b013e318294528c.

39. Flandez J, Belando N, Gargallo P, Fernández-Garrido J, Vargas-Foitzick RA, Devis-Devis J, Colado JC. Metabolic and Functional Profile of Premenopausal Women With Metabolic Syndrome After Training With Elastics as Compared to Free Weights. Biol Res Nurs. 2017;19:190–7. doi:10.1177/1099800416674307.

40. Fu S, Choy NL, Nitz J. Controlling balance decline across the menopause using a balance-strategy training program: a randomized, controlled trial. Climacteric. 2009;12:165–76. doi:10.1080/13697130802506614.

41. Gillett PA, Caserta MS, White AT, Martinson L. Responses of 49- to 59-Year-Old Sedentary, Overweight Women to Four Months of Exercise Conditioning and/or Fitness Education. Activities, Adaptation & Aging. 1995;19:13–32. doi:10.1300/J016v19n04_02.

42. Granacher U, Wick C, Rueck N, Esposito C, Roth R, Zahner L. Promoting balance and strength in the middle-aged workforce. Int J Sports Med. 2011;32:35–44. doi:10.1055/s-0030-1267214.

43. Holviala J, Häkkinen A, Alen M, Sallinen J, Kraemer W, Häkkinen K. Effects of prolonged and maintenance strength training on force production, walking, and balance in aging women and men. Scand J Med Sci Sports. 2014;24:224–33. doi:10.1111/j.1600-0838.2012.01470.x.

44. Janzen CL, Chilibeck PD, Davison KS. The effect of unilateral and bilateral strength training on the bilateral deficit and lean tissue mass in post-menopausal women. Eur J Appl Physiol. 2006;97:253–60. doi:10.1007/s00421-006-0165-1.

45. Jørgensen MB, Ektor-Andersen J, Sjøgaard G, Holtermann A, Søgaard K. A randomised controlled trial among cleaners--effects on strength, balance and kinesiophobia. BMC Public Health. 2011;11:776. doi:10.1186/1471-2458-11-776.

46. Kak H-B, Cho S-H, Lee Y-H, Cho B-J, Kim J-W, Oh B-D, Koh H-W. A study of effect of the compound physical activity therapy on muscular strength in obese women. J Phys Ther Sci. 2013;25:1039–41. doi:10.1589/jpts.25.1039.

47. Karatrantou K, Gerodimos V, Häkkinen K, Zafeiridis A. Health-Promoting Effects of Serial vs. Integrated Combined Strength and Aerobic Training. Int J Sports Med. 2017;38:55–64. doi:10.1055/s-0042-116495.

48. Karavirta L, Häkkinen A, Sillanpää E, García-López D, Kauhanen A, Haapasaari A, et al. Effects of combined endurance and strength training on muscle strength, power and hypertrophy in 40-67-year-old men. Scand J Med Sci Sports. 2011;21:402–11. doi:10.1111/j.1600-0838.2009.01059.x.

49. Kim S, Bemben MG, Bemben DA. Effects of an 8-month yoga intervention on arterial compliance and muscle strength in premenopausal women. J Sports Sci Med. 2012;11:322–30.

50. Klentrou P, Slack J, Roy B, Ladouceur M. Effects of exercise training with weighted vests on bone turnover and isokinetic strength in postmenopausal women. J Aging Phys Act. 2007;15:287–99. doi:10.1123/japa.15.3.287.

51. Kloubec JA. Pilates for improvement of muscle endurance, flexibility, balance, and posture. Journal of Strength and Conditioning Research. 2010;24:661–7. doi:10.1519/JSC.0b013e3181c277a6.

52. Kostić R, Uzunović S, Purenović-Ivanović T, Miletić Đ, Katsora G, Pantelić S, Milanović Z. The effects of dance training program on the postural stability of middle aged women. Cent Eur J Public Health. 2015;23 Suppl:S67-73. doi:10.21101/cejph.a4206.

53. Libardi C, Bonganha V, Conceicao M, Souza G de, Bernardes C, Secolin R, et al. The periodized resistance training promotes similar changes in lipid profile in middle-aged men and women. The Journal of sports medicine and physical fitness. 2012;52:289–92.

54. Augusto Libardi C, Bonganha V, Soares Conceição M, Vergínia De Souza G, Fernandes Bernardes C, Secolin R, et al. The periodized resistance training promotes similar changes in lipid profile in middle-aged men and women. J SPORTS MED PHYS FITNESS. 2012;52:286–92.

55. Liphardt AM, Schipilow J, Hanley DA, Boyd SK. Bone quality in osteopenic postmenopausal women is not improved after 12 months of whole-body vibration training. Osteoporos Int. 2015;26:911–20. doi:10.1007/s00198-014-2995-8.

56. Marcus RL, Lastayo PC, Dibble LE, Hill L, McClain DA. Increased strength and physical performance with eccentric training in women with impaired glucose tolerance: a pilot study. J Womens Health (Larchmt). 2009;18:253–60. doi:10.1089/jwh.2007.0669.

57. Marston KJ, Peiffer JJ, Rainey-Smith SR, Gordon N, Teo SY, Laws SM, et al. Resistance training enhances delayed memory in healthy middle-aged and older adults: A randomised controlled trial. J Sci Med Sport. 2019;22:1226–31. doi:10.1016/j.jsams.2019.06.013.

58. Del Moreno-Muñoz MM, Hita-Contreras F, Estudillo-Martínez MD, Aibar-Almazán A, Castellote-Caballero Y, Bergamin M, et al. The Effects of Abdominal Hypopressive Training on Postural Control and Deep Trunk Muscle Activation: A Randomized Controlled Trial. International Journal of Environmental Research and Public Health. 2021;18:2741. doi:10.3390/ijerph18052741.

59. Nelson ME, Fiatarone MA, Morganti CM, Trice I, Greenberg RA, Evans WJ. Effects of high-intensity strength training on multiple risk factors for osteoporotic fractures. A randomized controlled trial. JAMA. 1994;272:1909–14. doi:10.1001/jama.1994.03520240037038.

60. Neves LM, Fortaleza AC, Rossi FE, Diniz TA, Codogno JS, Gobbo LA, et al. Functional training reduces body fat and improves functional fitness and cholesterol levels in postmenopausal women: a randomized clinical trial. J SPORTS MED PHYS FITNESS. 2017;57:448–56. doi:10.23736/S0022-4707.17.06062-5.

61. Nunes PRP, Oliveira AA, Martins FM, Souza AP, Orsatti FL. Effect of resistance training volume on walking speed performance in postmenopausal women: A randomized controlled trial. Exp Gerontol. 2017;97:80–8. doi:10.1016/j.exger.2017.08.011.

62. Palumbo MV, Wu G, Shaner-McRae H, Rambur B, McIntosh B. Tai Chi for older nurses: a workplace wellness pilot study. Appl Nurs Res. 2012;25:54–9. doi:10.1016/j.apnr.2010.01.002.

63. Paoli A, Pacelli F, Bargossi AM, Marcolin G, Guzzinati S, Neri M, et al. Effects of three distinct protocols of fitness training on body composition, strength and blood lactate. J SPORTS MED PHYS FITNESS. 2010;50:43–51.

64. Park S-M, Kwak Y-S, Ji J-G. The Effects of Combined Exercise on Health-Related Fitness, Endotoxin, and Immune Function of Postmenopausal Women with Abdominal Obesity. J Immunol Res. 2015;2015:830567. doi:10.1155/2015/830567.

65. Perchthaler D, Grau S, Hein T. Evaluation of a six-week whole-body vibration intervention on neuromuscular performance in older adults. Journal of Strength and Conditioning Research. 2015;29:86–95. doi:10.1519/JSC.0000000000000608.

66. Reis JG, Costa GC, Schmidt A, Ferreira CHJ, Abreu DCC. Do muscle strengthening exercises improve performance in the 6-minute walk test in postmenopausal women? Rev Bras Fisioter. 2012;16:236–40. doi:10.1590/s1413-35552012005000022.

67. Sallinen J, Fogelholm M, Volek JS, Kraemer WJ, Alen M, Häkkinen K. Effects of strength training and reduced training on functional performance and metabolic health indicators in middle-aged men. Int J Sports Med. 2007;28:815–22. doi:10.1055/s-2007-964901.

68. Shaw BS, Gouveia M, McIntyre S, Shaw I. Anthropometric and cardiovascular responses to hypertrophic resistance training in postmenopausal women. Menopause. 2016;23:1176–81. doi:10.1097/GME.0000000000000687.

69. Shirazi KK, Wallace LM, Niknami S, Hidarnia A, Torkaman G, Gilchrist M, Faghihzadeh S. A home-based, transtheoretical change model designed strength training intervention to increase exercise to prevent osteoporosis in Iranian women aged 40-65 years: a randomized controlled trial. Health Educ Res. 2007;22:305–17. doi:10.1093/her/cyl067.

70. Singh JA, Schmitz KH, Petit MA. Effect of resistance exercise on bone mineral density in premenopausal women. Joint Bone Spine. 2009;76:273–80. doi:10.1016/j.jbspin.2008.07.016.

71. Sipilä S, Taaffe DR, Cheng S, Puolakka J, Toivanen J, Suominen H. Effects of hormone replacement therapy and high-impact physical exercise on skeletal muscle in post-menopausal women: a randomized placebo-controlled study. Clinical Science. 2001;101:147–57.

72. Smith MF, Ellmore M, Middleton G, Murgatroyd PM, Gee TI. Effects of Resistance Band Exercise on Vascular Activity and Fitness in Older Adults. Int J Sports Med. 2017;38:184–92. doi:10.1055/s-0042-121261.

73. Taaffe D, Sipilä S, Cheng S, Puolakka J, Toivanen J, SUOMINEN H. The effect of hormone replacement therapy and/or exercise on skeletal muscle attenuation in postmenopausal women: a yearlong intervention. Clin Physiol Funct Imaging. 2005;25:297–304. doi:10.1111/j.1475-097X.2005.00628.x.

74. Tsourlou T, Gerodimos V, Kellis E, Stavropoulos N, Kellis S. The Effects of a Calisthenics and a Light Strength Training Program on Lower Limb Muscle Strength and Body Composition in Mature Women. Journal of Strength and Conditioning Research. 2003;17:590. doi:10.1519/1533-4287(2003)017<0590:TEOACA>2.0.CO;2.

75. Uusi-Rasi K, Kannus P, Cheng S, Sievänen H, Pasanen M, Heinonen A, et al. Effect of alendronate and exercise on bone and physical performance of postmenopausal women: a randomized controlled trial. Bone. 2003;33:132–43. doi:10.1016/S8756-3282(03)00082-6.

76. Wen HJ, Huang TH, Li TL, Chong PN, Ang BS. Effects of short-term step aerobics exercise on bone metabolism and functional fitness in postmenopausal women with low bone mass. Osteoporos Int. 2017;28:539–47. doi:10.1007/s00198-016-3759-4.

77. Wittke A, Stengel S von, Hettchen M, Fröhlich M, Giessing J, Lell M, et al. Protein Supplementation to Augment the Effects of High Intensity Resistance Training in Untrained Middle-Aged Males: The Randomized Controlled PUSH Trial. Biomed Res Int. 2017;2017:3619398. doi:10.1155/2017/3619398.

78. Wong A, Figueroa A, Son W-M, Chernykh O, Park S-Y. The effects of stair climbing on arterial stiffness, blood pressure, and leg strength in postmenopausal women with stage 2 hypertension. Menopause. 2018;25:731–7. doi:10.1097/GME.0000000000001072.

79. Zhao J, Zhang L, Tian Y. Effect of 6 months of Tai Chi Chuan and calcium supplementation on bone health in females aged 50–59 years. J Exerc Sci Fit. 2007;5.
